# The structure of the dopamine D_3_ receptor bound to cariprazine reveals principles for partial agonists with designed pharmacology

**DOI:** 10.1126/sciadv.aef7411

**Published:** 2026-08-28

**Authors:** Eliane Hadas Yardeni, Dóra Judit Kiss, Julie Sanchez, Keshet Shavit, Dénes Szepesi Kovács, Attila Egyed, Caleb D. Vogt, Supriya A. Gaitonde, Jacqueline Glenn, Meritxell Canals, Michel Bouvier, Amy Hauck Newman, J. Robert Lane, György M. Keserű, Moran Shalev-Benami

**Affiliations:** ^1^ Department of Chemical and Structural Biology, Weizmann Institute of Science, Rehovot, Israel.; ^2^ Department of Life Sciences Core Facilities, Weizmann Institute of Science, Rehovot, Israel.; ^3^ Medicinal Chemistry Research Group, HUN-REN Research Centre for Natural Sciences, Budapest, Hungary.; ^4^ National Laboratory of Drug Research and Development, Budapest, Hungary.; ^5^ Division of Physiology, Pharmacology and Neuroscience, School of Life Sciences, Queen’s Medical Centre, University of Nottingham, Nottingham, UK.; ^6^ Centre of Membrane Proteins and Receptors, University of Birmingham and University of Nottingham, Nottingham, UK.; ^7^ Molecular Targets and Medications Discovery Branch, National Institute on Drug Abuse–Intramural Research Program, National Institutes of Health, Baltimore, MD, USA.; ^8^ Institute for Research in Immunology and Cancer, Department of Biochemistry and Molecular Medicine, Université de Montréal, Montréal, QC, Canada.

## Abstract

The third-generation antipsychotic cariprazine is a low-efficacy partial agonist of the dopamine D_3_ receptor (D_3_R). Here, we report the cryo–electron microscopy structure of cariprazine bound to D_3_R, establishing a framework for understanding ligand recognition in this receptor. We further determine structures of D_3_R in complex with a series of cariprazine derivatives spanning inverse agonists to high-efficacy partial agonists. Integration of structural data with pharmacological profiling and molecular dynamics simulations reveals how subtle chemical modifications translate into distinct functional outcomes. Determinants distinguishing agonism from inverse agonism are well defined, whereas differences among partial agonists arise from small positional shifts of the ligand within the orthosteric binding site. In contrast, the extended binding site primarily modulates ligand stability, affinity, and receptor selectivity. These findings establish a mechanistic link between bitopic ligand architecture and receptor activation, providing a “ligand-centric” view of D_3_R signaling. Leveraging these principles, we designed and validated cariprazine derivatives with enhanced D_3_/D_2_ selectivity and partial agonist activity. Together, this work provides a structural and pharmacological blueprint for the rational design of D_3_R-targeting ligands with tailored efficacy and therapeutic profiles.

## INTRODUCTION

The neurotransmitter dopamine exerts its actions through five G protein–coupled dopamine receptors (DRs) (*1*). D_1_-like (D_1_ and D_5_) receptors couple to the stimulatory G_s/olf_ heterotrimeric G proteins and the D_2_-like (D_2_ to D_4_) activate the G_i/o_ pathways. The human dopamine D_3_ receptor (hD_3_R) displays high sequence identity with the hD_2_R [58.5% increasing to >80% within the transmembrane domains (TMs)] but has a distinct pattern of expression in the human brain (*2*–*10*). D_3_R has been implicated in functions such as movement control, cognition, learning, reward, emotional regulation, and social behavior (*11*). Selective D_3_R antagonists and partial agonists have been proposed for the treatment of multiple central nervous system disorders (*11*–*13*). Compounds with a polypharmacological profile but substantial D_3_R affinity have shown clinical efficacy. This is exemplified by cariprazine (RGH-188), a Food and Drug Administration–approved medication for the treatment of schizophrenia, full spectrum of bipolar disorder, and major depressive disorder (*14*). As a high-affinity, low–intrinsic efficacy partial agonist of the D_2/3_Rs, cariprazine is classed as a third-generation antipsychotic with a distinct mechanism of action compared to the D_2_R antagonism of first- and second- generation antipsychotics (*15*–*17*). Low-efficacy partial D_2/3_ R agonists act as functional antagonists in the dorsal striatum where excessive dopamine activity is thought to cause positive symptoms, reducing DR signaling down to the partial agonist’s intrinsic efficacy (*15*, *18*). In the mesocortical pathway, where low dopamine is associated with negative symptoms and cognitive impairment, D_2/3_R partial agonism may reverse these symptoms (*18*, *19*). Another benefit of partial agonism is that D_2_R signaling is not completely inhibited and, therefore, they offer reduced extrapyramidal side effect profiles as compared to full D_2_R antagonist first- and second-generation antipsychotics, even at high levels of D_2_R occupancy (*18*, *19*). Cariprazine is a D_3_R-preferring partial agonist with ∼3- to 10-fold selectivity for D_3_R over D_2_R (*9*, *17*, *20*). This preference for D_3_R differs from other clinically used DR partial agonists, such as aripiprazole and brexpriprazole (*17*, *21*). Cariprazine has also shown efficacy in full spectrum of bipolar disorders and major depression (*22*–*26*). Preclinical models have provided evidence that partial agonism (as compared to full agonism or antagonism) at the D_3_R is important for this efficacy (*27*–*29*). The degree of intrinsic efficacy of D_2-like_R partial agonists, such as cariprazine, is thought to be key to both their therapeutic efficacy and reduced side effect profile as compared to inverse agonists/antagonists (*16*, *21*, *30*, *31*). Affinity for serotonin 5-hydroxytryptamine receptor 1A (5-HT_1A_R) and 5-HT_2A_R likely also contributes to this therapeutic profile. Thus, the combination of intrinsic efficacy and receptor selectivity across the DRs and beyond underlies the utility of cariprazine across these disease states (*11*, *14*).

The first crystal structure of the D_3_R in complex with the antagonist eticlopride highlighted an orthosteric binding site (OBS) that is highly conserved between the D_2-like_Rs and an extended binding site (EBS), sometimes termed the secondary binding pocket of the D_3_R (*32*, *33*), that extends away from the orthosteric site toward the extracellular receptor facet and the extracellular loop 1 (ECL1) and ECL2 (*33*). Most D_3_R-selective ligands have a common structure consisting of a head group, which is positively charged and bears an aromatic ring (e.g., 2,3-dichlorophenyl piperazine), and an extended aromatic or often heteroaromatic tail (e.g., indole) connected by a linker that has specified length and composition for optimal D_3_R affinity and selectivity (*12*, *34*, *35*). The interaction between the compound terminus and the EBS can confer selectivity over the D_2_R, modulate intrinsic efficacy, and affect binding kinetics (*33*, *36*–*41*).

Cariprazine is the prototypic dopaminergic ligand that, by having an acyclic tail, differs in structure from the canonical pharmacophore of D_2-like_R ligands characterized by heteroaromatic cores in both primary and secondary pharmacophores that are proposed to bind the OBS and EBS, respectively (*42*, *43*). A study in which the structures of serotonin 5-HT_2A_R were determined in complex with cariprazine and the structurally related aripiprazole found that these ligands adopted an unexpected upside-down orientation in which the tail moiety of both ligands extended down vertically into the receptor helical bundle to occupy a hydrophobic “exosite” defined by residues of TM5 and TM6. At the 5-HT_1A_R, however, aripiprazole binds in a similar fashion to the canonical DR agonists, with the pharmacophore shared with cariprazine occupying the OBS and tail extending toward the EBS (*44*). In addition to these conflicting data, the molecular details of optimizing the selectivity and the intrinsic efficacy of these compounds remain unclear. Relatively modest changes in the structure of phenylpiperazine D_3_R-targeted ligands can have profound effects on the selectivity and efficacy of these compounds (*38*–*40*, *42*, *45*). Despite the key role of DRs in the mechanism of action of third-generation antipsychotics (aripiprazole, brexpiprazole, and cariprazine), no structural information is available for the binding of these partial agonists at this receptor family. Given the clinical efficacy of cariprazine in the negative symptoms of schizophrenia, the full spectrum of bipolar disorder, and major depression, we selected D_3_Rs and the cariprazine chemotype to investigate the structural basis of selectivity and efficacy.

Here, we present the cryo–electron microscopy (cryo-EM) structure of cariprazine bound to the D_3_R, indicating a canonical binding mode that is opposite to the 5-HT_2A_R structure. Our pharmacology data support this binding mode. We further show the structures of four compounds with a cariprazine scaffold having variable pharmacological properties spanning from efficacious partial agonists to inverse agonists. Together with pharmacology and molecular dynamics (MD) simulations, these data tell the story of the D_3_R activation from the “ligand” point of view and identify principal elements for drug design. These findings were used in designing partial agonists with improved D_3_/D_2_ selectivity available for the investigation of D_3_R-assisted treatment options in a range of disease models.

## RESULTS AND DISCUSSION

### Cariprazine exhibits a “canonical” binding pose in the D_3_R

The molecular structure of cariprazine comprises a 2,3-dichlorophenyl ring directly linked to a piperazine moiety, which is further connected via an ethyl-cyclohexyl linker to a dimethylurea tail (Fig. 1A). This dichlorophenyl group differs from the catechol ring of the endogenous ligand dopamine both in the substitution of the two hydroxyl groups with chlorine atoms and in their relative orientation within the ring (Fig. 1A). Recent structures of dopamine-bound D_1_R and D_2_R have established a conserved binding mode in which dopamine occupies a cavity formed by TM3, TM5 to TM7, and ECL2 (*46*–*48*). In these structures, the catechol hydroxyl groups engage conserved serine residues in TM5 [S^5.42×43^ and S^5.46×461^; superscript numbers correspond to generic residue numbering: with Ballesteros-Weinstein nomenclature followed by the GPCRdb structure-based position (*49*, *50*)] and a residue at position 6.55 (asparagine in D_1-like_Rs and histidine in D_2-like_Rs). Given the high conservation of OBS residues across DR subtypes (fig. S1), a similar binding mode would be expected for dopamine-like ligands in the D_3_R. However, previously reported D_3_R structures have involved ligands with scaffolds distinct from both dopamine and cariprazine (*41*, *51*, *52*), leaving the binding mode of this clinically relevant compound unresolved.

**Fig. 1.**
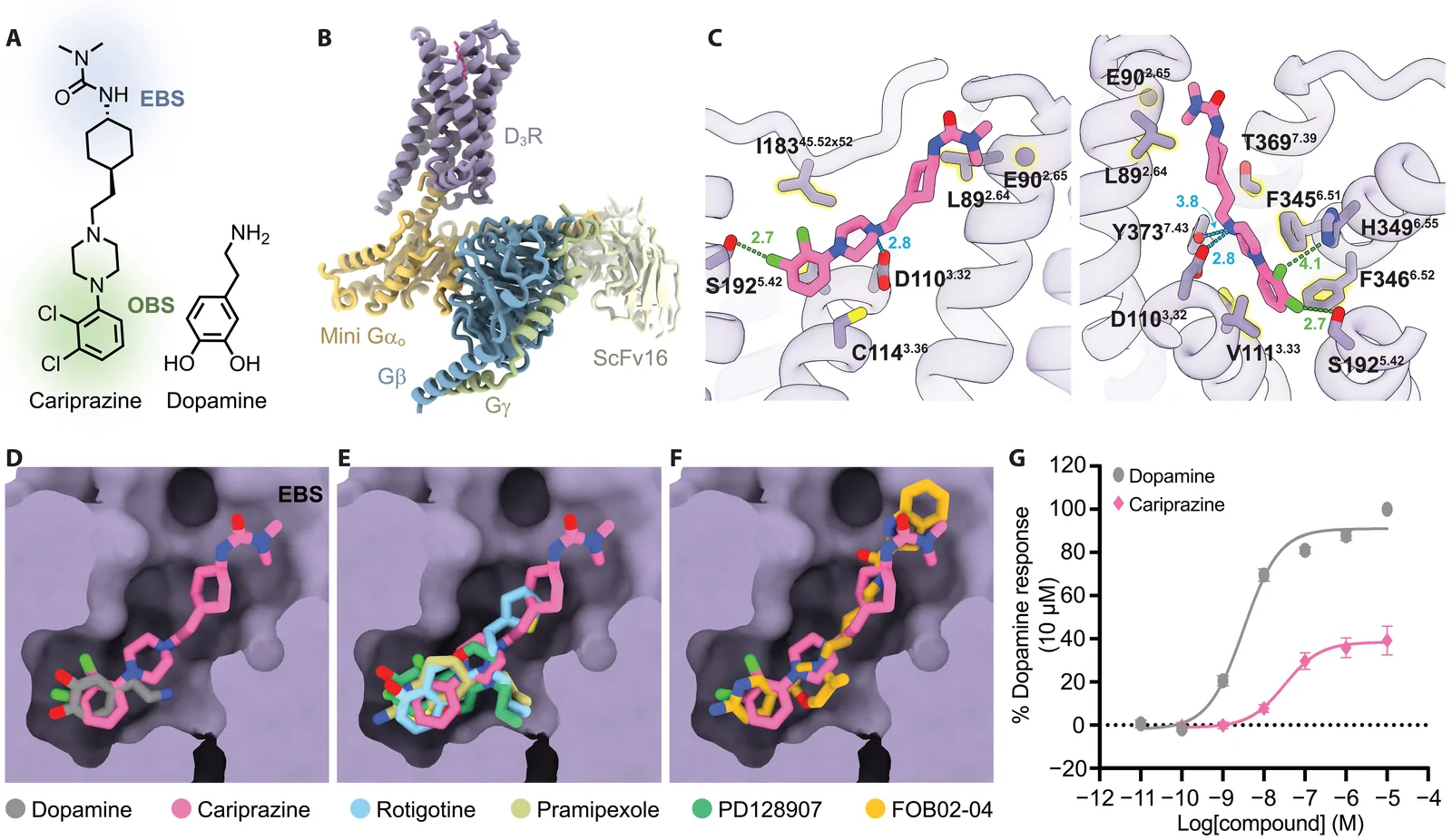
Cryo-EM structures of D_3_R with cariprazine and comparison to other agonists. (**A**) The chemical structures of cariprazine (left) and dopamine (right). The OBS binding region is colored green, and the EBS binding region is colored blue. (**B**) Model of D_3_R-mini G_o_-ScFv complex bound to cariprazine. Receptor is in purple, and G protein heterotrimer is colored yellow, cyan and green for the α, β, and γ subunits, respectively. ScFv16 is in beige. (**C**) Detailed binding interactions between D_3_R and cariprazine. Interacting residues are shown as sticks. Blue dashed lines represent hydrogen bonds, green lines are for halogen interactions, and yellow outline indicates hydrophobic interactions. Residues in a 4-Å vicinity of cariprazine in the EBS are shown. All distances are in angstroms. (**D**) Superimposition of cariprazine (pink) bound to D_3_R (purple, surface) and dopamine (gray) binding pose in D_2_R [PDB 8TZQ (*48*)]. A detailed comparison of the binding modes is in fig. S5A. (**E**) Comparison between the binding pose in D_3_R of cariprazine and the agonists rotigotine [PDB 8IRT (*52*), cyan], pramipexole [PDB 7CMU (*51*), lime green], and PD128907 [PDB 7CMV (*51*), dark green]. (**F**) Superimposition of cariprazine and FOB02-04 [PDB 9F33 (*41*), orange] binding modes. (**G**) Concentration response curves of dopamine (gray) and cariprazine (pink) in a Gα_oA_ G protein activation (GPA) assay. Cariprazine acts as a partial agonist (pEC_50_ = 7.49 ± 0.21, *E*_max_ = 38 ± 2.8) as compared to dopamine (pEC_50_ = 8.49 ± 0.07, *E*_max_ = 100). Data are the means ± SEM of three individual experiments performed in duplicate.

To address this, we determined the structure of cariprazine bound to an activated D_3_R using single-particle cryo-EM. The full-length hD_3_R was coexpressed with mini-Gα_o_βγ and purified in the presence of cariprazine. Complex stabilization was achieved using an enhanced affinity split NanoLuc system (LgBiT-HiBiT) (*53*, *54*), in which the receptor was fused to LgBiT at its C terminus and Gβ was tagged with HiBiT at its N terminus (fig. S2A). In addition, single-chain variable fragment antibody 16 (ScFv16) was used to stabilize the G_i/o_-coupled complex (*55*). The three-dimensional (3D) map was obtained at an overall resolution of 2.9 Å (figs. S2 to S4, and table S1), revealing a well-defined density for the receptor–G protein complex and the bound ligand (Fig. 1B and fig. S4).

Cariprazine adopts a canonical binding mode, with its dichlorophenyl-piperazine core occupying the OBS and the dimethylurea tail extending into the EBS toward the extracellular ends of TM2 and ECL1 (Fig. 1C). Superposition with dopamine-bound receptor structures shows substantial overlap between the catechol ring of dopamine and the dichlorophenyl group of cariprazine. However, because of differences in substitution patterns, only the meta substituents align, whereas the para-hydroxy group of dopamine and the ortho-chloro substituent of cariprazine adopt distinct orientations (Fig. 1D). The meta-chloro group of cariprazine forms a halogen bond with S192^5.42×43^ (Fig. 1C), partially mimicking the hydrogen bond formed by dopamine at this position (*46*–*48*, *56*) (fig. S5A). In contrast, interactions with S196^5.46×46^ and H349^6.55×55^ observed for dopamine are absent in the cariprazine-bound structure. Accordingly, alanine substitutions at these positions markedly reduce dopamine potency but have little effect on cariprazine potency or efficacy (fig. S6 and table S2). Mutation of S192^5.42×43^ similarly causes a strong reduction in dopamine potency but only modestly affects cariprazine efficacy, suggesting that the weaker halogen bond and compensatory interactions within the EBS mitigate the loss of this contact. Together, these differences likely contribute to the partial agonism of cariprazine relative to dopamine (Fig. 1G).

Within the OBS, cariprazine is further stabilized by a salt bridge between the protonated tertiary amine of the piperazine moiety and D110^3.32×32^ (Fig. 1C), a conserved interaction shared among aminergic G protein–coupled receptors (GPCRs) (*46*–*48*). Additional hydrophobic contacts are formed with V111^3.33×33^, C114^3.36×36^, F345^6.51×51^, F346^6.52×52^, and I183^45.52×52^ in ECL2. The hydroxyl group of Y373^7.43×42^ is positioned near the piperazine moiety and may contribute additional polar stabilization (Fig. 1C). Functional assays support the importance of these interactions: Mutation of C114^3.36×36^ (C114A; 5.8-fold decrease in cariprazine potency and a 71% decrease in *E*_max_ relative to dopamine) reduces both potency and efficacy, whereas mutation of Y373^7.43×42^ (Y373F; *E*_max_ = 82.5% of dopamine) converts cariprazine from a partial agonist into an inverse agonist (*E*_max_ = −14.4% of dopamine) (fig. S6 and table S2).

Beyond the OBS, the cyclohexyl-ethyl linker and dimethylurea tail extend into the EBS, a region connecting the orthosteric pocket to the extracellular milieu (Fig. 1, C and D). In this region, cariprazine interacts with residues in TM2 (L89^2.64×630^ and E90^2.65×64^) and TM7 (T369^7.39×38^) (Fig. 1C). Mutations at these positions produce modest increases in potency (fig. S6 and table S2).

Comparison with previously reported agonist-bound D_3_R structures, including complexes with pramipexole (*51*), rotigotine (*52*), PD128907 (*51*), and FOB02-04 (*41*), reveals a conserved anchoring of ligands within the OBS, particularly through the salt bridge with D110^3.32×32^ (Fig. 1, E and F, and fig. S5B). However, unlike dopamine and most full agonists, cariprazine lacks a strong hydrogen bond with S192^5.42×43^ (fig. S5B), instead relying on a weaker halogen interaction (Fig. 1C). This difference may underlie the partial agonism reported for cariprazine compared with the full agonism reported for the other ligands (*51*, *52*, *57*, *58*). Notably, while most DR agonists do not protrude much beyond OBS (Fig. 1E), the bitopic agonist FOB02-04 extends into the same EBS region as cariprazine but forms additional contacts with ECL2 and TM7 (Fig. 1F and fig. S5B) (*41*), resulting in enhanced receptor interactions and higher efficacy (*59*). These observations highlight how subtle differences in orthosteric interactions and extended binding site engagement collectively shape ligand efficacy and subtype selectivity (*41*).

Beyond binding and activating the DRs, cariprazine and cariprazine-like derivatives act as partial agonists at serotonin receptors (5-HTRs) (*60*, *61*). For example, aripiprazole, a D_2/3_R partial agonist antipsychotic, shares the same dichlorophenyl-piperazine scaffold as cariprazine, but its EBS binding moiety is a 1,2,3,4-tetrahydroquinolin-2-one ring connected by an acyclic linker, which differs from the cyclic linker and the acyclic dimethylurea terminus in cariprazine (Fig. 2A). Aripiprazole acts as a partial agonist at the 5-HT_1A_R and a low-efficacy partial agonist at 5-HT_2A_R (*30*, *62*, *63*).

**Fig. 2.**
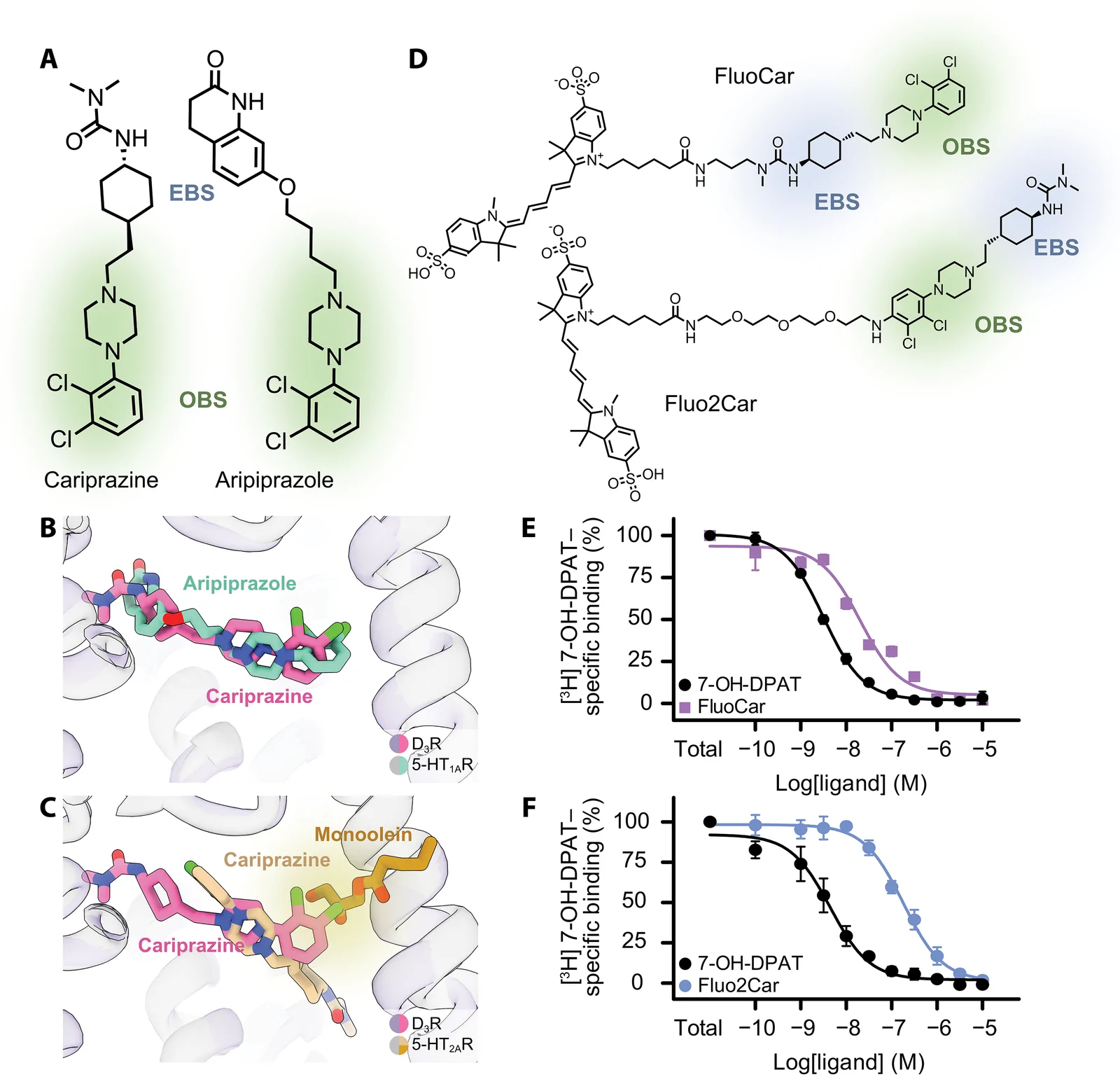
Different binding modes of cariprazine at the D_3_R and 5-HT_2A_R. (**A**) The chemical structures of cariprazine (left) and aripiprazole (right). The two molecules share the same dichlorophenyl-piprazine backbone (highlighted in green), but their secondary pharmacophores greatly differ. (**B**) Binding of cariprazine to D_3_R (pink) compared with aripiprazole binding mode to 5-HT_1A_R [teal; PBD 7E2Z (*44*)]. (**C**) Superimposition of cariprazine binding pose in D_3_R (pink) with 5-HT_2A_R [tan; PDB 7VOD (*60*)]. Cariprazine binds to 5-HT_2A_R in a skewed, upside-down orientation and is accompanied by the lipid monoolein that enters the binding pocket. Steric hindrance between cariprazine in D_3_R and monoolein in 5-HT_2A_R is shown in yellow. (**D**) The chemical structures of the two fluorescent derivatives of cariprazine, FluoCar and Fluo2Car. The OBS (green) and EBS (blue) regions are shown for each structure. (**E** and **F**) The ability of FluoCar (purple squares) (E) and Fluo2Car (blue circles) (F) to displace the radiolabeled agonist [^3^H]7-OH-DPAT at the D_3_R in comparison to 7-OH-DPAT (black circles) was measured in a competition binding experiment (FluoCar, *K*_i_ = 13 ± 0.5 nM; Fluo2Car, *K*_i_ = 104 ± 2.1 nM). Data presented are the means ± SEM of three individual experiments performed in duplicate.

Recently, the structures of aripiprazole bound to the 5-HT_2A_R and the 5-HT_1A_R were reported (*44*, *60*). In the structure of aripiprazole bound to the 5-HT_1A_R, aripiprazole position overlaps with our current pose of cariprazine in D_3_R (Fig. 2B). Unexpectedly, the pose observed for aripiprazole in the 5-HT_2A_R was an upside-down orientation, where the phenylpiperazine moiety points toward TM3 and ECL2, and the dihydrocarbostyril tail points toward TM5 and TM6 (*60*). The structure of cariprazine bound to the 5-HT_2A_R was also reported in the same study, and similar to aripiprazole, cariprazine adopted a skewed upside-down position that greatly differs from the pose observed in the current study for the cariprazine-bound D_3_R (Fig. 2C).

Notably, in the 5-HT_2A_R structures, monoolein, a lipid used for matrix formation for in meso crystallization of membrane proteins, protrudes into the binding pocket through an opening between TM4 and TM5 and forms a H bond with H^6.55×55^ (*60*). The monoolein position clashes with the orthogonal orientation of both cariprazine and aripiprazole (Fig. 2C) and was thus suggested by the authors as an explanation for the reversed mode of binding.

To provide pharmacological evidence toward the binding mode of cariprazine at the D_3_R, we designed two fluorescent derivatives of cariprazine (FluoCar and Fluo2Car; Fig. 2D) connecting a sulfo-Cy5 dye to either the aliphatic tail (secondary pharmacophore) of the drug (FluoCar) or to the aryl-piperazine part (Fluo2Car; fig. S7). Because of the bulky fluorophore, FluoCar is expected to prefer the canonical binding mode directing the tail toward the EBS as observed in the present cariprazine D_3_R structure, whereas Fluo2Car would be expected to preferably bind in the upside-down orientation observed for the 5-HT_2A_R (*60*). Both compounds were tested in [^3^H]7-hydroxy-N,N-di-n-propyl-2-aminotetralin ([^3^H]7-OH-DPAT) radioligand displacement assays against D_3_Rs [inhibition constant (*K*_i_) = 13 ± 0.5 nM and 104 ± 2.1 nM for FluoCar and Fluo2Car, respectively] that confirmed the clear preference of the canonical binding mode having 98.1% Boltzmann population over the upside-down orientation (Fig. 2, E and F). The binding specificity of the ligands was confirmed by directly imaging FluoCar and Fluo2Car in a competition imaging experiment on D_3_R expressing cells using confocal microscopy in which both compounds were displaced with 100 nM unlabeled cariprazine (fig. S8). These results support our structural observations that cariprazine adopts the canonical binding pose at the D_3_R.

### Structural and pharmacological characterization of cariprazine derivatives

Having established the binding mode of cariprazine at the D_3_R, we selected a series of analogs spanning efficacious agonists to inverse agonists (Fig. 3A) to examine how structural variations influence binding and intrinsic efficacy. Our primary objective was to dissect the respective contributions of the primary and secondary pharmacophores to ligand function while keeping their chemotype and the linker region unchanged.

**Fig. 3.**
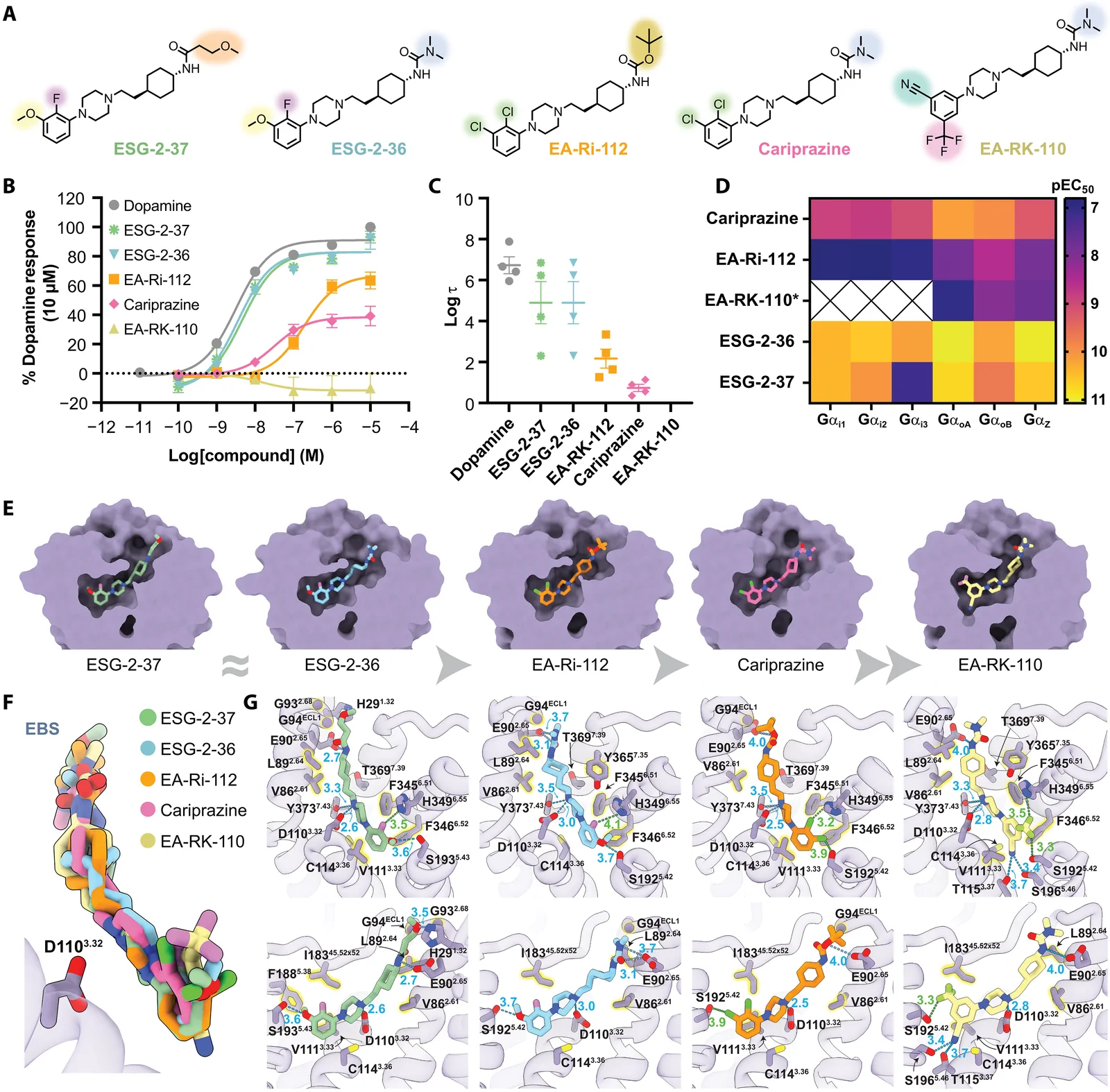
Structural and pharmacological characterization of cariprazine derivatives. (**A**) Chemical structures of cariprazine and derivatives used in this study. Colors indicate ligand derivatization by various moieties (**B**) The ability of dopamine (gray circles), cariprazine (pink diamonds), EA-RK-110 (yellow triangles), EA-Ri-112 (orange squares), ESG-2-36 (blue triangles), and ESG-2-37 (green star) to stimulate Gα_oA_ G protein activation (GPA). Data are the means ± SEM of three individual experiments performed in duplicate. (**C**) The relative efficacy log(τ) of the different D_3_R agonists to stimulate Gα_oA_ GPA as determined using receptor depletion and an operational model of agonism to derive values of τ (fig. S9 and table S3). (**D**) Heatmap illustrating the potency of different agonists and the inverse agonist EA-RK-110 labeled with an asterisk in an assay measuring the D_3_R activation of each of the Gα_i/o/z_ G proteins in an EMTA BRET G protein profiling assay (fig. S11 and table S5). (**E**) Representation of ligands in their respective binding pocket in D_3_R. Ligands are ordered according to their ability to induce receptor agonism. With ESG-2-37 (left) having the highest efficacy, and EA-RK-110 (right) acting as an inverse agonist. (**F**) Superimposition of all ligands, with residue D110^3.32×32^ shown as reference. EBS is indicated. (**G**) Detailed binding interactions between D_3_R and EA-Ri-112 (orange), ESG-2-36 (blue), ESG-2-37 (green), and EA-RK-110 (khaki). Interacting residues are shown as sticks. Blue dashed lines represent hydrogen/electrostatic bonds, green dashed lines represent halogen bonds, and yellow outline represents hydrophobic interactions. All distances are in angstroms.

To quantify their relative intrinsic activity at the D_3_R, we combined a highly sensitive bioluminescence resonance energy transfer (BRET) assay of Gα_oA_ activation (GPA), measuring Gβγ release, with a receptor depletion approach (fig. S8). Cariprazine acts as a high-affinity (*K*_A_ = 35 nM; table S3), low-efficacy partial agonist at the D_3_R (τ = 0.73, 10.9% of dopamine’s intrinsic efficacy; Fig. 3, B and C), consistent with its weak partial agonism observed in adenosine 3′,5′-monophosphate (cAMP) assays (*42*).

Selective modification of the secondary pharmacophore, replacing the dimethylurea group with a *tert*-butoxycarbonyl moiety (EA-Ri-112; Fig. 3A), increased efficacy while retaining partial agonism (*45*, *64*) as compared to cariprazine (τ = 2.16, 31% of dopamine intrinsic efficacy; Fig. 3, B and C, and table S3). In contrast, altering the primary pharmacophore to a 2-fluoro-3-methoxyphenyl piperazine while maintaining the original *N*,*N*-dimethylcarbonyl group (ESG-2-36; Fig. 3A) (*20*) further enhanced efficacy (τ = 4.90, 73% dopamine intrinsic efficacy; Fig. 2, B and C, and table S3).

Notably, substitution of the same pharmacophore with a bulkier primary pharmacophore, 3-cyano-5-trifluoromethylphenyl piperazine (EA-RK-110; Fig. 3A) (*42*), shifted activity toward inverse agonism (Fig. 3B and table S3). Last, simultaneous modification of both pharmacophore elements (ESG-2-37; Fig. 3A) (*20*), combining a 2-fluoro-3-methoxyphenyl piperazine with a 2-methoxyethyl ketone secondary group, yielded an efficacious partial agonist (τ = 4.56, 68% of dopamine intrinsic efficacy, Fig. 3, B and C, and table S3).

Across the series, all ligands displayed lower efficacy at the D_2_R relative to the D_3_R, with the exception of EA-RK-110, which exhibited weak partial agonism rather than inverse agonism at the D_2_R (figs. S9 and S10 and table S4). Time-resolved fluorescence resonance energy transfer (TR-FRET) binding assays revealed consistently high D_3_R affinity across all derivatives (1.1 to 43.6 nM), with cariprazine displaying the highest affinity (table S3).

Some GPCR agonists may display differential effects across different G protein signaling pathways. To assess this possibility for the D_3_R, we profiled all compounds across multiple G protein pathways using an EMTA (effectors to the plasma membrane) BRET assay in both agonist and antagonist modes. The partial agonists cariprazine and EA-Ri-112 displayed the highest potency and efficacy at the at the preferentially coupled Gα_o_ and Gα_z_ pathways (Fig. 3D, fig. S11, and table S5), consistent with previous reports indicating that D_3_R signaling is biased toward Gα_o/z_ (*65*, *66*). In contrast, ESG-2-36 and ESG-2-37 exhibited higher potency and efficacy across all tested pathways, in agreement with the receptor depletion experiments described above (Fig. 3D, fig. S11, and table S5).

Notably, cariprazine and EA-Ri-112 share an identical OBS binding arylpiperazine moiety (2,3-dichlorophenyl), whereas ESG-2 derivatives incorporate a modified arylpiperazine bearing fluoro and methoxy substituents at the ortho and meta positions, respectively (Fig. 3A). Together with variation in the secondary pharmacophore, these observations indicate that ligand efficacy is primarily governed by interactions within the OBS (*67*).

To elucidate the structural basis underlying these pharmacological differences, we determined cryo-EM structures of three agonists (EA-Ri-112, ESG-2-36, and ESG-2-37) and of inverse agonist EA-RK-110 bound to D_3_R. For agonist-bound complexes, the receptor was coupled to Gα_o_-βγ (figs. S2 to S4), reflecting a signaling pathway common to all derivatives (Fig. 3D). Although activity across Gα_i/o/z_ proteins was largely consistent (Fig. 3D, fig. S11, and table S5), we cannot exclude potential differences when D_3_R engages alternative G proteins. For inverse agonist EA-RK-110, we used a BRIL fusion strategy previously used for inactive GPCR structures (*68*, *69*), in which intracellular loop 3 (ICL3) was replaced with BRIL and the complex was stabilized using an anti-BRIL Fab (fig. S2, F and H). This system served as a fiducial marker to facilitate particle alignment during cryo-EM processing (fig. S2F). Cryo-EM studies maps at nominal resolutions of 3.03, 2.85, 2.74, and 3.58 Å for EA-Ri-112-G_o_, ESG-2-36-G_o_, ESG-2-37-G_o_, and EA-RK-110-BRIL, respectively (figs. S2 and S3 and table S1). These maps enabled modeling of all complex components and confident placement of ligands within the binding pocket (Fig. 3, E to G, and fig. S4).

Structural superposition revealed a conserved binding orientation across all ligands. The phenylpiperazine moiety overlaps with the orthosteric position of dopamine, while the secondary pharmacophore extends into the EBS (Fig. 3, E and F). The conserved salt bridge between the protonated amine of the piperazine ring and D110^3.32×32^ was observed in all complexes (Fig. 3, F and G), consistent with extensive structure-activity relationship (SAR) data across aminergic receptors (*33*, *41*, *51*, *52*, *57*, *70*–*74*). In addition, Y373^7.43×42^ forms electrostatic interactions with the ligand linker in all derivatives (Fig. 3G).

Our mutational studies show that, similar to cariprazine, a Y373^7.43×42^F substitution reduced EA-Ri-112 potency by 7.7-fold and decreased *E*_max_ from 96 to 25.5% of dopamine (fig. S12 and table S6). In contrast, the potent agonists ESG-2-36 and ESG-2-37 exhibited substantial potency reductions (27- and 4-fold, respectively) but only modest decreases in efficacy (∼15%). Notably, this mutation enhances inverse agonism of EA-RK-110 (fig. S12 and tables S6 and S7), and our analysis indicates that this might be due to elevation of the receptor constitutive activity by this mutation, as the structurally distinct inverse agonist haloperidol also displays an enhanced level of inverse agonism at this mutant relative to wild-type (WT) (fig. S13). This effect may also explain why cariprazine, which is a weak agonist, behaves as an inverse agonist in this mutant.

Within the OBS, the agonists engage conserved residues S192^5.42×43^ (or S193^5.43×44^ in ESG-2-37) and H349^6.55×55^, mimicking key interactions of dopamine (Fig. 3G). However, differences in phenyl ring substitution modulate interaction strength. EA-Ri-112 forms relatively weak halogen bonds via its dichloro substituents, similar to cariprazine. In contrast, ESG-2-36 and ESG-2-37 establish stronger hydrogen bonds, with the ortho-fluoro substituent interacting with H349^6.55×55^ and the meta-methoxy group engaging S192^5.42×43^ or S193^5.43×44^ (Fig. 3G). These stronger polar interactions likely contribute to their higher efficacy relative to cariprazine and EA-Ri-112. Consistent with this, mutation of OBS residues had limited effects on ligand pharmacology, with the exception of S192^5.42×43^A, which selectively reduced EA-Ri-112 potency (fig. S12 and tables S2 and S6).

Beyond the OBS, all ligands share an ethylcyclohexyl linker but differ in their secondary pharmacophore (Fig. 3A). ESG-2-36 retains the dimethylurea tail of cariprazine, EA-Ri-112 contains a bulky *tert*-butyl group, and ESG-2-37 incorporates a flexible 3-methoxy-*N*-methylpropanamide that extends further toward the extracellular surface (Fig. 3A). A common feature among agonists is a hydrogen bond between the amide moiety and E90^2.65×64^ (Fig. 3G), which is absent in cariprazine due to local side-chain flexibility. In addition, ESG-2-37 forms a distinct hydrogen bond between its 3-methoxy extension and H29^1.32×32^ likely contributing to its higher potency and affinity (Fig. 3, B and C) (*41*). Mutations in EBS residues generally had minimal impact on pharmacology (fig. S12 and tables S6 and S7), suggesting that the bitopic nature of these ligands enables compensation between binding sites.

To complement the structural analysis, we performed MD simulations using the cryo-EM structures as starting points. Both receptors and ligands remained stable throughout the simulations (figs. S14 and S15), and ligand poses closely resembled the experimental structures (fig. S16). EA-Ri-112, which contains the bulkiest secondary group, exhibited reduced interactions with TM2 and increased flexibility within the extracellular vestibule (figs. S16G and S17). In contrast, ESG-2-37 displayed the highest stability across both the OBS and EBS (fig. S16I). These findings correlate with pharmacological data, where EA-Ri-112 is a weak partial agonist and ESG-2-37 is a highly efficacious agonist, suggesting that overall ligand stability in the active receptor conformation, including within the EBS, contributes to efficacy. For the inverse agonist EA-RK-110, MD simulations revealed increased flexibility of the orthosteric moiety, with a tendency to reorient toward TM6 (fig. S16, K and L). This behavior may reflect a structural mechanism by which orthosteric interactions influence the balance between agonism and inverse agonism.

### Structural basis of inverse agonism by EA-RK-110

EA-RK-110 is a D_3_R inverse agonist that shares the same overall scaffold as cariprazine, differing primarily in substitutions on the OBS phenyl ring (Fig. 3A). Specifically, it contains nitrile and trifluoromethyl groups at the meta positions, in contrast to the chloro, fluoro, or methoxy substituents found in the agonists at the ortho and meta positions (Fig. 3, A and F). The trifluoromethyl group engages S192^5.42×43^ and H349^6.55×55^ through polar interactions, contacting the same OBS residues observed in agonist bound structures (Fig. 3G). An additional interaction is formed between the fluoro substituent and I183^45.52×52^.

A key distinction between agonist- and inverse agonist–bound structures lies in the positioning of the phenyl ring within the OBS (Fig. 4A). The presence of the nitrile group at the meta position displaces the ring toward the upper region of the binding pocket, inducing pronounced conformational rearrangements in surrounding residues. The rotamer orientations of S192^5.42×43^ and H349^6.55×55^ differ markedly from those observed in agonist-bound states (Fig. 4B), resulting in inward shifts of the extracellular ends of their respective helices (fig. S18A). Consistent with these structural observations, mutations S192^5.42×43^A and H349^6.55×55^A convert EA-RK-110 from an inverse agonist to a weak partial agonist, with only modest effects on potency (fig. S12 and table S6). In contrast, these mutations do not alter the activity of other bitopic ligands (fig. S12) but do affect dopamine signaling (fig. S6).

**Fig. 4.**
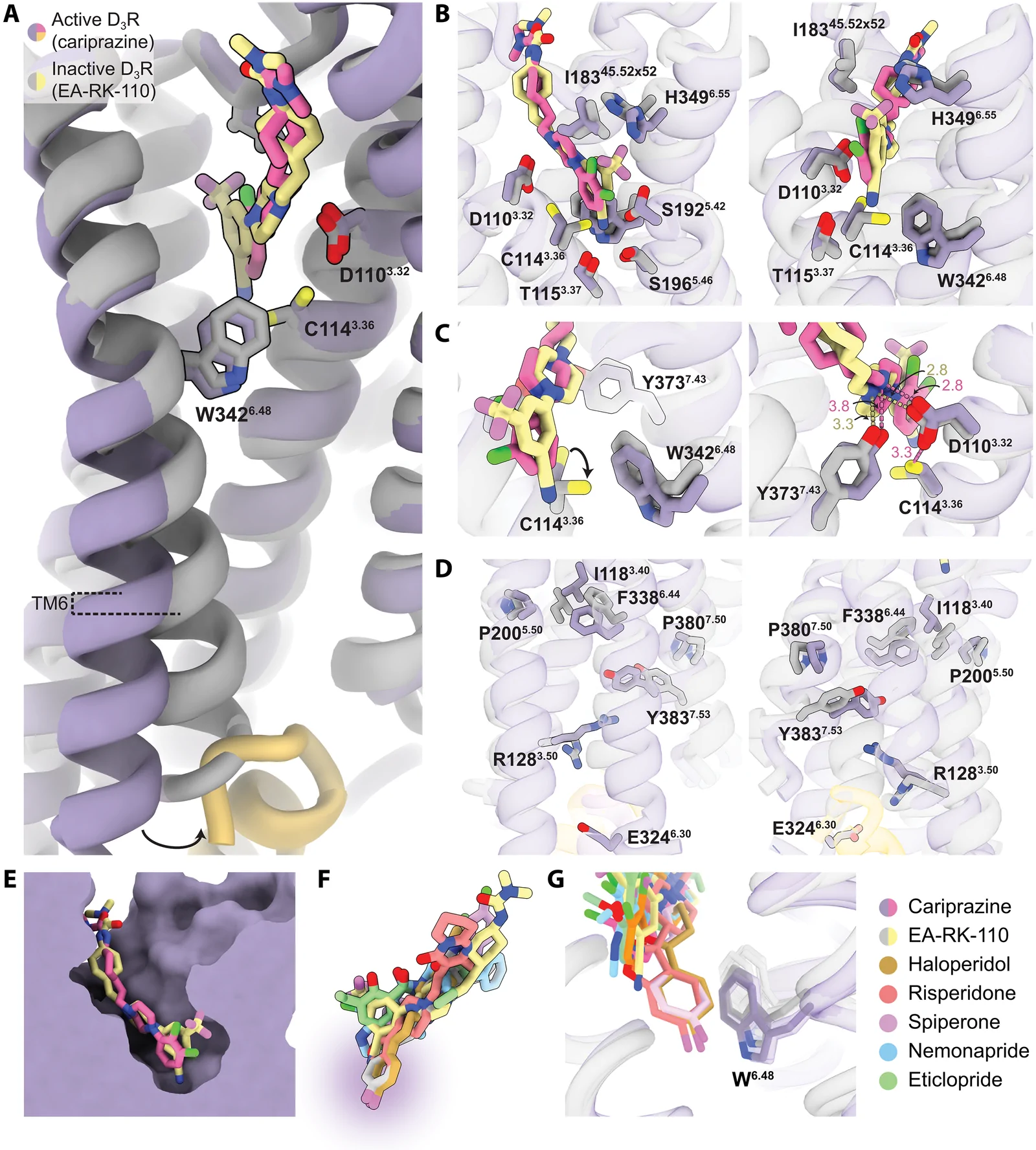
Comparison between the agonist and inverse agonist bound D_3_R. (**A**) View of TM6 in the active (purple) and inactive (gray) states of D_3_R. Structures are of cariprazine (pink) as representative of the active state and EA-RK-110 (light yellow) for inactive. When activated, TM6 shifts outward, enabling the binding of Gα_o_ (gold). (**B**) View of the residues in the binding pocket of D_3_R in the active (purple) and inactive (gray) states. The trifluoromethyl of EA-RK-110 (light yellow) clashes with H349^6.55×55^, resulting in a shift of the helix 6. Furthermore, C114^3.36×36^ interacts with the acetonitrile group of the antagonist, causing it to turn away from D110^3.32×32^ and to interact with W342^6.48×48^. (**C**) A detailed comparison between the active (purple) and inactive (gray) interactions in the binding site. (**D**) Residues of the P^5.50×50^I^3.40×40^F^6.44×44^ motif, the ionic lock motif and the NP^7.50×50^XXY^7.53×53^ motif, shown for the active (purple) and inactive (gray) states. (**E**) Comparison between cariprazine (pink) and EA-RK-110 (light yellow) in the binding pocket of the receptor. The inverse agonist penetrates deeper into the binding site. (**F**) Comparison between EA-RK-110 and other inverse agonists, including haloperidol [PDB 6LUQ (*70*), light brown], risperidone [PDB 6CM4 (*74*), dark pink], spiperone [PDB 7DFP (*72*), purple], nemonapride [PDB 5WIU and 5WIV (*73*), blue], and eticlopride [PDB 3PBL (*33*), green]. Extended moieties that penetrate deeper into the binding site are marked. (**G**) Residue W342^6.48×48^ in the active state (purple) compared to the inactive state (gray).

The nitrile group adopts a downward orientation toward the intracellular side of the receptor, forming interactions with C114^3.36×36^, T115^3.37×37^, and S196^5.46×461^ (Figs. 3G and 4C). The latter two residues are conserved across DRs and are known to mediate ligand binding (*46*–*48*, *56*, *75*, *76*). In agreement, alanine substitutions at T115^3.37×37^ and S196^5.46×461^ significantly impair dopamine potency (fig. S6 and table S2) yet have minimal effects on most bitopic ligands, with the exception of ESG-2-37, which shows reduced potency at S196^5.46×461^A (fig. S12 and tables S6 and S7). In contrast, mutation of C114^3.36×36^A reduces both potency and *E*_max_ of cariprazine and EA-Ri-112 (figs. S6 and S12 and tables S2, S6, and S7), suggesting a broader role for this position in modulating receptor activation.

Residue C^3.36×36^ is a hallmark residue of D_2-like_Rs and is replaced by serine (S^3.36×36^) in D_1_-like subtypes. In the inverse agonist–bound structure, the rotamer of C114^3.36×36^ shifts substantially because of interaction with the nitrile group (Figs. 3G and 4C). In this conformation, C114^3.36×36^ engages the aromatic face of W342^6.48×48^ through SH-π interactions (Fig. 4C), whereas in the agonist-bound state, it is oriented away from W342^6.48×48^. Position 6.48, commonly referred to as the “toggle switch,” is known to play a central role in modulating TM6 rearrangement during activation in many class A GPCRs (*77*). The distinct interactions observed between C114^3.36×36^ and TM6 in the agonist-bound versus inverse agonist–bound states therefore suggest a potential role for this position in regulating D_3_R activation.

The intracellular region of the receptor undergoes substantial conformational rearrangements between agonist- and inverse agonist–bound states (fig. S18B), reflecting canonical class A GPCR activation motifs (*78*). These include P200^5.50×50^-I118^3.40×40^-F338^6.44×44^ (P^5.50×50^I^3.40×40^W/F^6.44×44^ motif), R128^3.50×50^ and E324^6.30×30^ (the ionic lock), and Y383^7.53×53^ of the NP^7.50×50^XXY^7.53^ motif, all of which adopt active or inactive conformations in the respective structures (Fig. 4D).

Comparison with previously reported inverse agonist–bound structures of D_2-like_R subtypes, including risperidone (*74*), haloperidol (*70*), spiperone (*72*), eticlopride (*33*), and nemonapride (*73*), reveals a conserved inactive receptor conformation characterized by inward positioning of TM6 (fig. S18B). Despite substantial diversity in ligand scaffolds and binding modes (Fig. 4, E and F, and fig. S18C), these ligands share two common features: (i) formation of a salt bridge with the conserved D^3.32^ residue (fig. S18C) and (ii) deeper penetration into the binding pocket (compared with agonists), stabilizing W^6.48×48^ in an inactive (upward) conformation (fig. 4G).

In EA-RK-110, this effect is achieved indirectly through nitrile-mediated interactions with C114^3.36×36^, which, in turn, modulate the position of the toggle switch. In contrast, other inverse agonists such as haloperidol (*70*), spiperone (*72*), and riperidone (*74*), directly engage W^6.48×48^ via perpendicular π interactions (fig. S18C). Because EA-RK-110 shares its overall scaffold with agonists in this study, these findings establish a direct link between a single pharmacophore element, the meta-nitrile group, and inverse agonism. Moreover, given that position 3.36 differs between D_1_R and D_2-like_R, this interaction may also contribute to subtype selectivity, with implications for SAR interpretation and drug design.

Together, our combined cryo-EM, mutational, and MD simulation analyses demonstrate that subtle shifts in OBS position can dictate ligand pharmacology, distinguishing agonism from inverse agonism and affecting efficacy. Interactions within the EBS primarily contribute to ligand stability and selectivity, with increased stability in the active-state conformation correlating with higher efficacy. These findings are consistent with previous observations that modifications to the secondary pharmacophore can indirectly reposition the orthosteric moiety and thereby tune the functional outcome of bitopic ligands (*42*).

### Design of cariprazine derivatives: Chemistry and pharmacology

Cariprazine is clinically effective in treating positive and negative symptoms of schizophrenia, across the bipolar spectrum, and as an adjunct therapy in major depression. Preclinical studies suggest that its antidepressant effects are associated with partial agonism at the D_3_R and 5-HT_1A_R (*17*, *79*, *80*). One strategy to enhance its therapeutic utility in depression is to preserve these activities while reducing engagement of the D_2_R and 5-HT_2A_R, which are primarily linked to efficacy against positive symptoms but may cause tolerability and compliance issues in depressive patients without psychotic episodes. Our structural and pharmacological analyses of cariprazine derivatives provide a framework for such design. First, we find that intrinsic efficacy of these bitopic ligands is primarily governed by the OBS. Cariprazine, ESG-2-36, and EA-RK-110 share an identical secondary pharmacophore yet differ in their arylpiperazine moieties occupying the OBS, resulting in distinct functional profiles. Comparison of cariprazine with the inverse agonist EA-RK-110 indicates that bulky substitutions on the phenyl ring should be avoided to retain partial agonism, whereas the activity of ESG-2-36 and ESG-2-37 highlights the 2-fluoro-3-methoxyphenyl motif as favorable for higher-efficacy partial agonism. Second, our data indicate that the EBS can be exploited to tune selectivity. The elongated tail of ESG-2-37 engages H29^1.32×32^, a position that varies among DR subtypes. Although this interaction does not markedly alter efficacy relative to ESG-2-36 (which shares the same OBS pharmacophore), it substantially enhances D_3_R selectivity (160-fold versus 20-fold) (*20*). Consistent with this, previous SAR studies have shown that sulfonamide containing EBS moieties can further improve D_3_R selectivity, even in the absence of the canonical carbonyl linker (*64*).

Guided by these principles, we designed cariprazine derivatives combining optimized OBS motifs, either the original 2,3-dichlorophenyl piperazine or the 2-fluoro-3-methoxyphenyl scaffold, with elongated sulfonamide-linked tails (Fig. 5A). These compounds were synthesized in a single step from ethylcyclohexyl-amine intermediates via acylation with the corresponding sulfamoyl or sulfonyl chlorides, yielding EA-Ri-146 and EA-Ri-153 (fig. S19).

**Fig. 5.**
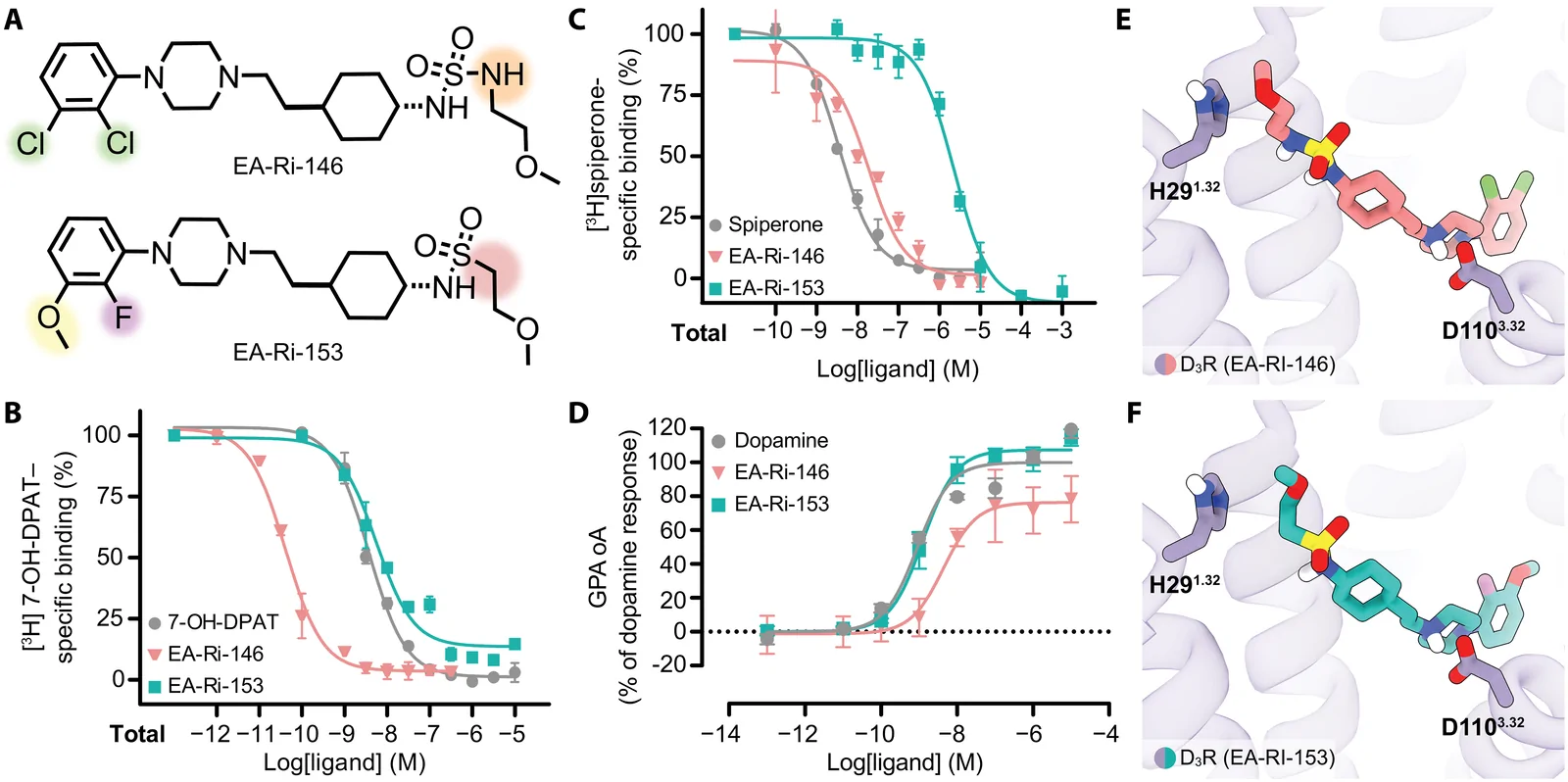
Structure and pharmacology of designed cariprazine derivatives. (**A**) Structures of the two designed compounds. (**B** and **C**) The ability of increasing concentrations of newly designed cariprazine derivatives to displace (B) [^3^H]7-OH-DPAT at the D_3_R or (C) [^3^H]spiperone at the D_2_R in competition binding assays. Data were analyzed to determine values of affinity (*K*_i_) displayed in table S8. Spiperone/7-OH-DPAT, gray circles (as indicated); EA-Ri-146, salmon triangles; EA-Ri-153, teal squares. (**D**) The ability of dopamine and newly designed cariprazine derivatives to stimulate Gα_oA_ GPA measured in a BRET assay in HEK293 cells expressing the WT D_3_R. Dopamine, gray circles; EA-Ri-146, salmon triangles; EA-Ri-153, teal squares. Data presented are the means ± SEM of three individual experiments performed in duplicate. (**E** and **F**) Binding mode of EA-Ri-146 (E) and EA-Ri-153 (F) obtained from docking the compounds to the D_3_R-ESG-2-37 complex structure solved in this study.

Both compounds display high D_3_R selectivity and potent agonist activity (table S8). The potency and efficacy of these compounds at the D_3_R were assessed in a BRET assay of Gα_oA_ G protein activation (GPA). Their affinities and those of cariprazine for the D_2_R, D_3_R, 5-HT_1A_R, and 5-HT_2A_R were determined in binding assays using [^3^H]spiperone, [^3^H]7-OH-DPAT, [^3^H]8-OH-DPAT, and [^3^H]ketanserin as tracers, respectively (table S8).

EA-Ri-146, which retains the cariprazine-like OBS pharmacophore combined with an extended EBS tail, exhibits enhanced D_3_R affinity (*K*_i_ = 0.02 nM), improved D_3_/D_2_ selectivity (490-fold), and increased potency (pEC_50_ = 8.91) with partial agonist efficacy (*E*_max_ = 76%) (Fig. 5, B to D, and table S8). Notably, it maintains moderate D_2_R affinity (*K*_i_ = 9.8 nM) within a therapeutically relevant range, while showing reduced affinity for 5-HT_1A_R (196 nM) and 5-HT_2A_R (2.5 μM), supporting its potential as a next-generation cariprazine analog.

In contrast, EA-Ri-153, which incorporates the ESG-2-37 OBS pharmacophore with a sulfonamide tail, shows comparable D_3_R affinity (*K*_i_ = 2.8 nM), high selectivity (435-fold), and potency (pEC_50_ = 8.9), as well as higher efficacy (*E*_max_ = 107%) on D_3_R. This compound has a much-decreased D_2_R affinity (*K*_i_ = 1.2 μM) (Fig. 5, B to D, and table S8). Together with its weak binding to 5-HT_1A_R (2.5 μM) and 5-HT_2A_R (22.8 μM), this profile positions EA-Ri-153 as a promising prototype for antidepressant-oriented D_3_R agonism.

We note that the use of agonist ([^3^H]7-OH-DPAT, D_3_R) and antagonist ([^3^H]spiperone, D_2_R) tracers might be expected to contribute to the degree of selectivity observed at the level of binding affinity, as they could preferentially label GPCRs and G protein–uncoupled receptors, respectively. In this regard, it is notable that the affinity of cariprazine for the D_3_R determined with this tracer is similar to that reported when [^3^H]spiperone was used (0.58 nM) (*35*). Further, as compared to cariprazine measured in the same assay, both EA-Ri-146 and EA-Ri-153 display enhanced selectivity for the D_3_R (table S8).

Assessment of blood-brain barrier (BBB) permeability using the BBB–parallel artificial membrane permeability assay (BBB-PAMPA) assay yielded logarithm of the effective permeability coefficient (logP_e_) values of −4.44 ± 0.08 and −4.53 ± 0.05 for EA-Ri-146 and EA-Ri-153, respectively, comparable to the centrally active reference compound carbamazepine (−4.46 ± 0.18) (tables S9 to S11). Predicted log(c_brain_/c_blood_) (logBB) values (0.78 and 0.76) suggest efficient brain penetration, with higher concentrations in brain tissue relative to plasma.

Docking studies using the active-state D_3_R structure indicate that both compounds adopt canonical binding poses similar to their parent scaffolds, with the elongated tails extending toward H29^1.32×32^ in the EBS (Fig. 5, E and F). These interactions likely optimize engagement of the extracellular vestibule, contributing to enhanced affinity and subtype selectivity.

Together, these results demonstrate that rational modulation of both OBS and EBS interactions enables fine-tuning of efficacy, potency, and selectivity in D_3_R-targeting ligands. While efficacy is primarily dictated by the OBS pharmacophore, EBS interactions, particularly via elongated, flexible tails, enhance ligand stability and receptor selectivity. These findings highlight the cooperative nature of bitopic ligand design and provide a foundation for developing next-generation therapeutics targeting GPCRs with tailored signaling profiles.

## MATERIALS AND METHODS

### Pharmacology

#### Cloning

cDNA in pcDNA3.1+ encoding the short isoform of the WT human dopamine D_2_ receptor (D_2S_R) or the hD_3_R incorporating an N-terminal soluble *N*-ethylmaleimide–sensitive factor attachment protein (SNAP) tag was obtained from Revvity (Bagnols-sur-Cèze, France). Enzymes for DNA cloning were sourced from New England Biolabs (Ipswich, MA). Mutant variants of the D_3_R were purchased from and generated by Genewiz using the WT D_3_R construct as a template (Leipzig, Germany).

#### Chemicals

Unless otherwise stated, reagents were purchased from Sigma-Aldrich–Merck. The selective D_2_R antagonist, spiperone, and the D_3_R agonist, 7-OH-DPAT, were obtained from Tocris Bioscience (Bristol, UK). Human embryonic kidney (HEK) 293-hD_2_ was offered by A. Bojarski (Krakow, Poland), Chinese hamster ovary (CHO)–hD_3_ cell line, the radiolabeled [^3^H]spiperone (*81*) (specific activity, 80.2 Ci/mmol), [^3^H]7-OH-DPAT (specific activity, 100 Ci/mmol), and the Ultima Gold MV harmless scintillation cocktail were acquired from PerkinElmer (Boston, USA).

### Competitive radioligand binding D_2_R, D_3_R, 5-HT_2A_R_,_ and 5-HT_1A_R

#### Preparation of cell line for binding assays

Cells were collected by a cell scraper in ice-cold phosphate-buffered saline (PBS) and centrifuged at 4°C for 15 min, at 2000*g*. The pellet was resuspended in a buffer containing 10 mM tris-HCl and 1 mM EDTA (pH 7.5) and homogenized with a Teflon-glass Braun homogenizer operating at 1500 rpm. The membrane homogenate was centrifuged at 40,000*g* for 15 min at 4°C. The final pellets were resuspended in a buffer containing 50 mM tris-HCl, 120 mM NaCl, 5 mM KCl, 5 mM MgCl_2_, and 1 mM EDTA (pH 7.5) for HEK293-hD_2_ cells and 50 mM NaHepes, 1 mM EDTA, 50 μM 8-hydroxy-quinoleine, 0.005% ascorbic acid, and 0.1% bovine serum albumin (pH 7.5) (incubation buffer) for CHO-hD_3_ cells. The membrane preparation was aliquoted and stored at −80°C until use. Protein concentration of the membrane preparations was determined by Bradford method (*82*). HEK293 cells with stable expression of human 5-HT_1A_R (prepared with the use of Lipofectamine 2000) or CHO-K1 cells with plasmid containing the sequence coding for the human serotonin 5-HT_2A_R (PerkinElmer) were maintained at 37°C in a humidified atmosphere with 5% CO_2_ and grown in Dulbecco’s modified Eagle’s medium (DMEM) containing 10% dialyzed fetal bovine serum (FBS) and G418 sulfate (500 μg/ml). For membrane preparation, cells were subcultured in 150-cm^2^ flasks, grown to 90% confluence, washed twice with PBS prewarmed to 37°C, and pelleted by centrifugation (200*g*) in PBS containing 0.1 mM EDTA and 1 mM dithiothreitol. Before membrane preparation, pellets were stored at −80°C.

#### Competitive binding experiment

In D_3_R displacement assays, aliquots of frozen HEK293-hD_2_ and CHO-hD_3_ cell line membrane homogenates (10 μg of protein) were thawed and suspended in the incubation buffer (described above). Membranes were incubated in the presence of the unlabeled ligands in increasing concentrations (10^−10^ to 10^−3^ M) at 25°C for 120 min with [^3^H]spiperone and at 25°C for 1 hour with [^3^H]7-OH-DPAT according to Seeman and Van Tol (*83*) and Lévesque *et al.* (*84*), respectively. The nonspecific and total binding was determined in the presence and absence of 10 μM unlabeled spiperone and 7-OH-DPAT, respectively. The reaction was terminated by rapid filtration under vacuum (Brandel M24R Cell Harvester) and washed three times with 5 ml of ice-cold buffer through 0.1% bovine serum albumin–coated Whatman GF/B glass fibers for D_2_R displacement and GF/C glass fibers for D_3_R displacement. The radioactivity of the dried filters was detected using UltimaGold MV aqueous scintillation cocktail with Packard Tricarb 2300TR liquid scintillation counter. The competitive binding assays were performed in duplicate and repeated at least three times.

For 5-HT_1A_R and 5-HT_2A_R competitive binding experiments, cell pellets were thawed and homogenized in 10 vol of assay buffer using an Ultra Turrax tissue homogenizer and centrifuged twice at 35,000*g* for 15 min at 4°C, with incubation for 15 min at 37°C in between. The composition of the assay buffers was as follows: for 5-HT_1A_R, 50 mM tris-HCl, 0.1 mM EDTA, 4 mM MgCl_2_, 10 μM pargyline, and 0.1% ascorbate; and for 5-HT_2A_R, 50 mM tris-HCl, 0.1 mM EDTA, 4 mM MgCl_2_, and 0.1% ascorbate. All assays were incubated in a total volume of 200 μl in 96-well microtiter plates for 1 hour at 37°C, except 5-HT_1A_R and 5-HT_2A_R that were incubated at room temperature (RT) and 27°C, respectively. The process of equilibration was terminated by rapid filtration through UNIFILTER plates with a 96-well cell harvester, and radioactivity retained on the filters was quantified on a MicroBeta plate reader (PerkinElmer, USA). For displacement studies, the assay samples contained as radioligands (PerkinElmer, USA): 2.5 nM [^3^H]-8-OH-DPAT (135.2 Ci/mmol) for 5-HT_1A_R and 1 nM [^3^H]-ketanserin (53.4 Ci/mmol) for 5-HT_2A_R. Nonspecific binding was defined with 10 μM of 5-HT in 5-HT_1A_R, whereas 20 μM mianserin was used in 5-HT_2A_R. Each compound was tested in triplicate at seven concentrations (10^−10^–10^−4^ M). The *K*_i_ values were calculated from the Cheng-Prusoff equation. Results were expressed as means of two separate experiments.

#### Data analysis

Experimental data were presented as means ± SEM. Points were fitted with the professional curve fitting program, GraphPad Prism 5.0 (GraphPad Prism Software Inc., San Diego, CA), using nonlinear regression analysis. In the competition binding assay, the “one site competition” fitting was used to establish the equilibrium binding affinity (*K*_i_ value). The median inhibitory concentration (IC_50_) values for the D_3_R according to the competition binding curves were converted into equilibrium *K*_i_ values, using the Cheng-Prusoff equation (*85*).

### TR-FRET binding studies

#### SNAP-tag labeling and membrane preparation

SNAP-tag labeling and membrane preparations were performed as previously described with minor modifications (*86*). Flp-In CHO-K1 cells were transiently transfected overnight (ON) with SNAP-D_3_R WT or mutant DNA [15 μg of DNA per T175 flask using polyethylenimine (PEI)] in 10% FBS DMEM/F-12 medium supplemented with antibiotics [penicillin (100 U/ml) and streptomycin (100 μg/ml), Gibco]. Cell culture medium was removed, cells were washed with Dulbecco’s PBS (D-PBS), and receptors were labeled by incubation in 10 ml of Tag-lite buffer (Revvity) containing 2 nmol of SNAP-Lumi4-Tb (Revvity) in a 5% CO_2_ incubator for 1 hour at 37°C. SNAP-Lumi4-Tb was then removed, and cells were washed and harvested in D-PBS using a cell scraper. Cells were collected by centrifugation at 1500 rpm for 10 min, and cell pellets were stored at −80°C before membrane preparations. Membrane preparations were performed on ice with ice-cold buffers to maintain receptor integrity. Cell pellets were resuspended in 10 ml of buffer 1 [10 mM Hepes and 10 mM EDTA (pH 7.4)]. Cells were subsequently homogenized with an IKA Works T10 Ultra-Turrax homogenizer on setting 4 for 10 short pulses. Homogenates were then made up to 20 ml with buffer 1, followed by centrifugation at 48,000*g* in an Avanti J-25 ultracentrifuge with a JA25.50 rotor (Beckman) for 30 min at 4°C. After centrifugation, the supernatant was discarded. The pellet was resuspended in 20 ml of buffer 1 and recentrifuged as before. The supernatant was then discarded, and the membrane pellet was fully resuspended in 10 mM Hepes and 0.1 mM EDTA (pH 7.4) using an IKA Labortechnik glass homogenizer (20 passes, setting 9). Final membranes were aliquoted and stored at −80°C. Membrane protein concentration was determined using the bicinchoninic acid assay (Sigma-Aldrich).

#### TR-FRET binding assays

TR-FRET binding assays were performed in white opaque 384-well low binding surface OptiPlates (PerkinElmer) at 37°C. SNAP-D_3_R cell membranes and all ligands were diluted in Hanks’ balanced salt solution (Sigma-Aldrich) supplemented with 20 mM Hepes, 0.02% (w/v) pluronic acid, 1% dimethyl sulfoxide (DMSO), and glucose (1 mg/ml) (pH 7.4). For saturation binding experiments, each well contained 2 μg of cell membrane protein in saponin (2.5 μg/ml), increasing concentrations of homologous time resolved fluorescence (HTRF) Tag-Lite D_2_R red antagonist (Revvity). Nonspecific binding for each condition was determined in the presence of 10 μM haloperidol. The microplate was incubated for 1 hour at 37°C in a total volume of 40 μl. For competition binding at each mutant, the *K*_d_ value of HTRF Tag-Lite D_2_R red antagonist at each mutant was used in the presence of increasing concentrations of unlabeled antagonists. Nonspecific binding for each condition was determined in the presence of 10 μM haloperidol. The microplate was incubated for 1 hour at 37°C in a total volume of 40 μl. Time-resolved fluorescence was detected using a PHERAstar FSX microplate reader (BMG Labtech) at 37°C. Terbium cryptate was excited at 337 nm, and emission was detected at 620 nm (donor) and 665 nm (acceptor) using the HTRF filter module with six laser flashes per well per cycle (integration start, 60 μs; integration time, 400 μs). HTRF ratios were determined by dividing the 665-nm counts (acceptor) by the 620-nm counts (donor) multiplied by 10,000. The HTRF ratios were then subtracted by the nonspecific binding HTRF ratios to give specific binding.

#### Data analysis TR-FRET binding data

For D_2_R red antagonist saturation binding data, the following equations were globally fitted to total binding (Eq. 1) and nonspecific binding (Eq. 2) data, respectively

$$Y=\frac{B_{\text{max}}\left[A\right]}{\left[A\right]+K_{A}}\text{NS}\left[A\right]$$ (1)

Y=NS[A] (2)

where *Y* is the binding, *B*_max_ is the total receptor density, [*A*] is the free D_2_R red antagonist concentration, *K*_A_ is the equilibrium dissociation constant of the D_2_R red antagonist, and NS is the fraction of nonspecific D_2_R red antagonist binding.

The concentration of ligand that inhibited half of the D_2_R red antagonist binding (IC_50_) was determined using the following equation

$$Y=\text{Bottom}+\frac{\text{Top}-\text{Bottom}}{1+10^{\left(\text{Log}[A]-{\text{Log}\text{IC}}_{50}\right)n_{H}}}$$ (3)

where *Y* denotes the percentage-specific binding, Top and Bottom denote the maximal and minimal asymptotes, respectively, IC_50_ denotes the *X* value when the response is midway between Bottom and Top, and *n*_H_ denotes the Hill slope factor. IC_50_ values obtained from the inhibition curves were converted to *K*_i_ values using the Cheng-Prusoff equation.

### BRET functional assays

#### BRET Gα_oA_ activation assay

Experiments were performed in HEK 293T cells, transiently transfected with a range of BRET sensors as described previously (*86*, *87*). Briefly, cells were cultured at 37°C in 5% CO_2_ in DMEM supplemented with 10% (v/v) FBS. Cells were seeded in 10-cm petri dishes (3 × 10^6^ cells per dish) and allowed to grow ON in full medium at 37°C in 5% CO_2_. Cells were transiently transfected the next day in medium supplemented with antibiotics [penicillin (100 U/ml) and streptomycin (100 μg/ml), Gibco] using a 1:6 total DNA-to-PEI (Polysciences Inc.) ratio. BRET constructs were as follows: 2 μg of WT-Gα_oA_, 1 μg of Gβ_1_-Venus(156 to 239), 1 μg of Gγ_2_-Venus(1 to 155), 1 μg of masGRK3ct-Rluc8, and 1 μg of receptor for GPA (*88*) assays. The following day, cells were plated in Greiner poly-d-lysine–coated, white-bottom 96-well plates [Scientific Laboratory Supplies (SLS)] in full medium. On the day of the assay (48 hours posttransfection), cells were washed once with D-PBS (Lonza, SLS) and incubated in D-PBS for 30 min at 37°C. The Rluc substrate coelenterazine h (NanoLight) was added to each well (final concentration of 5 μM), and ligands (final concentration from 10 μM to 0.01 nM in D-PBS) were added to the wells before reading the plate at 37°C in a PHERAstar FSX microplate reader (Venus and Rluc8 emission signals at 535 and 475 nm, respectively; BMG Labtech) every minute for 10 min, with an additional 30-min read at the end of the assay. For receptor depletion experiments, cells were pretreated with 0.1, 0.3, or 1 μM phenoxybenzamine (Sigma-Aldrich) diluted in D-PBS for 30 min before removal, washing with D-PBS, and equilibration as above before commencement of the assay. For the antagonist mode of the GPA assay, an EC_80_ concentration of dopamine or quinpirole was added to the wells just after compound addition to induce an 80% response that can then be inhibited by D_3_R antagonists or weak partial agonists. The ratio between Venus fluorescence and Rluc8 luminescence was used to quantify the BRET signal in each well. Data were normalized to maximal and minimal response of dopamine or quinpirole as specified on the graph axes.

#### G protein profiling EMTA BRET assays

The following BRET-based biosensor components have been previously described: human G protein subunits, p63-RlucII (*89*), Rap1GAP-RlucII (*89*), and rGFP-CAAX (*90*, *91*). The details of the transfection protocol have been previously described (*61*, *89*). On the day of the BRET experiments, the cell culture medium was removed, and the cells were washed twice with PBS and treated with Tyrode’s buffer [140 mM NaCl, 2.7 mM KCl, 1 mM CaCl_2_, 12 mM NaHCO_3_, 5.6 mM d-glucose, 0.5 mM MgCl_2_, 0.37 mM NaH_2_PO_4_, and 25 mM Hepes (pH 7.4)] and incubated at 37°C for at least 15 min. Compounds were tested in the agonist mode (in the absence of the endogenous agonist) and antagonist mode (in the presence of the endogenous agonist at its EC_80_) using the EMTA biosensor suite. For the concentration response curves of the endogenous agonist and the ligands, cells were stimulated with different concentrations of the ligands and incubated for 5 min after which the substrate Prolume Purple (1.3 μM) was added and incubated for an additional 6 min. BRET signal was measured on the Spark multimode microplate reader (Tecan) (agonist mode) (acceptor, 515 ± 20–nm filter; donor, 400 ± 70–nm filters). Cells were thereafter treated with a submaximal concentration of the endogenous agonist (EC_80_ concentration at the respective pathways) and incubated for an additional 5 min after which a second BRET read (antagonist mode) was conducted.

#### Data analysis of functional data

All data points represent the mean of at least three independent experiments performed in duplicate. Dose-response curves were fitted using the following three parameter equation

$$Y=\text{Bottom}+\frac{\text{Top}-\text{Bottom}}{1+10^{\left({\text{Log}\text{EC}}_{50}-\text{Log}[A]\right)}}$$ (4)

where Top and Bottom represent the maximal and minimal asymptote of the dose response curve, [*A*] is the molar concentration of agonist, and EC_50_ is the molar concentration of agonist required to give a response halfway between bottom and top.

To compare profiles between the WT and mutant D_3_R, agonist concentration response curves were also fitted to the operational model of agonism below

$$Y=\text{basal}+\frac{E_{m}-\text{basal}}{1+{\left(\frac{K_{A}+[A]}{\text{τ}[A]}\right)}^{n}}$$ (5)

where *E*_m_ is the maximal possible response of the system; basal is the basal level of response; *K*_A_ represents the equilibrium dissociation constant of the agonist (*A*) and τ is an index of the signaling efficacy of the agonist that is defined as *R*_T_/*K*_E_, where *R*_T_ is the total number of receptors and *K*_E_ is the coupling efficiency of each agonist-occupied receptor; and *n* is the slope of the transducer function that links occupancy to response. The analysis assumes that the transduction machinery used for a given cellular pathway are the same for all agonists, such that the *E*_m_ and transducer slope (*n*) are shared between agonists. To obtain values of *K_A_* and τ for each agonist, we treated cells with phenoxybenzamine to irreversibly block cell surface DRs as described above. This resulted in significant reductions in the maximal response. Given the proportional relationship of *R*_T_ to measured τ, *K*_A_ is invariant with receptor depletion. Hence, estimates of *K*_A_ and τ could be obtained by operational fitting of the family of concentration response curves for each agonist at WT D_2_Rs and D_3_Rs.

#### Measurement of cell surface expression of D_3_R mutants

Experiments were performed in HEK 293T cells, transiently transfected with each D_3_R WT or mutant along with the G protein constructs required for the BRET GPA assay described above. Cell-impermeable SNAP-surface Alexa Fluor 647 (A647) was diluted 1:1000 in DMEM supplemented with 10% (v/v) FBS (1 μM final concentration) and incubated at 37°C for 30 min at 5% CO_2_. Cells were washed once with DMEM with 10% FBS and once with D-PBS, and then D-PBS was added for measurement. Measurement of fluorescence (A647, 625-nm excitation and 680-nm emission; Venus, 497-nm excitation and 540-nm emission) was performed in a CLARIOstar microplate reader (BMG Labtech). The A647/Venus ratios were background corrected using ratios from unlabeled cells and fold change over the WT receptor were then plotted.

#### BBB-PAMPA experiments

The solutions of each compound were prepared in DMSO at 10 mM and then diluted with PBS (pH 7.4) buffer to obtain the donor drug solution with the final nominal concentration of 100 μM (297 μl of buffer + 3 μl of DMSO drug solution). One hundred micromolars is appropriate for direct high-performance liquid chromatography with diode-array detection (HPLC-DAD) measurements. This solution was shaken for an hour at RT in a 96-well polypropylene plate (Agilent, Waldbronn, Germany) and then filtered (Vacuum Manifold, Millipore) to avoid solid particles in HPLC measurements. Data were measured in three replicates on every single plate.

A PAMPA system was used to determine the effective permeability and membrane retention (*92*). Each well of the top plate [96-well polycarbonate-based filter donor plates (Multiscreen-IP, MAIPN4510, pore size of 0.45 μm, Millipore)] was coated with 5 μl of porcine polar brain lipid extract solution, and then 150 μl of the filtrate that contained 100 μM drug solution was put on the membrane. The bottom plate [96-well polytetrafluoroethylene acceptor plates (Multiscreen Acceptor Plate, MSSACCEPTOR, Millipore)] was filled with 300 μl of buffer solution [0.01 M PBS buffer (pH 7.4)]. The donor and acceptor plates were fit, and the donor plate was coated with a wet paper tissue and a plate lid to avoid the evaporation of the solvent. The sandwich system was incubated at 37°C or at RT for 4 or 18 hours (the plates were shaken and incubated in a Heidolph Titramax 1000). After the incubation, PAMPA sandwich plates were separated, and compound concentrations in donor [CD(*t*)] and acceptor [CA(*t*)] solutions were determined by HPLC-DAD. The concentration of the donor solution at zero time point [CD(0)] was determined using the same HPLC system after the filtration.

### Confocal microscopy

#### Cell culture

CHO DRD3 cell lines were bought from PerkinElmer. CHO DRD3 cell line was cultured in F-12 K medium (Thermo Fisher Scientific) at 37°C in 5% CO_2_. Routine mycoplasma tests were conducted monthly.

#### Competition treatment assay

Twenty-four hours before the competition treatment assay, cells were seeded in eight-well chambers (ibidi GmbH, Germany) at a density of 20,000 cells per well with the corresponding culture medium. The glass slide surface was coated with Fibronectin, collagen and albumin (FNC) coating medium (Athena Environmental Sciences Inc., MD, USA). Treatment with FluoCar and FluoCar2 [concentration of 10 nM)] was carried out after a medium change for 1 hour at 37°C in 5% CO_2_. Following a two times wash with 1× PBS, Car (competitor drug treatment, 100 nM) was applied for 2 hours at 37°C in 5% CO_2_. Posttreatment, supernatant removal was followed by two washes with 1× PBS before fixation. Fixation was performed at RT using 4% paraformaldehyde (Thermo Fisher Scientific) for 15 min. Subsequent to two additional washes with 1× PBS, Fluoro-Gel with 4′,6-diamidino-2-phenylindole mounting medium (ProSciTech) was applied before covering the slide. The samples were investigated with a Zeiss LSM 710 confocal microscope (Zeiss, Jena, Germany).

#### Chemical syntheses

All starting materials were purchased from commercial sources, or the synthesis was published earlier (*20*, *64*). The compound EA-Ri-112, EA-RK-110, and cariprazine were synthesized on the basis of our earlier publication (*42*). The HCl salts of ESG-2-36 and ESG-2-37 [compounds 6c and 13c, respectively, in Gogarnoiu *et al.* (*20*)] were prepared in the Newman Lab as previously described (*20*). For the experiments with the FluoCar, we used the same batch that we published earlier (*9*). HPLC chromatograms of all synthesized compounds are published for purity verification. All synthesized new compounds were identified with HPLC–mass spectrometry, high-resolution mass spectrometry, and nuclear magnetic resonance measurements. The details of the synthesis of the previously undiscovered compounds are described in data S2 and in (*93*).

### Structural studies

#### Construct design

For structural studies of the active complex, hD_3_R (DRD3, UniPort ID X5D2G4) was cloned into pFastBac1 (Thermo Fisher Scientific) following an N-terminal haemagglutinin membrane–targeting signal peptide, a FLAG-tag, and a tobacco etch virus nuclear inclusion A endopeptidase protease cleavage site. The C terminus was attached to a LgBit subunit, via a 15–amino acid linker, to stabilize the interaction of the receptor with Gβ (*53*, *54*). Gβ was N-terminally tagged with 6×His, followed by a human rhinovirus (HRV) 3C protease cleavage site, and contained a HiBit subunit at the C terminus. Gγ was linked to mini-Gα_o_ iN18 (in which the first 18 amino acids were mutated to the corresponding sequence from Gαi to serve as antigen for ScFv16 (*55*). Both Gβ and Gγ-mini-Gαo iN18 were cloned into pFastBac Dual (Thermo Fisher Scientific). For structural studies of the inactive receptor, residues A215-V336 (ICL3) of D_3_R were replaced with a BRIL sequence, and the LgBit subunit was removed. Baculoviruses were generated using the Bac-to-Bac system (Thermo Fisher Scientific) and prepared according to the manufacturer’s instructions in *Spodoptera frugiperda* 9 (Sf9) cells, grown in ESF 921 medium (Expression Systems).

#### Protein expression and purification

For the active complex, D_3_R-LgBit and Gβ-HiBit plus Gγ-mini-Gα_o_ were coexpressed in Sf9 cells at a ratio of 1:1. Cells were cultured in ESF 921 at 27°C and harvested by centrifugation after 60 hours. Cells were lysed in lysis buffer {20 mM Hepes (pH 7.5), 100 mM NaCl, 15% glycerol, 20 mM KCl, 10 mM MgCl_2_, 100 μM Tris(2-carboxyethyl)phosphine hydrochloride (TCEP), and protein inhibitor cocktail [PIC; benzamidine (160 μg/ml), 1 mM phenylmethylsulfonyl fluoride (PMSF), and leupeptin (2.5 μg/ml)]} at RT in the presence of Apyrase (50 mU/ml; Sigma-Aldrich) and 20 to 40 μM agonist to instigate complex formation. Membranes were harvested by centrifugation (37,000*g* for 30 min at 4°C) and then solubilized with 0.5% (w/v) lauryl maltose neopentylglycol (LMNG; Anatrace) and 0.1% (w/v) cholesterol hemisuccinate (CHS; Anatrace) in solubilization buffer [20 mM Hepes (pH 7.5), 150 mM NaCl, 15% glycerol, 20 mM KCl, 10 mM MgCl_2_, PIC, benzonase (7.5 μl/liter), and 100 μM TCEP] in the presence of 20 to 40 μM agonist at 4°C for 1.5 hours. Insoluble material was removed by centrifugation (37,000*g* for 30 min at 4°C), and supernatant was loaded onto α-FLAG resin (anti-DYKDDDDK G1 affinity resin, GenScript), using a gravity column. Resin was washed with 20 column volumes (CVs) of FLAG wash buffer [20 mM Hepes (pH 7.5), 150 mM NaCl, 10 mM MgCl_2_, 100 μM TCEP, 10 μM agonist, 0.075% (w/v) LMNG, 0.025% (w/v) glyco-diosgenin (GDN), and 0.01% (w/v) CHS]. Protein complex was eluted with FLAG peptide (0.25 mg/ml; DYKDDDDK peptide, GenScript) in FLAG wash buffer. ScFv16 was added to the eluted complex at a molar ratio of 1:1.5 and incubated ON at 4°C. Sample was concentrated using Amicon ultra centrifugal filter [molecular weight cutoff (MWCO), 100 kDa; Merck Millipore] and loaded onto Superose 6 Increase 10/300 GL (GE Healthcare), using size exclusion chromatography buffer [20 mM Hepes (pH 7.5), 150 mM NaCl, 10 mM MgCl_2_, 10 μM agonist, 100 μM TCEP, 0.00075% (w/v) LMNG, 0.00025% (w/v) GDN, and 0.0001% (w/v) CHS]. Fractions corresponding to the complex were concentrated to >10 mg/ml and used for cryo-EM experiments and SDS–polyacrylamide gel electrophoresis (PAGE) (fig. S2).

For the inactive receptor, the D_3_R-BRIL construct was expressed in Sf9 cells. Cells were treated as described above and then lysed in lysis buffer at RT. Membranes were harvested by centrifugation (37,000*g* for 30 min at 4°C) and then solubilized with 0.5% (w/v) LMNG and 0.1% (w/v) CHS in the solubilization buffer. Protein was then purified using α-FLAG resin, followed by size exclusion chromatography using Superose 6 Increase 10/300 GL, as described above. Fractions corresponding to the receptor were concentrated, anti-BRIL Fab (BAG2) was added at a molar ratio of 1:1.5 and incubated for 1 hour at RT. Sample was then concentrated to ∼12 mg/ml for cryo-EM studies and SDS-PAGE (fig. S2).

#### ScFv16 purification

ScFv16 was purified as previously described (*55*). Briefly, ScFv16, tagged at the C terminus with 8His, was expressed in secreted form in *Trichoplusia ni* Hi5 cells (Tni, Expression Systems LLC) for 48 hours at 27°C. Cells were discarded by centrifugation; the pH of the supernatant was adjusted to 8.2 using tris buffer; and 1 mM NiCl_2_, 5 mM CaCl_2_, and PIC were added. Solution was stirred for 1 hour at RT, followed by centrifugation at 4000*g* for 15 min and filtration through a 0.45-μm filter. Supernatant was then loaded onto HisTrap HP His tag protein purification columns (Cytiva), washed with 20 mM Hepes (pH 7.5), 500 mM NaCl, and 20 mM imidazole, and eluted with 20 mM Hepes (pH 7.5), 100 mM NaCl, and 250 mM imidazole. Protein was then loaded onto Superdex 75 10/300 GL (GE Healthcare) using 20 mM Hepes (pH 7.4) and 100 mM NaCl. Appropriate fractions were concentrated, flash-frozen in liquid nitrogen in the presence of 20% glycerol, and stored in −80°C.

#### Anti-BRIL Fab (BAG2) expression and purification

*Escherichia coli* C43 cells were used for the expression of BAG2 (*94*). ON starter cultures were inoculated with a single colony into 2x Yeast Extract Tryptone (2×YT) medium, supplemented with ampicillin (100 μg/ml), and incubated at 37°C while shaking at 220 rpm. Starters were then diluted 1:100 into TB autoinduction medium [terrific broth supplemented with 0.4% glycerol, 0.01% glucose, 0.02% lactose, 1.25 mM MgSO_4_ and ampicillin (100 μg/ml)] and grown at 37°C for 6 hours, while shaking at 225 rpm, followed by ON incubation at 30°C, while shaking at 225 p.m. Cells were then harvested by centrifugation at 6000*g* for 10 min, flash frozen in liquid nitrogen, and stored at −80°C. For purification, cells were resuspended in lysis buffer [20 mM sodium phosphate (pH 7.5), 200 mM NaCl, deoxyribonuclease I (1 μg/ml), and 1 mM PMSF] and sonicated twice for 8 min (30% amplitude, 15-s on, and 15-s off). The lysate was then incubated at 65°C for 30 min, followed by centrifugation at 25,000*g* for 30 min at 4°C. The supernatant was filtered using a 0.22-μm filter and loaded onto an equilibrated protein L column (Cytiva HiTrap protein L antibody fragment purification 5-ml columns) at a rate of 5 ml/min. The column was then washed with 10 CVs of running buffer [20 mM Hepes (pH 7.5) and 500 mM NaCl], at a rate of 2.5 ml/min, and protein was eluted with 0.1 M acetic acid into wells containing 1 M tris (pH 8) (buffer:eluent ratio of 1:5) at a rate of 2.5 ml/min. BAG2 was then concentrated using 10-kDa MWCO and dialyzed against 20 mM Hepes (pH 7.5) and 150 mM NaCl. Protein was then aliquoted, flash frozen, and stored in −80°C in the presence of 20% glycerol.

#### Cryo-EM, model building, and refinement

Grids for cryo-EM were prepared by placing a 3.5-μl sample on glow-discharged UltrAUFoil (R1.2/1.3, 300 mesh, 2 min, 15 mA; PELCO easiGlow, Ted Pella). Grids were blotted for 2.5 to 3.5 s at 22°C and 100% humidity, using Vitrobot Mark IV (FEI), followed by plunge-freezing in liquid ethane. Movies were recorded on Titan Krios electron microscope (Thermo Fisher Scientific) equipped with a K3 direct electron detector (Gatan), operating at 300 kV, at a calibrated fold magnification of ×105,000, corresponding to a magnified pixel size of 0.8423 Å. BioQuantum energy filter (Gatan) was operated with an energy slit width of 20 eV. Micrographs were recorded at an exposure rate of ∼26 e^-^/Å^2^ per second and defocus values ranging from −0.8 to −2.0 μm. Total exposure time was 1.49 s, with intermediate frames being recorded every 0.03 s, resulting in an accumulated dose of ∼39 ē/ Å^2^ and a total of 45 frames per micrograph. Data acquisition was done automatically using EPU (Thermo Fisher Scientific, FEI). Specific recording data for each dataset can be found in table S1.

cryoSPARC (v4.2.1) (*95*) was used for patch motion correction, contour transfer function estimation, particle picking, 2D classification, ab initio model reconstruction, and heterogeneous refinement, followed by nonuniform refinement for homogeneous groups with well-resolved features. To refine the homogeneity of particles of the active complexes, particle coordinates with their assigned angles were imported into Relion 3.1 (*96*) and subjected to alignment-free 3D classification. Particles corresponding to classes with well-defined density were transferred back to cryoSPARC, subjected to nonuniform refinement, followed by local refinement on the receptor and the G proteins portions for the EA-Ri-112– and ESG-2-37–bound complexes. Details regarding each dataset, including resolution values based on gold-standard Fourier shell correlation using the 0.143 criterion (fig. S3A), can be found in table S1. A flowchart describing the data processing step for D_3_R–G protein–ESG-2-37 complex can be found in fig. S2G. Local resolution was calculated using Phenix Local_resolution (*97*) and plotted using ChimeraX (*98*). Angular distribution for particle projections was plotted using cryoSPARC (v4.2.1) (*95*) (fig. S3, B and C).

Initial coordinates for D_3_R were generated using Protein Data Bank (PDB) 7CMV (*51*). G protein coordinates were generated using PDB 8E9X (*99*). Ligand coordinates and geometry restraints were generated using Phenix eLBOW (*100*). The models were initially docked into the EM maps using ChimeraX, followed by iterative manual building in Coot (*101*). Ligand positions were refined by GlideEM following the GemSpot pipeline (*102*). Final models were subjected to global refinement and minimization in real space using Phenix real-space refinement (*97*). Model geometry was evaluated using MolProbity (*103*). Final refinement parameters can be found in table S1. Structures have been deposited into the PDB and Electron Microscopy Data Bank (EMDB) databases with accession codes 9T8C or 32FD/EMD-55684 (D_3_R-cariprazine), 9T7Q/EMD-55651 (D_3_R-EA-Ri-112), 9T7P/EMD-55650 (D_3_R-ESG-2-36), 9T88/EMD-55683 (D_3_R-ESG-2-37), and 9T8D/EMD-55685 (D_3_R-EA-RK-110).

### Molecular dynamics simulations

#### Protein preparation and system building

The refined cryo-EM structures were used as starting structures for the molecular dynamics simulations of the four agonist-bound structures (cariprazine, ESG-2-37, ESG-2-36, and EA-Ri-112) and the antagonist-bound structure (EA-RK-110). All stabilizing subunits added to the WT sequence to enhance the stability of the complexes were removed before model preparation. To generate a consensus sequence for the receptors, the TM1 helix was expanded to include the residues from P28 using the ESG-2-37 bound D_3_ structure as template for the agonists displaying more flexibility in that region therefore missing the top part of TM1 in the density. Following GPCRmd protocol, as the ICL3 loop in the D_3_R is too long to be remodeled completely, a chain break was introduced in the middle, and the helixes were expanded with five residues until S233 on TM5 and G313 on TM6. All modeling work was carried out with the Schrödinger Program Package (*104*). Unphysical N and C termini were capped with acetyl group and *N*-methylamide, respectively. The missing side chains were added, the protonation states and tautomer forms were assigned using Propka at pH 7, the hydrogen bonds were optimized, and the steric clashes were eliminated with restrained minimization with the Protein Preparation Wizard of Schrödinger (*104*). D75^2.50^ was protonated in the active structures and was kept deprotonated in the inactive structure similarly as described in the GPCRmd protocol and previous simulations (*105*). Disulfide bonds were formed between Cys103-Cys181 and Cys355-Cys358. CHARMM-GUI (*106*) input preparator was applied to build the membrane embedded system for the simulations. The protein was aligned with the Orientations of Proteins in Membranes PPM 2.0 webserver (*107*) and embedded in POPC (1-palmitoyl-2-oleoyl-sn-glycero-3-phosphocholine) bilayer and solvated with TIP3P (Transferable Intermolecular Potential 3 Points) water applying 22.5-Å water thickness resulting in a 150 Å–by–150 Å–by–162 Å tetragonal periodic box for the agonists bound G protein–coupled D_3_R structures and 150 Å–by–150Å–by–124 Å box for the antagonist bound D_3_R structure. The systems were neutralized and the ion concentration was set to 0.15 M with NaCl. Amberff14SB (*108*) force field was applied for the protein and lipid molecules, while for the ligand Generalized Amber Force Field (GAFF) (*109*), parameters were applied with restrained electrostatic potential (RESP) (*110*) charges calculated with Gaussian16 (*111*).

#### Simulation protocol

All simulations were carried out using the Amber22 (*112*) program package. The CHARMM-GUI default equilibration protocol was followed to minimize and equilibrate the system in seven steps. Briefly, first, the system was energy minimized in 5000 steps using the steepest descent algorithm for the first 2500 steps and the conjugate gradient method for the remaining steps with restraints on the protein, ligand, and membrane. The restrains are gradually lifted during the equilibration that contains six steps, starting with two 125-ps-long simulations in canonical (NVT) ensemble with 1-fs time steps, followed by one 125-ps-long isothermal-isobaric ensemble (NPT) step with 1-fs time step and four 500-ps-long NPT steps with 2-fs time steps. The three, independent parallel production simulations were run for 1 μs each. The temperature was set to 310 K in the simulations and was controlled by Langevin dynamics (*113*) with a friction coefficient of 1 ps^−1^. The target pressure set to 1 bar was controlled with Berendsen barostat with a relaxation time of 1 ps. A surface tension (0 dynes/cm) was applied resulting in a semi-isotropic scaling with the number of interfaces set to two due to the bilayer. A 12-Å cutoff was applied for the nonbonded interactions. Bonds containing hydrogens are constrained with SHAKE algorithm (*114*) for the solutes and SETTLE algorithm (*115*) for the rigid water molecules. The trajectories were analyzed with cpptraj available in Amber22 and getContacts Python package (https://getcontacts.github.io/). For clustering, the receptor backbones were aligned and complete linkage clusterings were performed with a 3.0-Å cutoff.

#### Docking

All calculations were performed using the Schrödinger Small-Molecule Drug Discovery Suite 2024-4, Schrödinger LLC, New York, NY (2024). The 3D structure of the active hD_2_R was downloaded from GPCRdb [PDB 7JVR (*58*)]. The D_3_R-ESG-2-37 complex structure solved in this study was used for the D_3_R calculations. The structures were prepared using the Schrödinger Protein Preparation Wizard with default settings with the restrained minimization step included, and all waters were removed. Ligands were protonated at the arylpiperazine basic amine moiety. Subsequently, all ligands were prepared for docking using Schrödinger LigPrep with the OPLS4 force field (*116*). Schrödinger Glide Single Precision was used for docking the ligands. In the experimental structures, the grid was placed on the centroid of the cocrystallized ligands. The inner box size was set to 12 Å by 12 Å by 12 Å, and the outer box size was set to the diameter of the ligands. A H-bond constraint was defined with the characteristic D3.32 residue of aminergic receptors. In all these cases, 15 poses were included in postdocking minimization, and 5 poses were saved, which were ranked by the docking score separately for each ligand. Binding modes were analyzed in Schrödinger Maestro. The best scoring binding modes are shown in the figures.

## Supplementary Materials

### This PDF file includes:

Figs. S1 to S19 Tables S1 to S11 Supplementary Text References
