# Supplementary material for: The structure of the dopamine D3 receptor bound to cariprazine reveals principles for partial agonists with designed pharmacology
Source: Sci Adv. 2026 Aug 28;12(35):eaef7411. doi: 10.1126/sciadv.aef7411 (PMC13524052; doi:10.1126/sciadv.aef7411)
Supplement: Supplementary file 1 [file sciadv.aef7411_sm.pdf]

Science Advances
AAAS

Supplementary Materials for

The structure of the dopamine D_3 receptor bound to cariprazine reveals principles for partial agonists with designed pharmacology

Eliane Hadas Yardeni *et al.*

Corresponding author: J. Robert Lane, rob.lane@nottingham.ac.uk; György M. Keserű, keseru.gyorgy@ttk.hu; Moran Shalev-Benami, moransb@weizmann.ac.il

Sci. Adv. **12**, eaef7411 (2026)
DOI: 10.1126/sciadv.aef7411

This PDF file includes:

Figs. S1 to S19
Tables S1 to S11
Supplementary Text
References

```
                                                      1.29x29                                       1.60x60  2.37x37
D1R    MRTLNT--SAMDG---------------TGLVVERDFSVRILTACF-LSLLILSTLLGNTLVCAAVIRFRHLRSKVTNFF
D2R    MDPLNL--SWYDDDLERQNW----SRPFNGSDGKADRPHYNYYATL-LTLLIAVIVFGNVLVCMAVSREKALQT-TTNYL
D3R    MASLSQ--LSG----HLNYT----CGAENS-TGASQARPHAYYALS-YCALILAIVFGNGLVCMAVLKERALQT-TTNYL
D4R    MGNRST--ADADGL-LAGRG----PAAGASAGASAGLAGQGAAALVGGVLLIGAVLAGNSLVCVSVATERALQT-PTNSF
D5R    MLPPGSNGTAYPGQFALYQQLAQGNAVGGSAGAPPLGPSQVVTACL-LTLLIIWTLLGNVLVCAAIVRSRHLRANMTNVF
       *   .                        .             *      **   : ** *** ::   : *::  ** :

       2.68x67  3.21x21  3.32x32  3.33x33  3.36x36  3.56x56  4.38x38
D1R    VISLAVSDLLVAVLVMPWKAVAEIAG-FWPFGS-FCNIWVAFDIMCSTASILNLCVISVDRYWAISSPFRYER--K-MTP
D2R    IVSLAVADLLVATLVMPWVVYLEVVG-EWKFSRIHCDIFVTLDVMMCTASILNLCAISIDRYTAVAMPMLYNT--RYSSK
D3R    VVSLAVADLLVATLVMPWVVYLEVTGGVWNFSRICCDVFVTLDVMMCTASILNLCAISIDRYTAVVMPVHYQHGTGQSSC
D4R    IVSLAAADLLLALLVLPLFVYSEVQGGAWLLSPRLCDALMAMDVMLCTASIFNLCAISVDRFVAVAVPLRYNR--Q-GGS
D5R    IVSLAVSDLFVALLVMPWKAVAEVAG-YWPFGA-FCDVWVAFDVMCSTASILNLCVISVDRYWAISRPFRYKR--K-MTQ
       ::***.:**::* **:*  .  *: *  * :.   *:  :::*:* .****:***.**:**: *:  *. *:

       4.63x64  5.35x36  5.42x43  5.46x461
D1R    KAAFILISVAWTLSVLISFIPVQLSWHKAKPTSPSDGNA--------------TSLAETIDNCDSSLSRTYAISSSVISF
D2R    RRVTVMISIVWVLSFTIS-CPLLFGLNNA-----------------------------DQNECI-IANPAFVVYSSIVSF
D3R    RRVALMITAVWVLAFAVS-CPLLFGFNTT----------------------------GDPTVCS-ISNPDFVIYSSVVSF
D4R    RRQLLLIGATWLLSAAVA-APVLCGLNDVRG--------------------------RDPAVCR-LEDRDYVVYSSVCSF
D5R    RMALVMVGLAWTLSILISFIPVQLNWHRDQAASWGGLDLPNNLANWTPWEEDFWEPDVNAENCDSSLNRTYAISSSLISF
       :   :::  .* *:  ::  *:  . :                                   *    .  :.: **: **

       5.76x76
D1R    YIPVAIMIVTYTRIYRIAQKQ-IRRIAAL-ERAAVH--------------------------------------------
D2R    YVPFIVTLLVYIKIYIVLRRR-RKRVNTK-RSSRA---------------------------------------------
D3R    YLPFGVTVLVYARIYVVLKQRRRKRILTR-QNSQC---------------------------------------------
D4R    FLPCPLMLLLYWATFRGLQRWEVARRAKLHGRAPRRPSGPGPPSPTPPAPRLPQDPCGPDCAPPAPGLPRGPCGPDCAPA
D5R    YIPVAIMIVTYTRIYRIAQVQ-IRRISSL-ERAAEH--------------------------------------------
       ::*  : ::  *   :   :     *       :

       5.86x86
D1R    ----------------------------------AKNCQTT---------------------------------------
D2R    ---------------------------------FRAHLRAPLKGNCTHPEDMKLCTVIMKSNGSFPVNRRRVEAARRAQE
D3R    ---------------------------------NSVRPGFPQQTLSPDPAHLELKRYY----------------------
D4R    APSLPQDPCGPDCAPPAPGLPPDPCGSNCAPPDA-----------------VRAAAL-----------------------
D5R    ----------------------------------AQSCRSS---------------------------------------

D1R    --------------------------------------------------------------------TGNGKPVECSQP
D2R    LEMEMLSSTSPPERTRYSPIPPSHHQLTLPDPSHHGLHSTPDSPAKPEKNGHA-KDHPKIAKIFEIQTMPNGKTRTSLKT
D3R    ---------------------SICQDTALGGPGFQERG---GELKREEKTRNSLSPTIAPKLSLEVRKLSNGRLSTSLKL
D4R    ------------------------------------------------------------------------PPQTPPQT
D5R    ---------------------------------------------------------------------------AACAP

       6.24x24  6.48.48  6.51x51  6.52x52  6.55x55  6.61x61  7.31x30
D1R    -ESSFKMSFKRETKVLKTLSVIMGVFVCCWLPFFILNCILPFCGSGETQ----PFCIDSNTFDVFVWFGWANSSLNPIIY
D2R    -MSRRKLSQQKEKKATQMLAIVLGVFIICWLPFFITHILNIHCD-CN---------IPPVLYSAFTWLGYVNSAVNPIIY
D3R    GPLQPRGVPLREKKATQMVAIVLGAFIVCWLPFFLTHVLNTHCQTCH---------VSPELYSATTWLGYVNSALNPVIY
D4R    RRRRRAKITGRERKAMRVLPVVVGAFLLCWTPFFVVHITQALCPACS---------VPPRLVSAVTWLGYVNSALNPVIY
D5R    -DTSLRASIKKETKVLKTLSVIMGVFVCCWLPFFILNCMVPFCSGHPEGPPAGFPCVSETTFDVFVWFGWANSSLNPVIY
                 :* *. : :.:::*.*: ** ***: :     *             :     .. .*:*:.**::**:**

       7.56x56  8.47x47  8.59x59
D1R    -AFNADFRKAFSTLLG-C terminus sequence
D2R    TTFNIEFRKAFLKILH-C terminus sequence
D3R    TTFNIEFRKAFLKILS-C terminus sequence
D4R    TVFNAEFRNVFRKALR-C terminus sequence
D5R    -AFNADFQKVFAQLLG-C terminus sequence
        .** :*::.*   *     *
```

Fig. S1. Sequence alignment of human dopamine receptors. Helices are marked with a grey background. Residues are numbered according to D$_3$R, where both the Ballesteros-Weinstein nomenclature and the GPCRdb structure-based position are noted. Conserved residues within the OBS are marked in red. Highly conserved residues are indicated by (*), conserved residues are indicated by (:), similar residues are indicated by (.). Alignment was done using T-COFFEE (*117*).

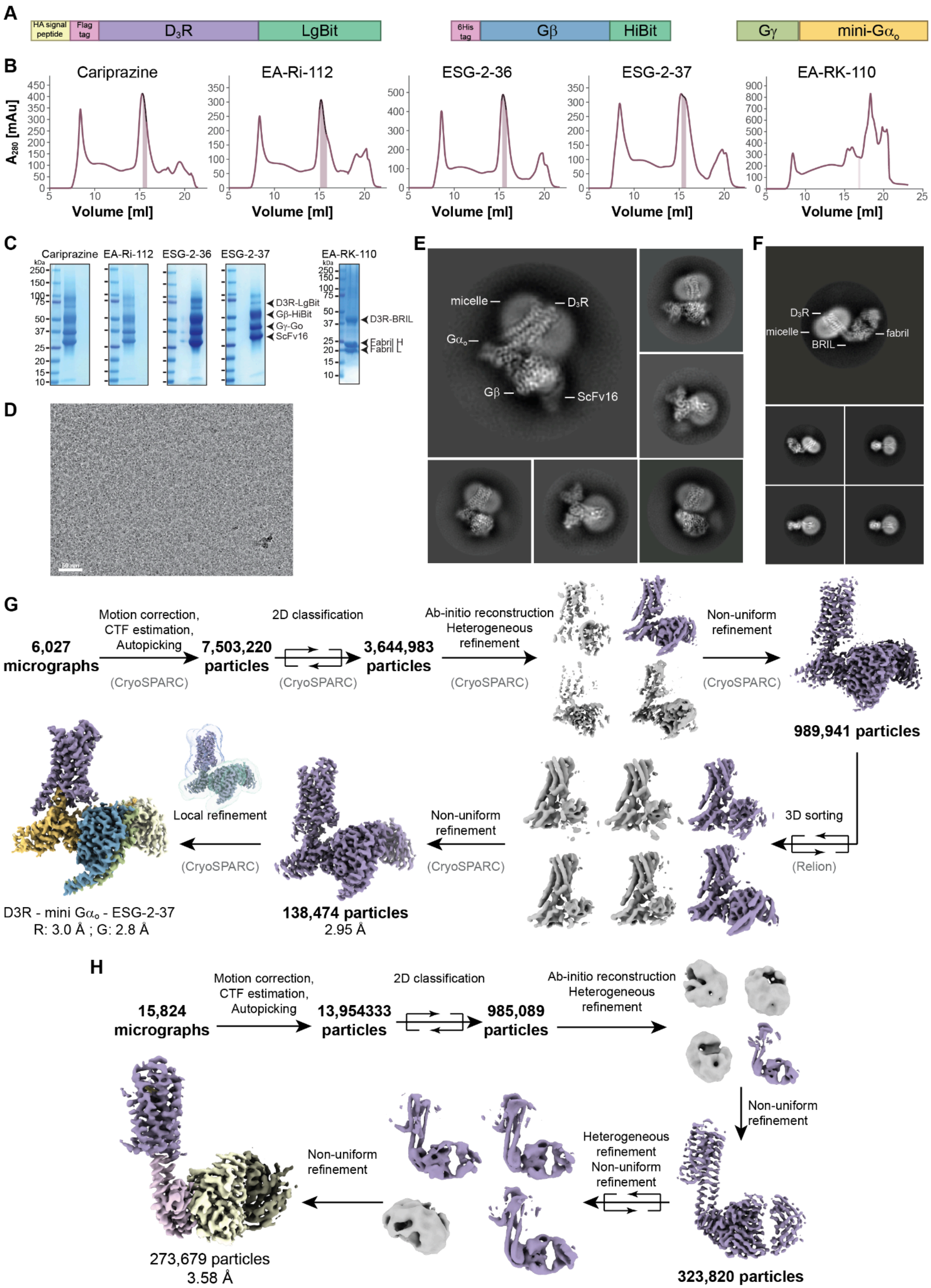
A
HA signal peptide
Flag tag
D3R
LgBit
6His tag
Gβ
HiBit
Gγ
mini-Gαo
B
Cariprazine
EA-Ri-112
ESG-2-36
ESG-2-37
EA-RK-110
A280 [mAu]
Volume [ml]
C
Cariprazine
EA-Ri-112
ESG-2-36
ESG-2-37
EA-RK-110
kDa
D3R-LgBit
Gβ-HiBit
Gγ-Go
ScFv16
D3R-BRIL
Fabril H
Fabril L
D
E
micelle
D3R
Gαo
Gβ
ScFv16
F
D3R
micelle
BRIL
fabril
G
6,027 micrographs
Motion correction, CTF estimation, Autopicking
(CryoSPARC)
7,503,220 particles
2D classification
(CryoSPARC)
3,644,983 particles
Ab-initio reconstruction Heterogeneous refinement
(CryoSPARC)
Non-uniform refinement
(CryoSPARC)
989,941 particles
3D sorting
(Relion)
Non-uniform refinement
(CryoSPARC)
138,474 particles
2.95 Å
Local refinement
(CryoSPARC)
D3R - mini Gαo - ESG-2-37
R: 3.0 Å ; G: 2.8 Å
H
15,824 micrographs
Motion correction, CTF estimation, Autopicking
13,954333 particles
2D classification
985,089 particles
Ab-initio reconstruction Heterogeneous refinement
Non-uniform refinement
323,820 particles
Heterogeneous refinement Non-uniform refinement
Non-uniform refinement
273,679 particles
3.58 Å

Fig. S2. Purification of D_3R-G protein complexes and the inactive D_3R complex and their cryo-EM processing. (**A**) Schematic representation of the constructs used for D_3R-G protein complex purification. (**B**) Size-exclusion chromatograms of the complexes, in the presence of the different ligands, as indicated. Fractions used for cryo-EM are shown in purple. (**C**) Coomassie stained SDS-PAGE of purified D_3R complexes. (**D**) A representative cryo-EM micrograph from the ESG-2-37 complex sample. (**E**) Representative reference-free 2D class averages from the ESG-2-37 complex sample. (**F**) Representative reference-free 2D class averages from the EA-RK-110 complex sample. (**G**) Processing flowchart of the ESG-2-37 complex sample, including particle selection, 2D and 3D classifications, particle sorting, masking, and final map reconstruction. Only samples purified in the presence of EA-Ri-112 and ESG-2-37 underwent local refinement. cryoSPARC v4.2.1 and RELION 3.1 were used for data processing, as indicated in parenthesis within the diagram. Other data sets were processed similarly, and specific details are indicated in **table S1**. (**H**) Processing flowchart of the EA-RK-110 complex sample, including particle selection, 2D and 3D classifications, particle sorting, masking, and final map reconstruction. cryoSPARC v4.2.1 was used for data processing. Specific details are indicated in **table S1**.

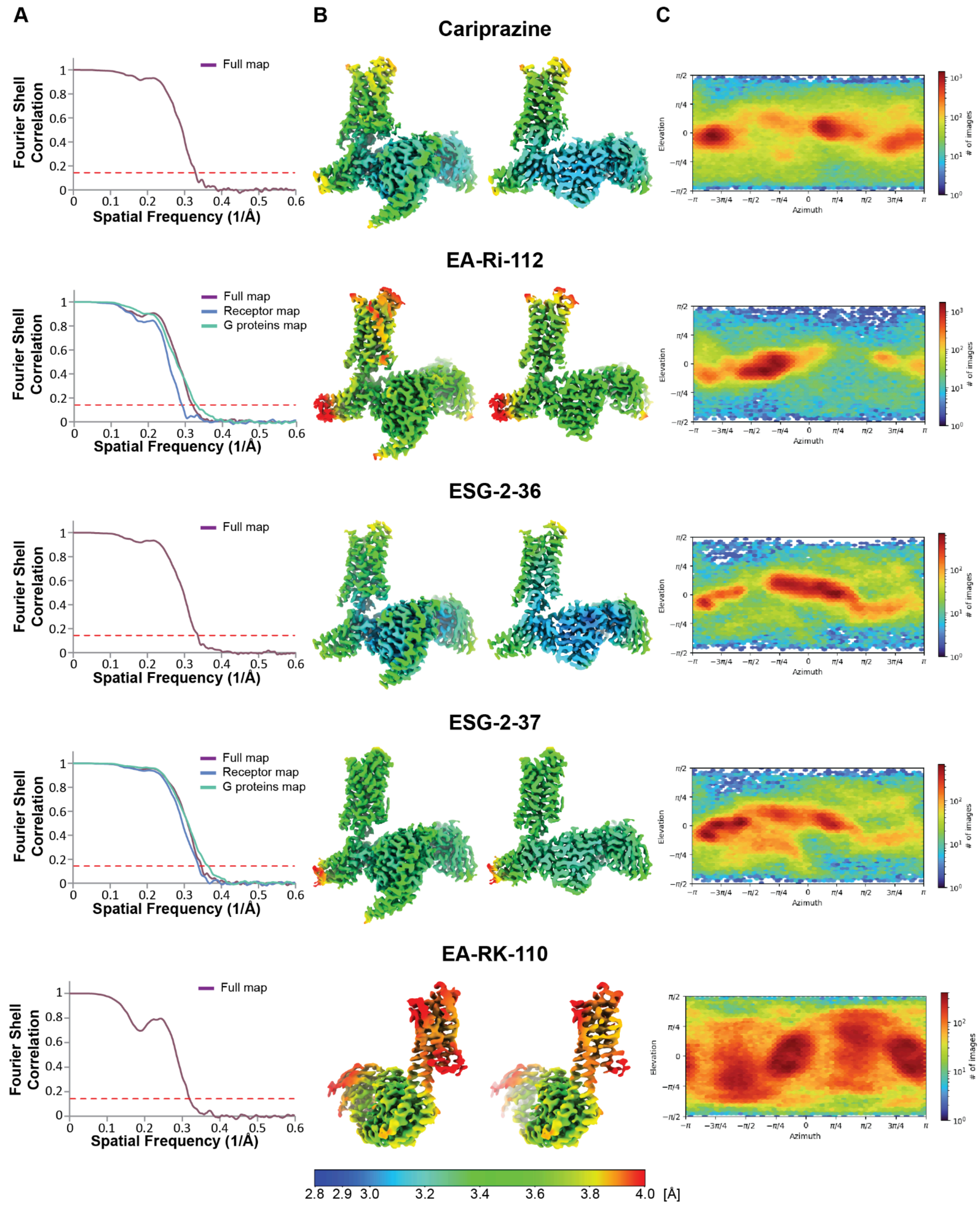

Fig. S3. D_3R complex maps and model qualities. (**A**) Gold standard Fourier shell correlation (FSC) curves of half maps that were individually calculated, indicating an average resolution at 0.143 FSC. The global map resolution is shown in purple. For EA-Ri-112 and ESG-2-37, the resolution for each local-refined map is shown in blue and teal, respectively. (**B**) Final cryo-EM maps colored by local resolution. Resolution distribution was calculated using Phenix Local_resolution. (**C**) Angular distribution for particle projections. Heat maps, showing the number of particles for each viewing angle, were plotted using cryoSPARC v4.2.1.

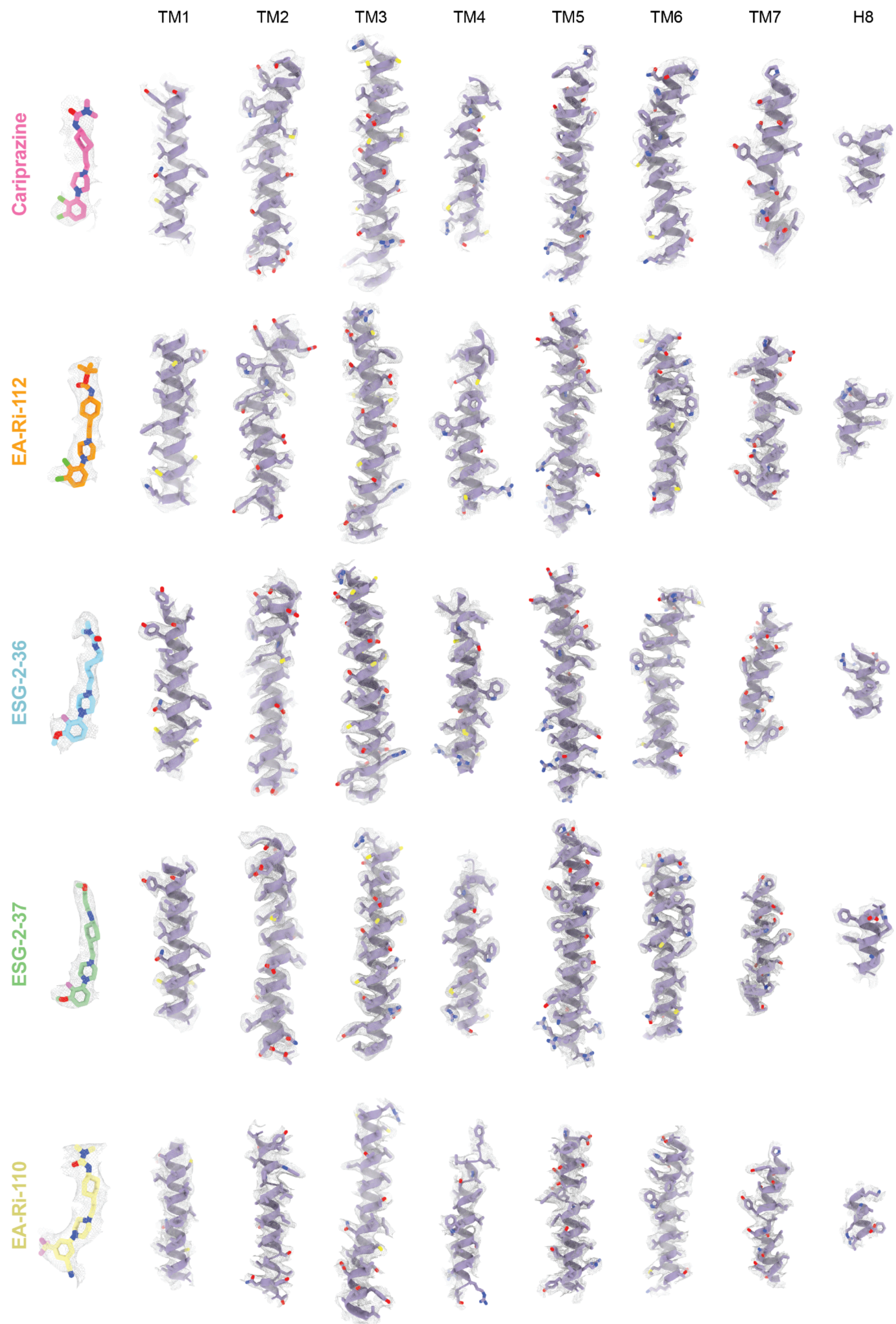

Fig. S4. D_3R helices and ligands in their cryo-EM density map. Contour levels: Cariprazine - 0.120; EA-Ri-112 - 0.109; ESG-2-36 - 0.122; ESG-2-37 - 0.127; EA-RK-110 - 0.120.

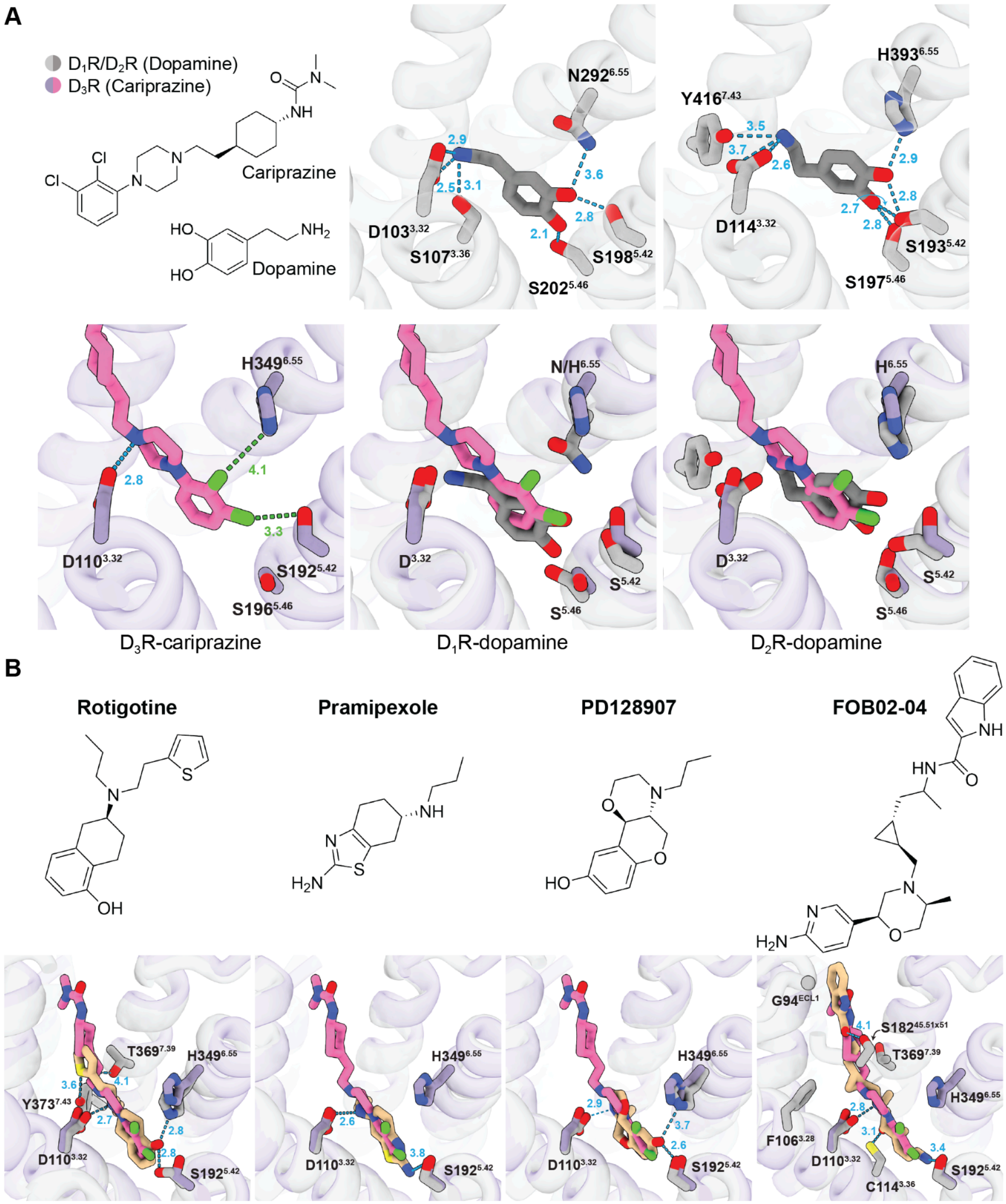

Fig. S5. Binding positions of D_3R agonists. (**A**) Comparison between the binding of dopamine in D_1R (PDB 7CKZ (*76*), grey) and in D_2R (PDB 8U02 (*48*), grey) to the binding of cariprazine (pink) in D_3R. (**B**) Ligand structures (upper panels) and their position within the binding pocket of D_3R (bottom panels, khaki) in comparison to cariprazine (pink). In all cases, the ligands bind in the OBS. Only in the case of cariprazine and FOB02-04 the binding site extends to the EBS. Lengths are shown in Å.

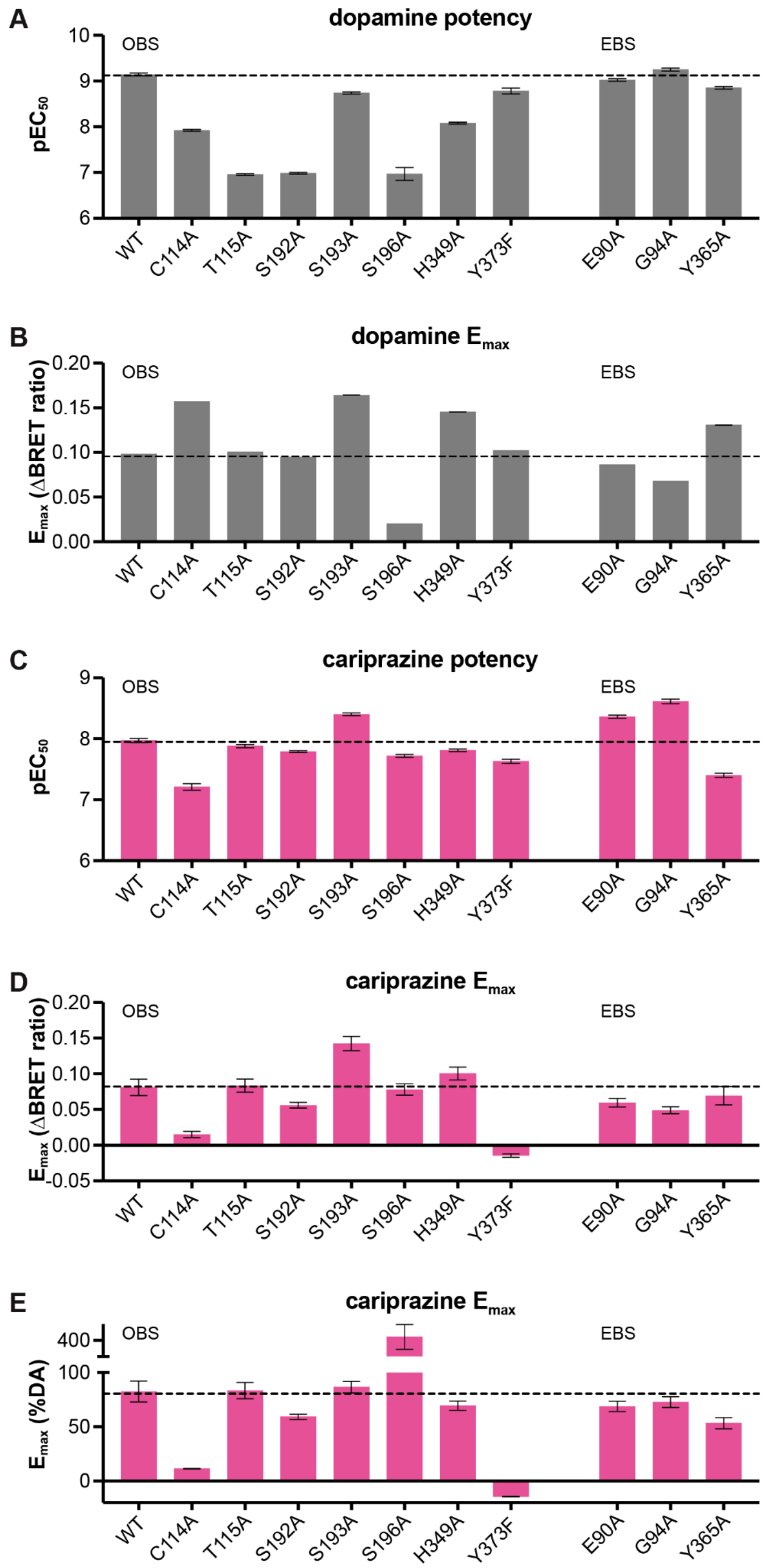

Fig. S6. The potency (pEC_{50}) and maximal effect (E_{max}) of dopamine and cariprazine at mutant D_3Rs. The activities of dopamine (grey) (**A,B**) or cariprazine (pink) (**C-E**) were measured in HEK293 cells expressing the hD_3R using a BRET assay of $G\alpha_{oA}$ G protein activation. E_{max} data is presented in terms of maximal change in BRET units (ΔBRET) (**B,D**) or as a percentage of the maximal effect of dopamine at that receptor mutant (% DA) (**E**). Mutations are divided into orthosteric binding site (OBS) or extended binding site (EBS) residues. Data presented are the mean ± SEM of three individual experiments performed in duplicate. Data is also presented in **table S2.**

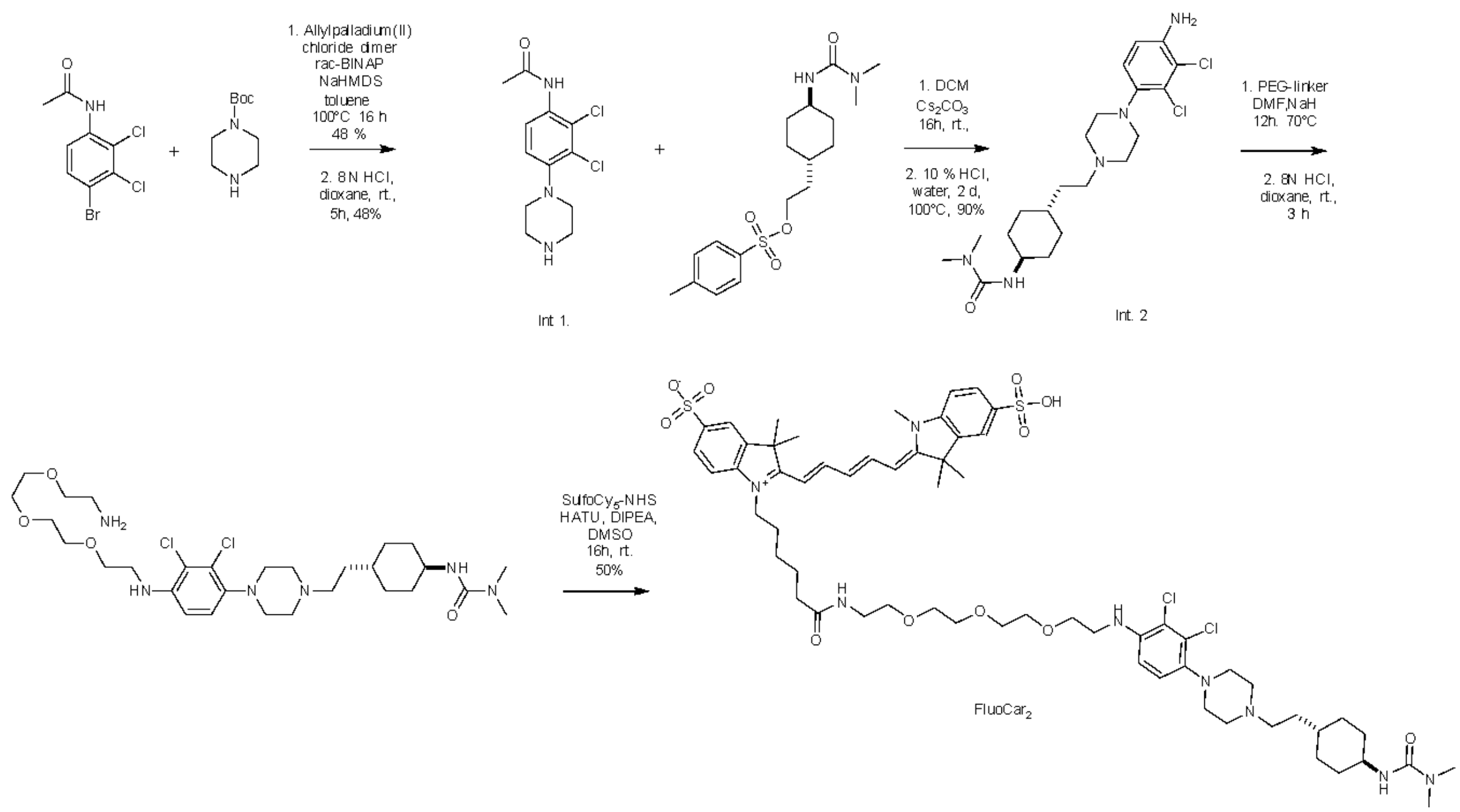

Fig. S7. The synthesis of Fluo2Car and its intermediates. Chemical synthesis of Fluo2Car is detailed in the Supplementary Text and in (*93*).

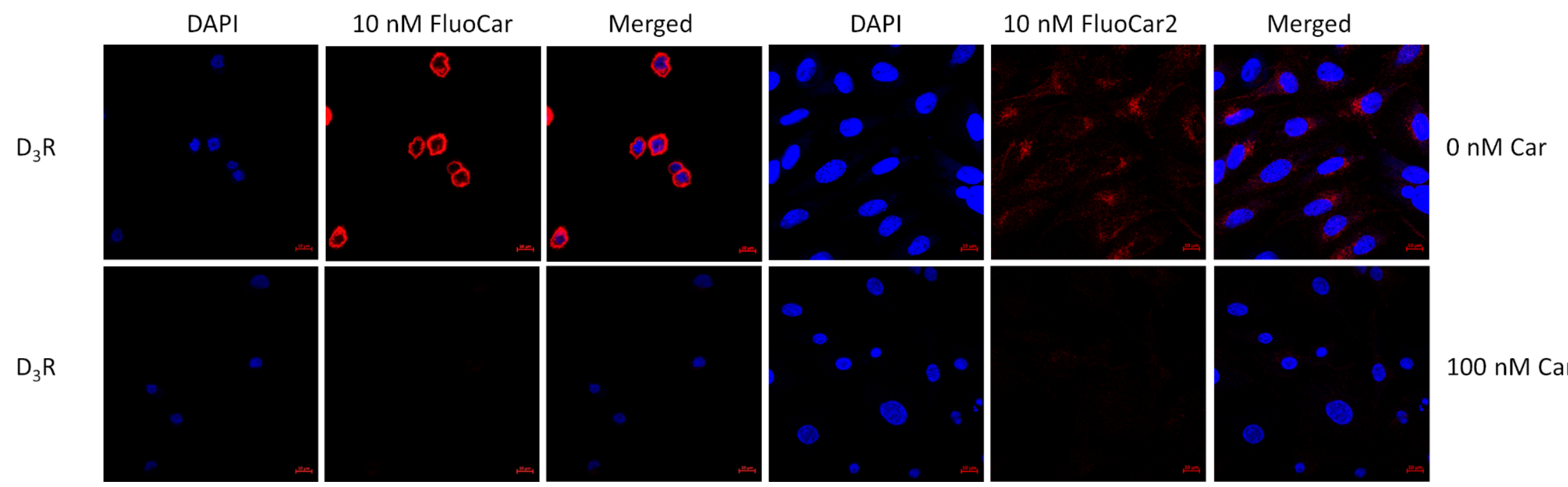

Fig. S8. Confocal microscopy imaging of FluoCar and Fluo2Car on D_3R expressing cells in the absence or presence of 100 nM cariprazine. DAPI was used to color the cells as reference.

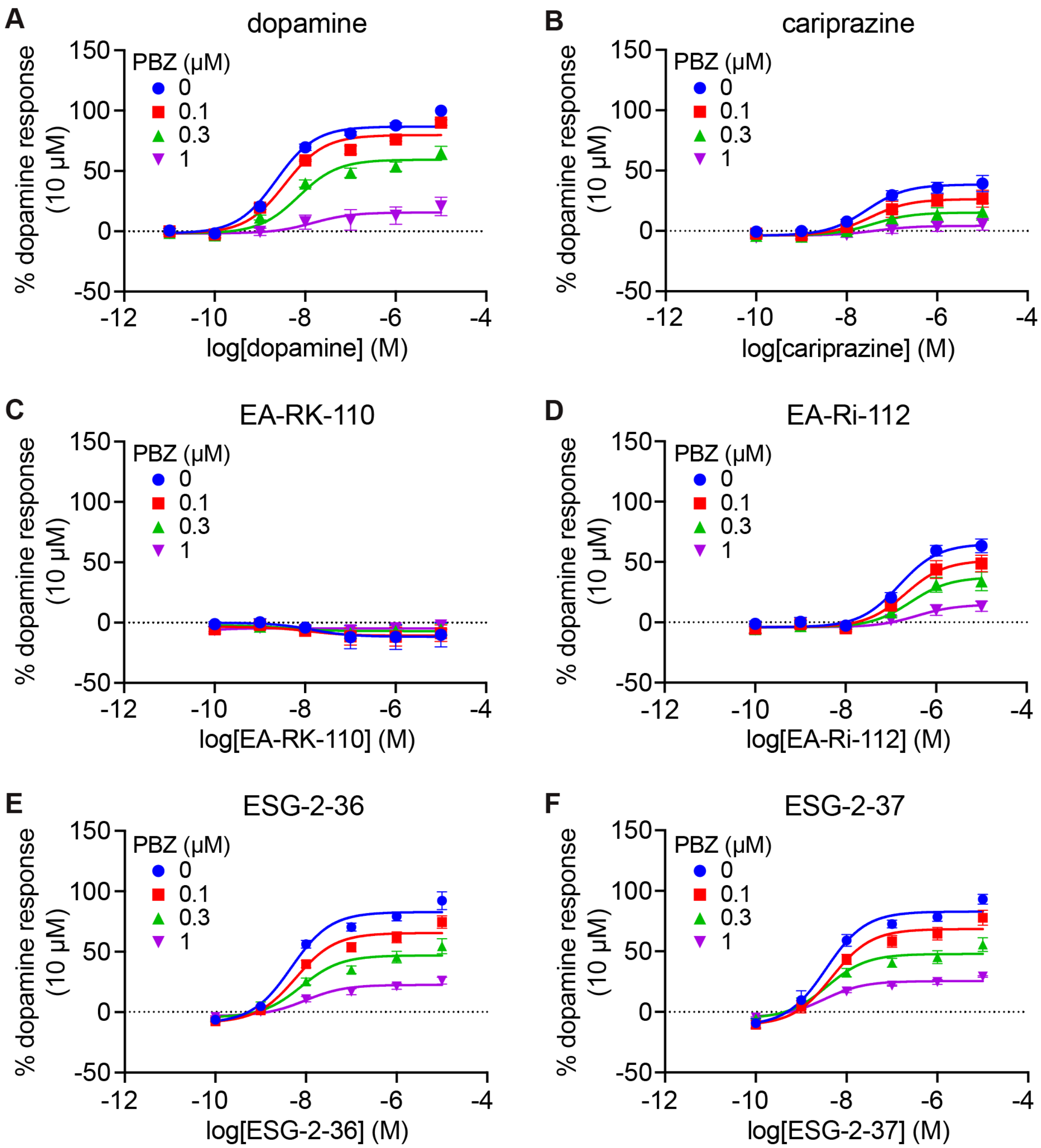

Fig. S9. Measurement of the intrinsic efficacy of the different ligands for D_3R-mediated activation of $G\alpha_{oA}$. The ability of (**A**) dopamine, (**B**) cariprazine, (**C**) EA-RK-110, (**D**) EA-Ri-112, (**E**) ESG-2-36, and (**F**) ESG-2-37 to stimulate $G\alpha_{oA}$ G protein activation measured in a BRET assay in HEK293 cells expressing the D_3R in the absence or presence of increasing concentrations of the irreversible antagonist phenoxybenzamine. Data presented are the mean ± SEM of three individual experiments performed in duplicate. Data were fitted to an operational model of agonism and parameters are presented in **table S3**.

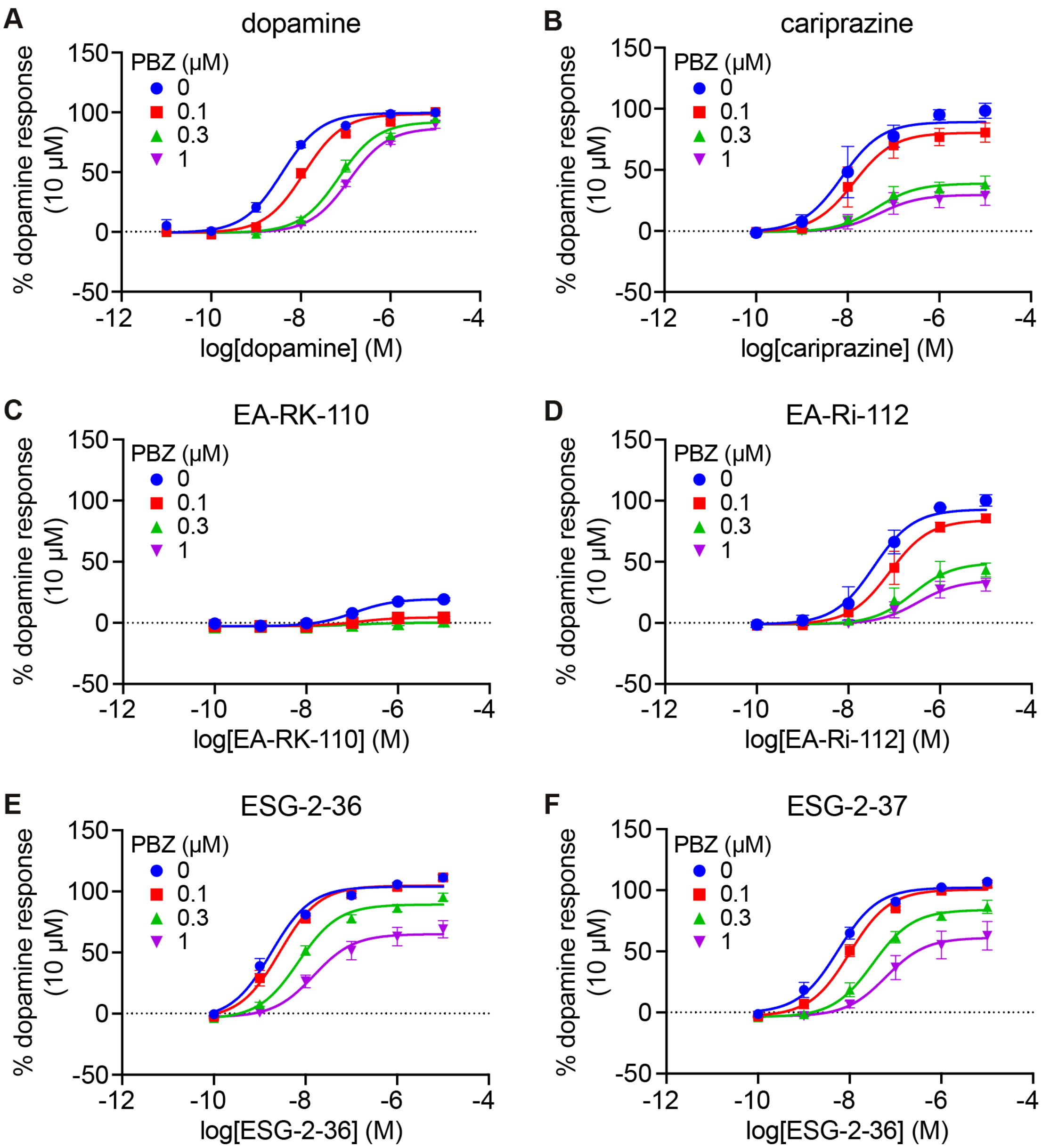

Fig. S10. Measurement of the intrinsic efficacy of the different ligands for D_2R-mediated activation of $G\alpha_{oA}$. The ability of (**A**) dopamine, (**B**) cariprazine, (**C**) EA-RK-110, (**D**) EA-Ri-112, (**E**) ESG-2-36, and (**F**) ESG-2-37 to stimulate $G\alpha_{oA}$ G protein activation measured in a BRET assay in HEK293 cell expressing the D_2R in the absence or presence of increasing concentrations of the irreversible antagonist phenoxybenzamine. Data presented are the mean ± SEM of three individual experiments performed in duplicate. Data were fitted to an operational model of agonism and parameters are presented in **table S4**.

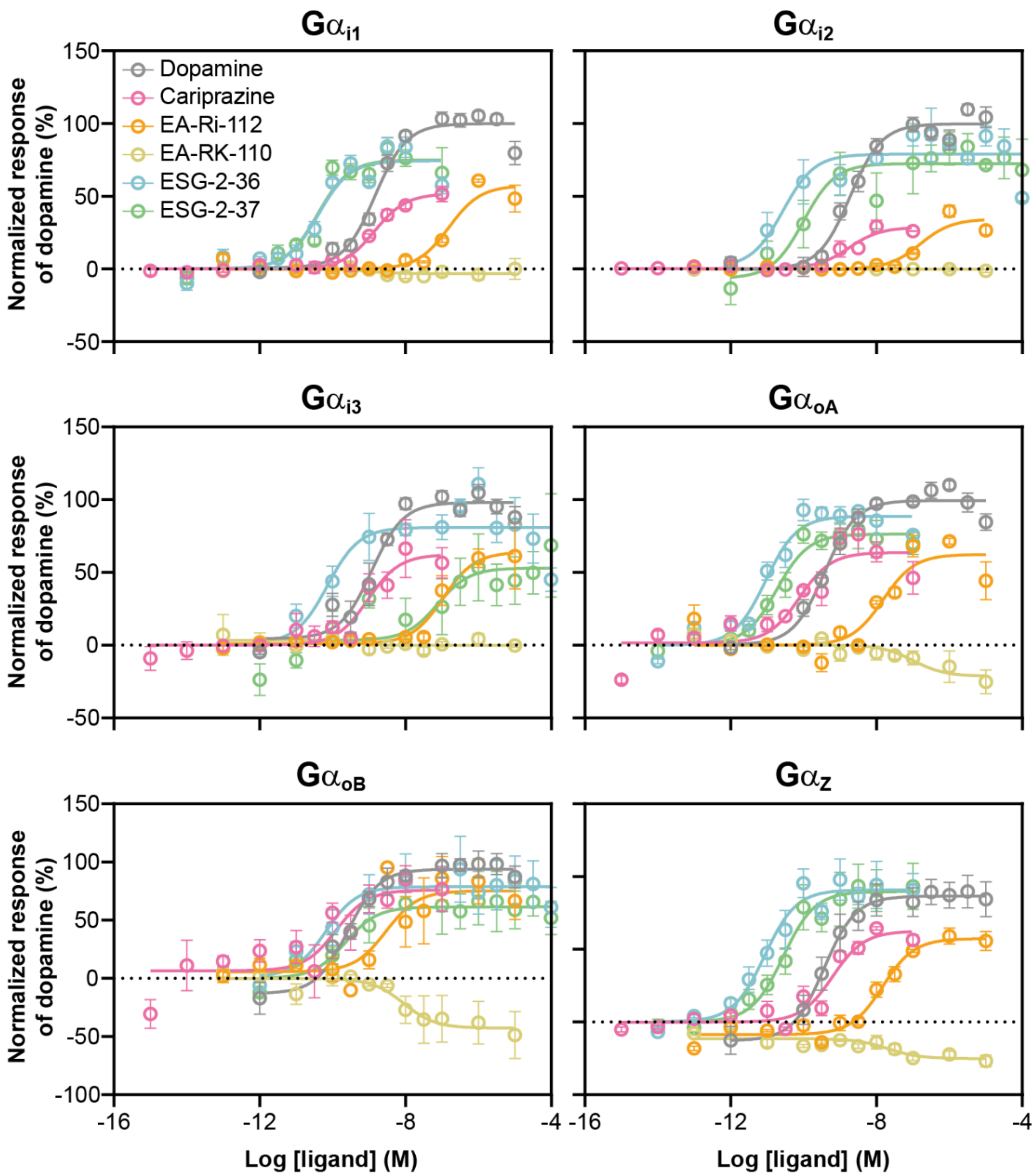

Fig. S11. EMTA BRET assay of the different ligands measuring D_3R-mediated activation of Gαi$_{1\text{-}3}$, αo$_{A/B}$ or α$_Z$. The ability of increasing concentrations of dopamine (grey), cariprazine (pink) and cariprazine derivatives (EA-Ri-112 (orange), EA-RK-110 (yellow), ESG-2-36 (blue) & ESG-2-37 (green) in an EMTA BRET assay, measuring D_3R-mediated activation of Gαi$_{1\text{-}3}$, αo$_{A/B}$ or α$_Z$ as described in methods as the G protein profiling BRET assay. Data were fitted to a three-parameter concentration response curve. Parameters from these experiments are displayed in **table S5** and in the heat map in **Figure 3D**. Data represents the mean ± S.E.M of 3 independent experiments.

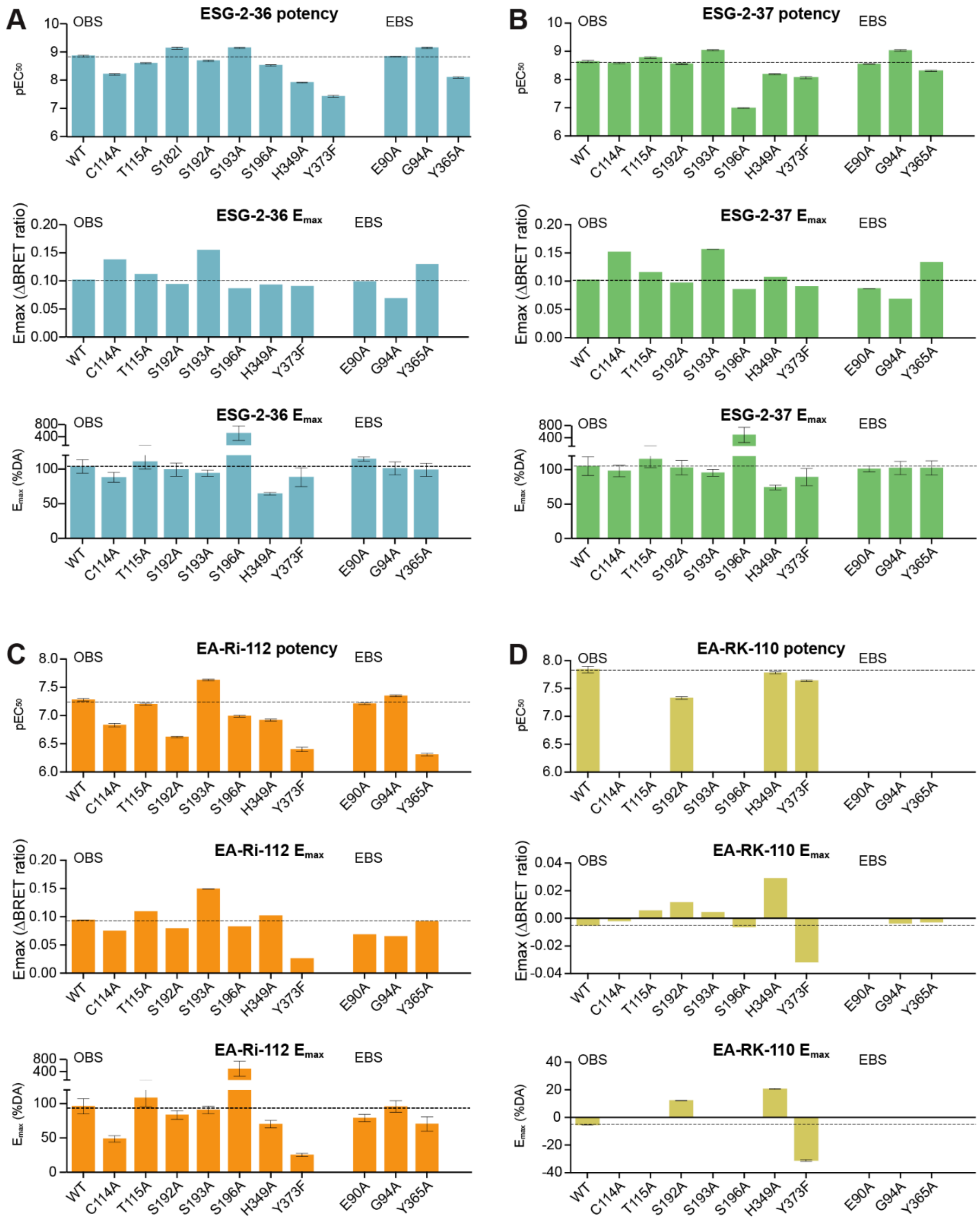

Fig. S12. The potency (pEC_{50}) and maximal effect (E_{max}) of cariprazine derivatives at mutant D_3Rs. The activities of cariprazine derivatives (**A**) ESG-2-36, (**B**) ESG-2-37, (**C**) EA-Ri-112, and (**D**) EA-RK-110 were measured in HEK293 cells expressing the WT or mutant hD_3Rs using a BRET assay of $G\alpha_{oA}$ G protein activation. E_{max} data is presented in terms of maximal change in BRET units (middle panels) or as a percentage of the maximal effect of dopamine at that receptor mutant (% DA, bottom panels). Mutations are divided into orthosteric binding site (OBS) or extended binding site (EBS) residues. Data presented are the mean ± SEM of three individual experiments performed in duplicate. Data is also presented in **tables S6, and S7**.

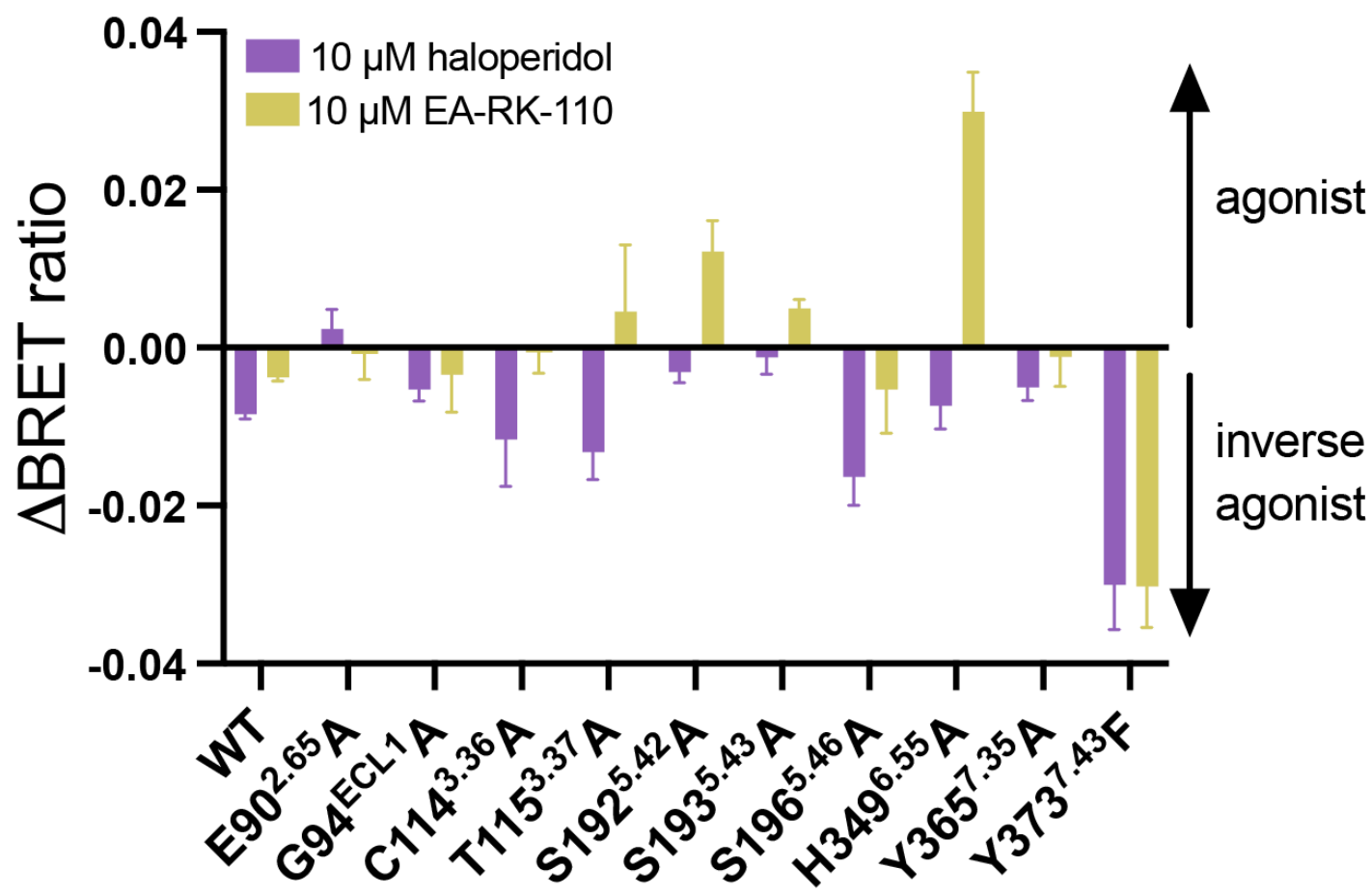

Fig. S13. The effect of EA-RK-110 and the inverse agonist haloperidol at mutant D_3Rs. The action of 10 mM haloperidol (purple bars) or 10 mM EA-RK-110 (yellow bars) were measured in HEK293 cells expressing WT or mutant hD_3Rs using a BRET assay of Ga_{oA} G protein activation (see methods). Values less than zero indicate inverse agonism, values greater than zero indicate agonism. Data presented are the mean ± SEM of three individual experiments performed in duplicate.

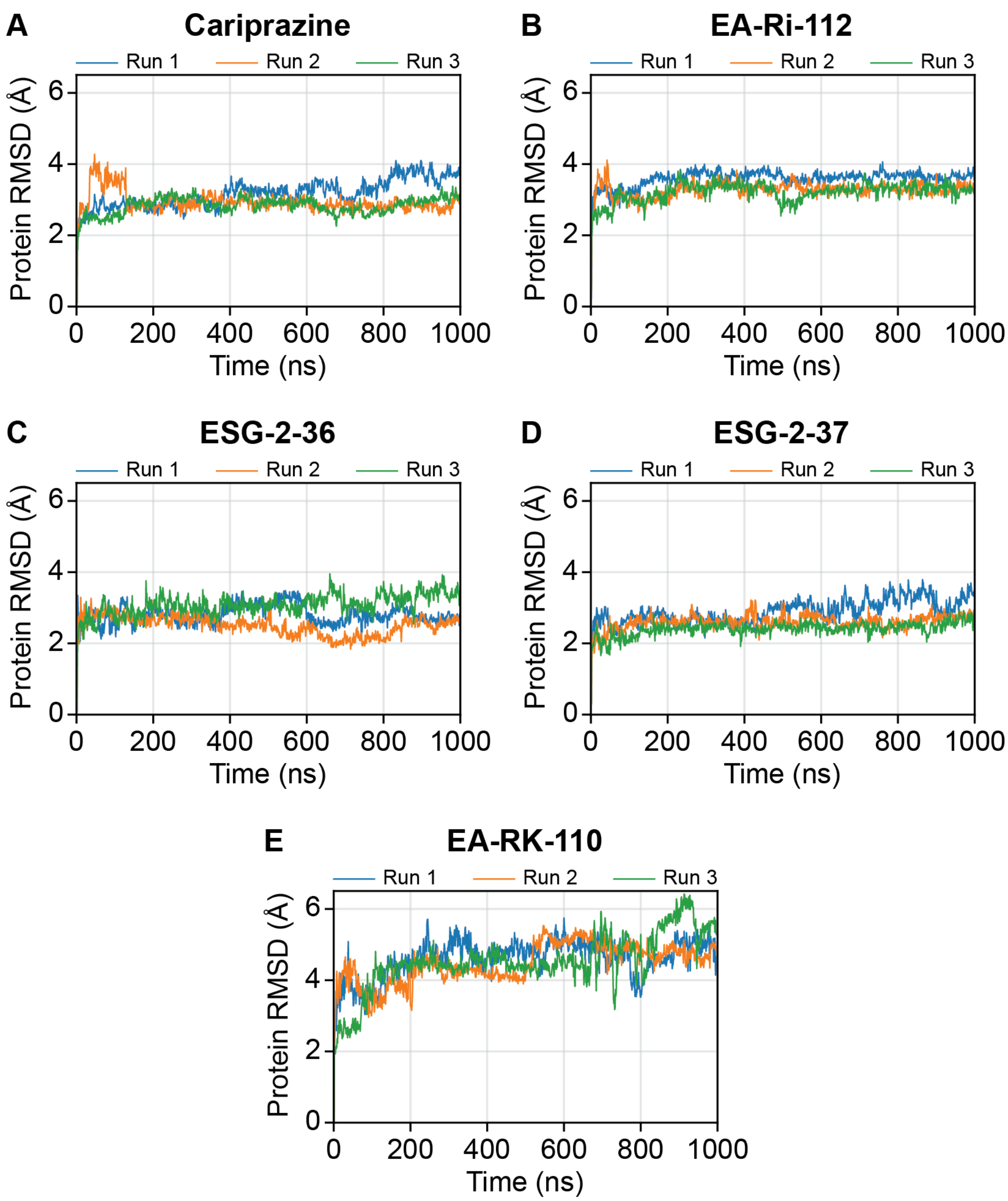

Fig. S14. Backbone RMSD of the receptor during the parallel MD simulations for (**A**) cariprazine, (**B**) EA-Ri-112, (**C**) ESG-2-36, (**D**) ESG-2-37 and (**E**) EA-RK-110. After an initial stabilization period the receptor backbone remains stable during the simulations.

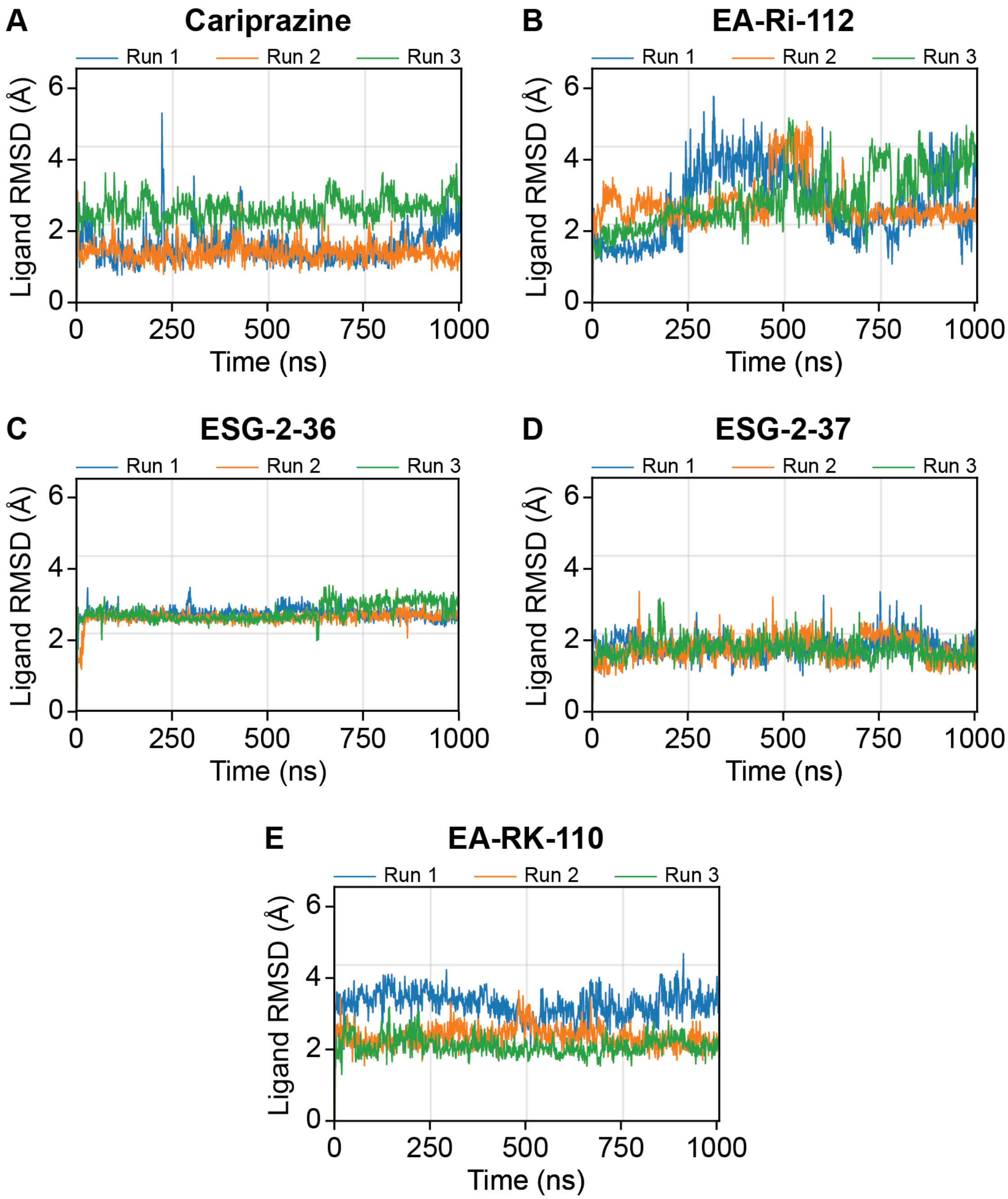

Fig. S15. Ligand RMSD during the parallel MD simulations for (**A**) cariprazine, (**B**) EA-Ri-112, (**C**) ESG-2-36, (**D**) ESG-2-37 and (**E**) EA-RK-110. All ligands maintained the cryo-EM pose during the simulations and remained stable for the 1 us. The largest fluctuations were observed with EA-Ri-112 that contains a bulky tBu-carbamate head group which is not stabilized well in the EBS and moves around more compared to the other investigated ligands. ESG-2-36 and ESG-2-37 were the most stable during the three parallel simulations.

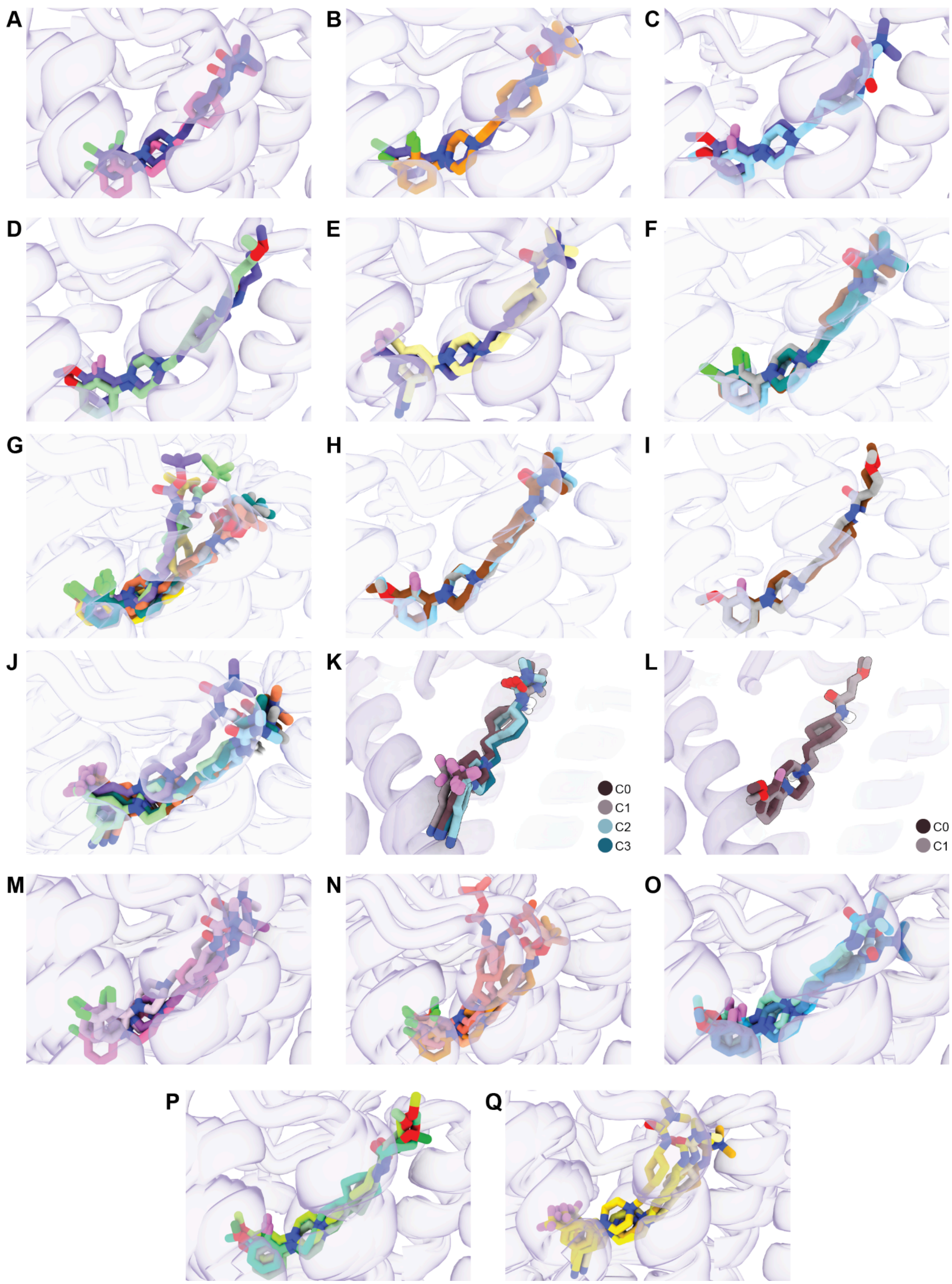
A
B
C
D
E
F
G
H
I
J
K
L
M
N
O
P
Q
C0
C1
C2
C3
C0
C1

Fig. S16. MD simulation. (**A-E**) Alignment of the ligand binding poses in the representative frames obtained with clustering from the MD simulations (purple) and the cryo-EM structures. Both the agonists cariprazine (A), EA-Ri-112 (B), ESG-2-36 (C), ESG-2-37 (D), and the antagonist EA-RK-110 (E) maintain a similar binding pose compared to the starting cryo-EM pose during the MD simulations. Representative frames are the centroids of the largest clusters, obtained via complete linkage clustering after aligning receptor backbones and clustering by ligand heavy-atom RMSD with a 3.0 Å cutoff. (**F-L**) Representative cluster centroids showing the flexibility of the ligands for cariprazine (F), EA-Ri-112 (G), ESG-2-36 (H), ESG-2-37 (I) and EA-RK-110 (J), and the flexibility of the primary pharmacophore for EA-RK-110 (K) and ESG-2-37 (L). Primary pharmacophore clustering (for K-L) used the same method as full ligand clustering (A-J) but on the substituted phenylpiperazine heavy atoms. Clusters covered >75% of frames, except for EA-Ri-112 (G), which was highly flexible in the EBS (41 clusters); the top 8 clusters (~62% of frames) are shown for clarity. Colours in panels (F-L) represent the different clusters. (**M-Q**) Alignment of the ligand binding poses from the last frame of each MD simulation and the cryo-EM structures for cariprazine (M), EA-Ri-112 (N), ESG-2-36 (O), ESG-2-37 (P), and EA-RK-110 (Q).

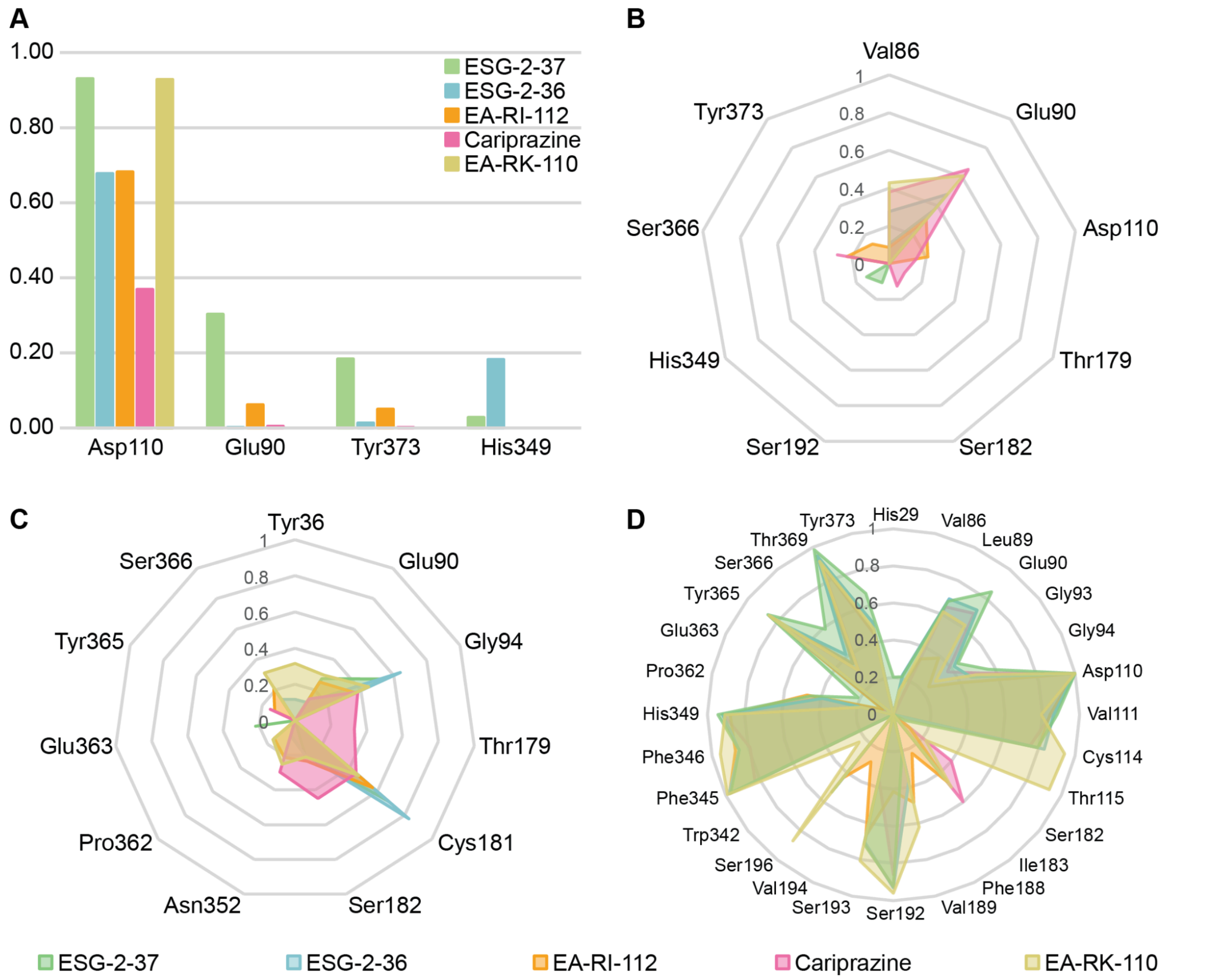

Fig. S17. Protein-ligand interactions from MD. The mean frequency of the most abundant (**A**) direct, (**B**) water mediated, and (**C**) multiple water mediated hydrogen bond interactions between the ligands and the receptor residues, and (**D**) the most abundant residues contacted by the ligands based on Van-der-Waals interactions. Both agonists and antagonists are stabilized by hydrophobic interactions, especially in the orthosteric and linker region, most frequently with L89$^{2.64x63}$, V111$^{3.33x33}$, C114$^{3.36x36}$, F345$^{6.51x51}$, F346$^{6.52x52}$, H349$^{6.55x55}$, Y365$^{7.35x34}$, T369$^{7.39x38}$, and less often Y373$^{7.43x42}$. Direct hydrogen bonds with D110$^{3.32x32}$ anchor the ligands; although least frequent for cariprazine (39%), the ionic interaction remains during simulations even for this compound. For ESG-2-37, additional direct hydrogen bonds with E90$^{2.65x64}$ and Y373$^{7.43x42}$ contribute to stability, while for ESG-2-36, hydrogen bonding with H349$^{6.55x55}$ provides further stability to the primary pharmacophore, albeit to a lesser extent. Water-mediated hydrogen bonds variably stabilize ligand binding, particularly in the extended pocket, mainly via E90$^{2.65x64}$, S366$^{7.36x35}$, V86$^{2.61x60}$, C181$^{45.50x50ECL2}$, T179^{ECL2}, S182$^{45.51x51ECL2}$, G94$^{2.69}$ and N352$^{6.58x58}$, Y365$^{7.35x34}$ and Y36$^{1.39x39}$. Direct interaction with H29$^{1.32x32}$ on TM1 is unique to ESG-2-37. On TM5 S192$^{5.42x43}$ and S193$^{5.43x44}$ is engaged by most ligands, the latter is least abundant for cariprazine though in the investigated series that in contrast to the other agonists engages more ECL2 (I183$^{45.52x52}$, S182$^{45.51x51}$) residues. S196$^{5.46x461}$ and T115$^{3.37x37}$ engagement is characteristic to the antagonist EA-RK-110, while due to its bulky secondary pharmacophore L89$^{2.64x63}$, E90$^{2.65x64}$ and G93$^{2.68x67}$ is less frequently contacted by this ligand.

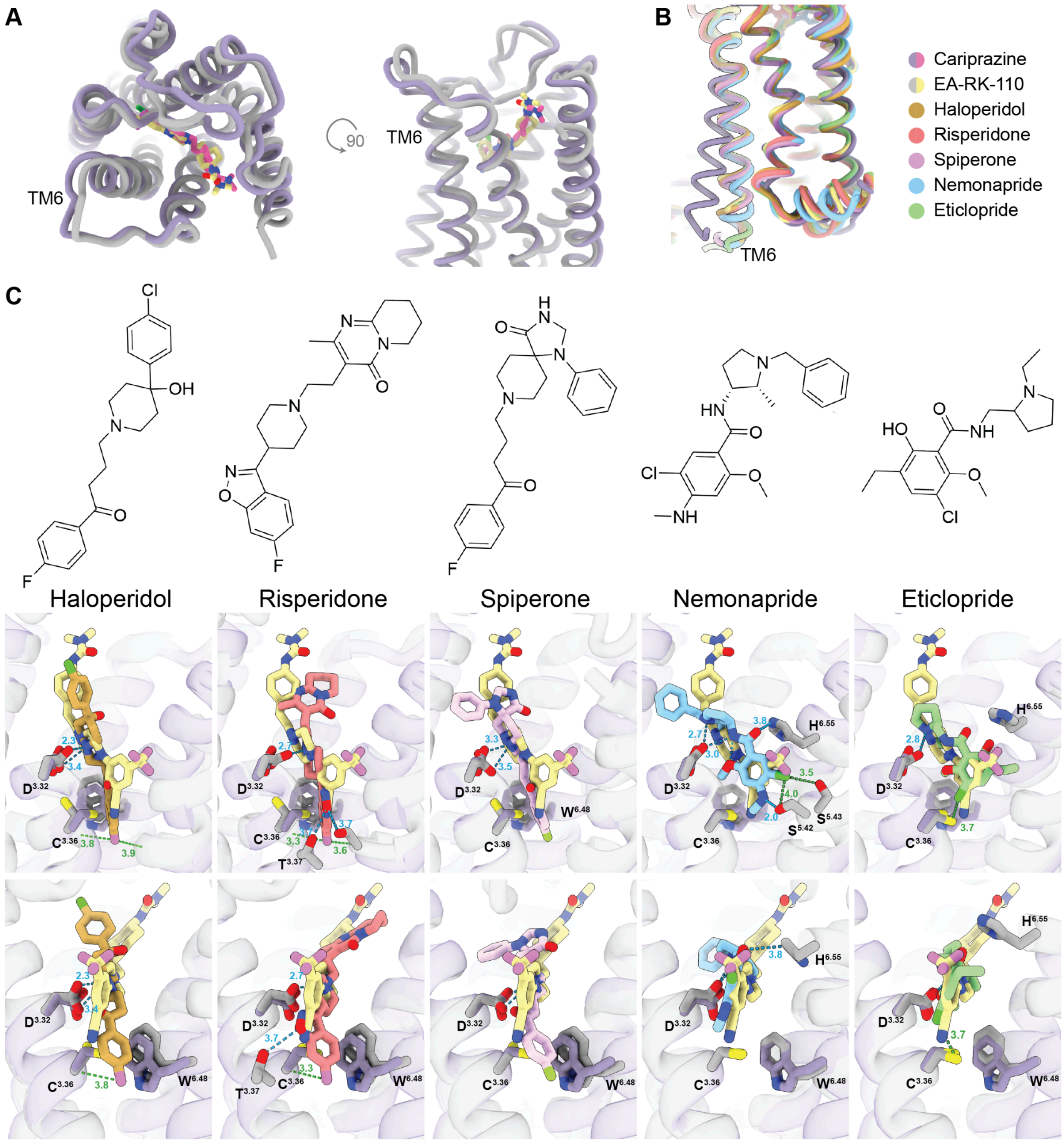

Fig. S18. **Active *vs.* inactive conformational changes of dopamine receptors.** (**A**) The extracellular side of D_3R in the active (D_3R-cariprazine, purple and pink) and inactive (D_3R-EA-RK-110, grey and yellow) states. The extracellular tip of TM6 moves inwards in the inactive state, while ECL2 moves downwards, creating a more compact conformation. (**B**) TM6 in the activated receptor (purple) compared to the inactive states of other dopamine receptors (colors indicated in figure). The intracellular portion of the TM moves inwards. (**C**) Inverse agonist ligand structures (upper panels) and their position within the binding pocket of other dopamine receptors (bottom panels) in comparison to EA-RK-110 (Purple and yellow): D_2R-Haloperidol (PDB 6LUQ (*70*), brown), D_2R-Risperidone (PDB 6CM4 (*74*), dark pink), D_2R-Spiperone (PDB 7DFP (*72*), light pink), D_4R-Nemonapride (PDB 5WIU, 5WIV (*73*), blue), and D_3R-Eticlopride (PDB 3PBL (*33*) , green). Lengths are shown in Å.

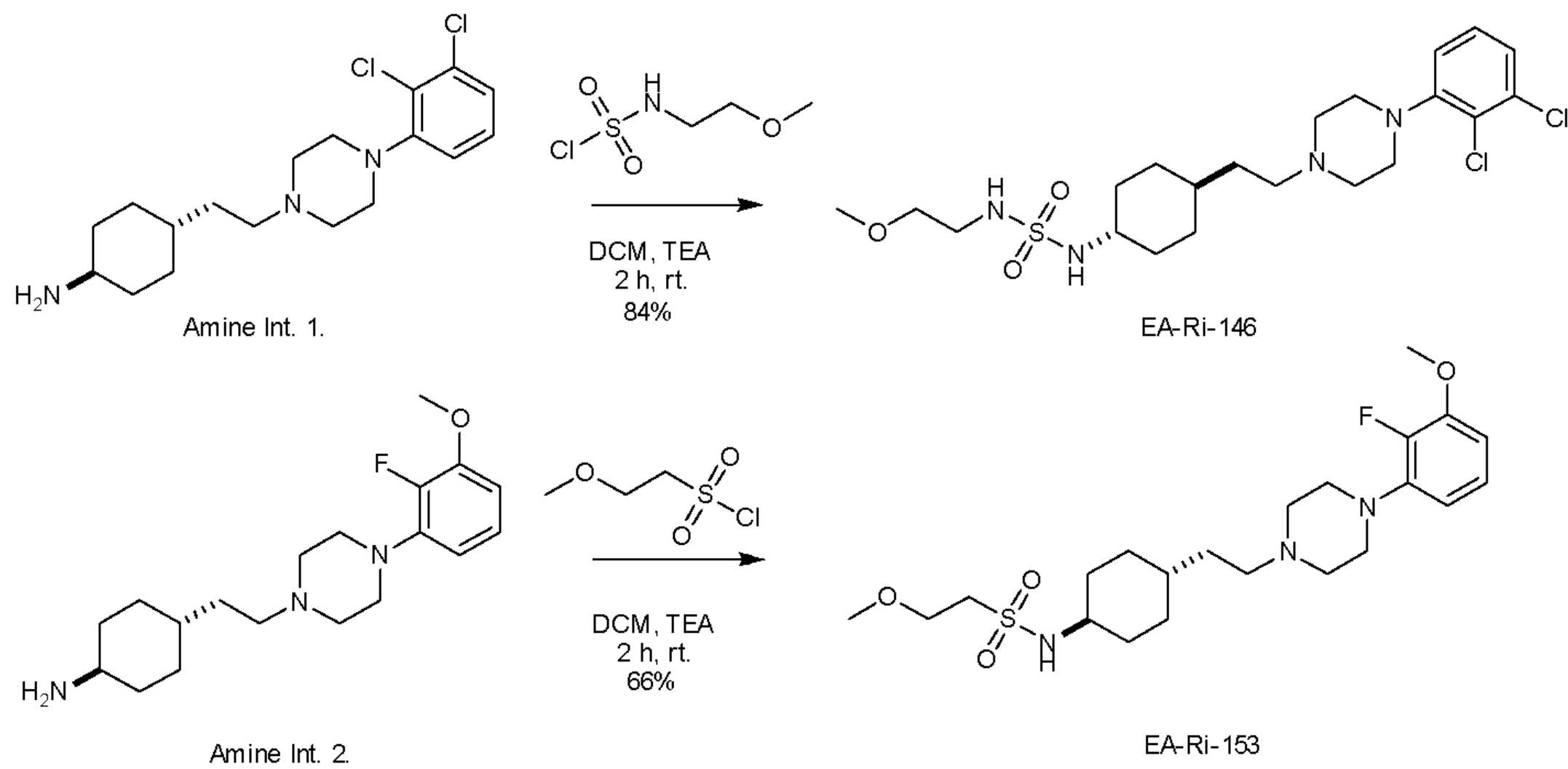

Fig. S19. **Synthesis of EA-Ri-146 (top) and EA-Ri-153 (bottom) from known intermediates.** Chemical syntheses of the two compounds are detailed in the Supplementary Text.

Table S1. Cryo-EM data collection, processing, refinement and validation.

Data collection	D_3R - Cariprazine	D_3R - EA-Ri-112	D_3R - ESG-2-36	D_3R - ESG-2-37	D_3R - EA-RK-110
**Microscope**	Titan Krios G3i				
**Detector**	Gatan K3				
**Energy filter slit width [eV]**	20				
**Nominal magnificat'n [fold]**	105,000				
**Voltage [kV]**	300				
**Electron exposure [e^-/Å^2]**	38				
**Exposure rate [e^-/(Å^2.s)]**	25.5	26.3	25.9	26.3	26.1
**Defocus range [µm]**	-0.8 – (-2.2)				
**Pixel size [Å]**	0.8423				
**Number of frames per movie**	45				
**Automation software**	EPU				
**Data processing**					
**Processing software**	cryoSPARC/RELION				cryoSPARC
**Micrographs [no.]**	4,941	4,395	5,405	6,029	15,824
**Initial particles [no.]**	5,941,290	5,149,002	6,543,064	7,503,220	13,954,333
**Total # of particles used for 3D analyses**	1,495,738	204,408	3,057,485	3,644,983	985,089
**Final particles [no.]**	247,597	175,447	113,825	138,474	273,679
**Map resolution (FSC 0.143) [Å]**	2.91	3.03;<br>R: 3.39 ; G: 2.9	2.85	2.74<br>R: 2.91 ; G: 2.65	3.58
**Symmetry**	P1	P1	P1	P1	P1
**Model Refinement**					
**Refinement package**	Phenix				
**Initial model (PDB code)**	7CMV, 8E9X				7CMV, 6USF, 4EIY
**Map sharpening B factor [Å^2]**	109.6	101.2<br>R: 119.2 ; G: 97.9	82.1	83.2<br>R: 77.0 ; G: 77.5	140.8

	D_3R - Cariprazine	D_3R - EA-Ri-112	D_3R - ESG-2-36	D_3R - ESG-2-37	D_3R - EA-RK-110
**Model composition**					
**Non-hydrogen atoms**	8367	8627	8606	8733	5947
**Protein residues**	1118	1131	1120	1129	801
**Ligands**	1	1	1	1	1
**B factors [Å^2]**					
**Protein**	75.84	132.36	118.01	98.06	176.61
**Ligand**	118.64	165.23	132.32	84.59	184.05
**R.M.S.D**					
**Bond lengths [Å]**	0.03	0.004	0.006	0.003	0.006
**Bond angles [°]**	0.585	0.800	0.643	0.609	0.697
**Global and Local CC [CC_{volume}/CC_{mask}]**	0.81/0.83	0.79/0.79	0.82/0.83	0.8/0.8	0.77/0.77
**C-beta deviations [%]**	0	0	0	0	0
**CaBLAM outliers [%]**	2	2.1	0.9	1.1	3.5
**Validation**					
**MolProbity score**	0.87	1.43	1.08	1.13	1.54
**Clashscore**	0.98	4.07	2.88	3.35	5.49
**Poor rotamers [%]**	0.23	0	0.22	0.21	0.16
**Ramachandran plot**					
**Favored [%]**	97.64	96.41	98.1	98.11	96.32
**Allowed [%]**	2.36	3.59	1.9	1.89	3.68
**Disallowed [%]**	0	0	0	0	0
**PDB codes**	9T8C	9T7Q	9T7P	9T88	9T8D

Table S2. The potency (pEC_{50}) and maximal effect (E_{max}) of cariprazine at WT or mutant D_3Rs. Data were obtained in a $G\alpha_{oA}$ protein activation assay using HEK293 cells transiently expressing the WT or mutant D_3R. Fold change in potency is determined relative to WT. E_{max} is displayed as change in BRET in arbitrary units. For cariprazine E_{max} is also displayed as a percentage of the E_{max} of dopamine at each receptor mutant. Change in E_{max} is determined by subtracting E_{max} (as % of dopamine E_{max}) value at WT from the E_{max} obtained at each mutant. Data presented are the mean ± SEM of three individual experiments performed in duplicate.

	**dopamine (DA)**			**cariprazine**				
	**pEC_{50} ± 95% CI (EC_{50}, nM)**	**Fold change**	**E_{max} DBRET ± 95% CI**	**pEC_{50} ± 95% CI (EC_{50}, nM)**	**Fold change**	**E_{max} DBRET ± 95% CI**	**E_{max} (% DA) ± 95% CI**	**difference *vs.* WT (%)**
**WT**	9.14 ± 0.34 (0.7)	-	0.10 ± 0.01	7.97 ± 0.44 (10.7)	-	0.08 ± 0.01	82.5 ± 11.7	-
**$E90^{2.65x64}A$**	9.02 ± 0.29 (0.9)	1.3	0.09 ± 0.01	8.36 ± 0.32 (4.4)	0.4	0.06 ± 0.01	68.7 ± 7.1	-13.8
**$G94^{ECL1}A$**	9.25 ± 0.35 (0.6)	0.8	0.07 ± 0.01	8.61 ± 0.44 (2.4)	0.2	0.05 ± 0.01	72.7 ± 6.8	-9.8
**$C114^{3.36x36}A$**	7.92 ± 0.23 (11.9)	16.6	0.15 ± 0.01	7.21 ± 0.75 (61.6)	5.8	0.02 ± 0.00	11.4 ± 2.7	-71.2
**$T115^{3.37x37}A$**	6.96 ± 0.28 (110.3)	153.3	0.10 ± 0.01	7.88 ± 0.34 (13.3)	1.2	0.08 ± 0.01	83.3 ± 9.0	0.8
**$S192^{5.42x43}A$**	6.98 ± 0.26 (103.7)	144.1	0.09 ± 0.01	7.79 ± 0.19 (16.2)	1.5	0.06 ± 0.01	59.2 ± 4.1	-23.3
**$S193^{5.43x44}A$**	8.74 ± 0.26 (1.8)	2.5	0.16 ± 0.01	8.40 ± 0.25 (4.0)	0.4	0.14 ± 0.01	86.6 ± 6.1	4.1
**$S196^{5.46x461}A$**	6.97 ± 2.02 (107.2)	149.0	0.02 ± 0.02	7.72 ± 0.30 (19.2)	1.8	0.08 ± 0.01	464.0 ± 49	381
**$H349^{6.55x55}A$**	8.08 ± 0.25 (8.3)	11.6	0.15 ± 0.01	7.81 ± 0.26 (15.4)	1.4	0.10 ± 0.01	69.4 ± 6.2	-13.1
**$Y365^{7.35x34}A$**	8.85 ± 0.32 (1.4)	2.0	0.13 ± 0.01	7.40 ± 0.45 (39.5)	3.7	0.07 ± 0.01	53.3 ± 9.8	-29.3
**$Y373^{7.43x42}F$**	8.78 ± 0.72 (1.6)	2.3	0.10 ± 0.02	7.63 ± 0.45 (23.2)	2.2	-0.01 ± 0.00	-14.4 ± 2.2	-96.9

Table S3. **The binding affinities and intrinsic efficacies of dopamine, cariprazine and cariprazine derivatives at the hD_3R.** Binding affinity (K_i) was determined in a TR-FRET binding assay using a fluorescent version of spiperone. The functional affinity (K_A) and relative intrinsic efficacy (**τ**) were determined for dopamine, cariprazine and cariprazine derivatives in an assay measuring $G\alpha_{oA}$ G-protein activation by the D_3R (**fig. S9**). Data presented are the mean ± SEM of three individual experiments.

Compound	pK_i (K_i, nM)	pK_A (K_A, nM)	Log*τ*	*τ*	% of dopamine *τ*
**dopamine**	7.97 ± 0.12 (11)	7.74 ± 0.04 (18 nM)	0.82 ± 0.03	6.72 ± 0.47	100
**cariprazine**	8.97 ± 0.09 (1.1)	7.45 ± 0.04 (35 nM)	-0.17 ± 0.12	0.73 ± 0.20	10.9 ± 2.5
**EA-RK-110**	No agonism detectable				
**EA-Ri-112**	7.62 ± 0.11 (24)	6.36 ± 0.17 (437 nM)	0.30 ± 0.11	2.16 ± 0.53	31.6 ± 5.2
**ESG-2-36**	7.36 ± 0.10 (44)	7.63 ± 0.11 (23 nM)	0.65 ± 0.11	4.90 ± 1.03	73 ± 15
**ESG-2-37**	8.14 ± 0.16 (7.2)	7.82 ± 0.14 (15 nM)	0.63 ± 0.08	4.56 ± 0.95	68 ± 14

Table S4. **The functional affinity (K_A) and relative intrinsic efficacy (τ)** of dopamine, cariprazine and cariprazine derivatives determined in an assay measuring $G\alpha_{oA}$ G-protein activation by the D_2R (**fig. S10**). Data presented are the mean ± SEM of three individual experiments.

Compound	pK_A (K_A, nM)	Logτ	τ	% of dopamine τ
**Dopamine**	6.01 ± 0.07 (977 nM)	2.40 ± 0.08	261 ± 46	100
**EA-RK-110**	6.84 ± 0.11 (145 nM)	-0.55 ± 0.03	0.27 ± 0.02	0.1 ± 0.03
**Cariprazine**	7.07 ± 0.19 (85 nM)	1.00 ± 0.17	11.4 ± 3.9	4.4 ± 1.3
**EA-Ri-112**	6.28 ± 0.26 (524 nM)	1.17 ± 0.10	15.6 ± 3.1	6.3 ± 1.7
**ESG-2-36**	7.06 ± 0.09 (73 nM)	1.78 ± 0.06	61.6 ± 9.0	24 ± 0.3
**ESG-2-37**	6.55± 0.15 (287 nM)	1.82 ± 0.16	84.9 ± 38	33 ± 0.1

Table S5. The potency (pEC_{50}) and maximal effect (E_{max}, as % of dopamine response) of cariprazine and derivatives at the D_3R determined in a EMTA BRET assay of G protein activation (**fig. S11**). Data presented are the mean ± SEM of three individual experiments.

Agonist Mode		CARIPRAZINE	EA-Ri-112	EA-RK-110	ESG-2-36	ESG-2-37
$G\alpha_{i1}$	pEC_{50}	8.85 ± 0.10	6.81 ± 0.13	NA	10.39 ± 0.14	10.41 ± 0.15
	E_{max}	51.78 ± 2.92	57.42 ± 3.70	NA	74.14 ± 4.80	74.77 ±5.21
$G\alpha_{i2}$	pEC_{50}	8.76 ± 0.14	6.80 ± 0.13	NA	10.56 ± 0.14	10.00 ± 0.33
	E_{max}	28.47 ± 2.18	34.36 ± 3.70	NA	77.77 ± 4.80	78.91 ±11.90
$G\alpha_{i3}$	pEC_{50}	9.03 ± 0.23	6.96 ± 0.19	NA	10.14 ± 0.32	7.12 ± 0.58
	Emax	62.23 ± 7.47	63.06 ± 6.69	NA	79.89 ± 11.93	49.67 ±11.55
$G\alpha_{oA}$	pEC_{50}	10.11 ± 0.25	7.82 ± 0.20	*6.99 ± 0.38**	11.04 ± 0.11	10.73 ± 0.14
	E_{max}	62.21 ± 6.76	62.45 ± 6.02	*-21.48 ± 4.23**	88.74 ± 4.79	76.15 ± 4.97
$G\alpha_{oB}$	pEC_{50}	9.90 ± 0.33	8.53 ± 0.39	*8.05 ± 0.40**	10.19 ± 0.44	9.65 ± 0.48
	E_{max}	69.35 ± 10.71	69.43 ± 10.75	*-42.57 ± 8.03**	77.79 ± 15.83	61.3 ± 11.93
$G\alpha_z$	pEC_{50}	9.20 ± 0.13	7.83 ± 0.10	*7.69 ± 0.44**	11.07 ± 0.15	10.56 ± 0.17
	E_{max}	62.54 ± 4.02	66.01 ± 3.30	*-13.72 ± 3.03**	90.62 ± 6.70	89.51 ± 7.09

inverse agonist

Table S6. The potency (pEC_{50}) and maximal effect of EA-Ri-112 or EA-RK-110 at WT or mutant D_3Rs. Data were obtained in a Ga_{oA} protein activation assay using HEK293 cells transiently expressing the WT or mutant D_3R. Fold change in potency is determined relative to WT. E_{max} is displayed as change (Δ) in BRET in arbitrary units or as a percentage of the E_{max} of dopamine at each receptor mutant. Change in E_{max} is determined by subtracting E_{max} (as % of dopamine E_{max}) value at WT from the E_{max} obtained at each mutant. Data presented are the mean ± SEM of three individual experiments performed in duplicate.

	EA-Ri-112					EA-RK-110				
	**pEC_{50} ± 95% CI (EC_{50}, nM)**	**Fold change**	**E_{max} ΔBRET ± 95% CI**	**E_{max} (% DA) ± 95% CI**	**difference vs WT (%)**	**pEC_{50} ± 95% CI (EC_{50}, nM)**	**Fold change**	**E_{max} ΔBRET ± 95% CI**	**E_{max} (% DA) ± 95% CI**	**difference vs WT (%)**
**WT**	7.28 ± 0.31 (52.2)	-	0.09 ± 0.01	96.0 ± 11.7	-	7.84 ± 0.74 (14.5)	-	-0.005 ± 0.001	-5.33 ± 1.1	-
**E90$^{2.65x64}$A**	7.21 ± 0.20 (61.0)	1.17	0.07 ± 0.01	79.0 ± 6.6	-17.0	-	-	-	-	-
**G94^{ECL1}A**	7.35 ± 0.23 (45.0)	0.86	0.07 ± 0.01	95.8 ± 8.6	-0.2	-	-	-	-	-
**C114$^{3.36x36}$A**	6.83 ± 0.49 (149.2)	2.86	0.07 ± 0.02	48.7 ± 9.7	-47.3	-	-	-	-	-
**T115$^{3.37x37}$A**	7.20 ± 0.28 (63.1)	1.21	0.11 ± 0.01	108.4 ± 12.2	12.5	-	-	-	-	-
**S192$^{5.42x43}$A**	6.62 ± 0.20 (242.5)	4.65	0.08 ± 0.01	83.4 ± 7.7	-12.5	7.33 ± 0.32 (46.8)	3.2	0.011 ± 0.003	12.2 ± 1.4	17.5
**S193$^{5.43x44}$A**	7.63 ± 0.20 (23.4)	0.45	0.15 ± 0.01	90.7 ± 6.2	-5.2	-	-	-	-	-
**S196$^{5.46x461}$A**	6.99 ± 0.23 (103.0)	1.97	0.08 ± 0.01	492.3 ± 50.8	396.4	-	-	-	-	-
**H349$^{6.55x55}$A**	6.92 ± 0.27 (120.3)	2.31	0.10 ± 0.01	70.2 ± 7.6	-25.7	7.78 ± 0.22 (16.6)	1.1	0.029 ± 0.004	20.6 ± 1.4	26.0
**Y365$^{7.35x34}$A**	6.31 ± 0.37 (488.4)	9.36	0.09 ± 0.02	70.4 ± 15.0	-25.6	-	-	-	-	-
**Y373$^{7.43x42}$F**	6.40 ± 0.62 400.3	7.67	0.03 ± 0.01	25.5 ± 9.3	-70.5	7.64 ± 0.19 (23.2)	1.6	0.03 ± 0.01	-31.1 ± 2.0	-25.8

Table S7. The potency (pEC_{50}) and maximal effect of ESG-2-36 or ESG-2-27 at WT or mutant D_3Rs. Data were obtained in a $G\alpha_{oA}$ protein activation assay using HEK293 cells transiently expressing the WT or mutant D_3R. Fold change in potency is determined relative to WT. E_{max} is displayed as change (Δ) in BRET in arbitrary units or as a percentage of the E_{max} of dopamine at each receptor mutant. Change in E_{max} is determined by subtracting E_{max} (as % of dopamine E_{max}) value at WT from the E_{max} obtained at each mutant. Data presented are the mean ± SEM of three individual experiments performed in duplicate.

	ESG-2-36					ESG-2-37				
	pEC_{50} ± 95% CI (EC_{50}, nM)	Fold change	E_{max} ΔBRET ± 95% CI	E_{max} (% DA) ± 95% CI	difference vs WT (%)	pEC_{50} ± 95% CI (EC_{50}, nM)	Fold change	E_{max} ΔBRET ± 95% CI	E_{max} (% DA) ± 95% CI	difference vs WT (%)
**WT**	8.86 ± 0.33 (1.4)	-	0.10 ± 0.01	103.8 ± 9.3	-	8.64 ± 0.43 (2.3)	-	0.10 ± 0.02	105.3 ± 13.0	-
**E90$^{2.65x64}$A**	8.84 ± 0.08 (1.4)	1.0	0.10 ± 0.00	114.6 ± 2.7	10.9	8.55 ± 0.13 (2.8)	1.22	0.09 ± 0.00	101.1 ± 4.1	-4.2
**G94^{ECL1}A**	9.15 ± 0.36 (0.7)	0.5	0.07 ± 0.01	101.0 ± 9.3	-2.8	9.03 ± 0.34 (0.9)	0.41	0.07 ± 0.01	102.5 ± 9.5	-2.8
**C114$^{3.36x36}$A**	8.21 ±0.30 (6.2)	4.5	0.14 ± 0.01	88.1 ± 8.1	-15.7	8.58 ± 0.31 (2.6)	1.14	0.15 ± 0.02	98.3 ± 8.5	-7.0
**T115$^{3.37x37}$A**	8.60 ± 0.32 (2.5)	1.8	0.11 ± 0.01	110.8 ± 9.8	7.1	8.78 ± 0.35 (1.7)	0.73	0.11 ± 0.01	115.5 ± 10.7	10.1
**S192$^{5.42x43}$A**	8.69 ± 0.34 (2.0)	1.5	0.09 ± 0.01	99.3 ± 9.6	-4.5	8.56 ± 0.32 (2.7)	1.19	0.09 ± 0.01	102.9 ± 10.2	-2.4
**S193$^{5.43x44}$A**	9.15 ± 0.19 (0.7)	0.5	0.15 ± 0.01	94.1 ± 4.9	-9.6	9.04 ± 0.19 (0.9)	0.40	0.15 ± 0.01	95.6 ± 5.1	-9.8
**S196$^{5.46x461}$A**	8.53 ± 0.30 (3.0)	2.1	0.09 ± 0.01	518.2 ± 46.0	414.5	6.99 ± 0.23 (103.0)	44.71	0.09 ± 0.01	492.3 ± 50.8	387.0
**H349$^{6.55x55}$A**	7.92 ± 0.14 (12.1)	8.8	0.09 ± 0.00	64.3 ± 3.1	-39.4	8.19 ± 0.20 (6.4)	2.78	0.09 ± 0.00	74.3 ± 4.6	-31.0
**Y365$^{7.35x34}$A**	8.10 ± 0.29 (7.9)	5.8	0.13 ± 0.01	99.1 ± 9.6	-4.7	8.31 ± 0.31 (4.9)	2.11	0.13 ± 0.01	102.6 ± 10.2	-2.7
**Y373$^{7.43x42}$F**	7.43 ± 0.46 (37.0)	26.9	0.09 ± 0.02	88.41± 5.5	-15.4	8.07 ± 0.49 (8.5)	3.68	0.09 ± 0.02	89.31 ± 3.9	-16.1

Table S8. The binding affinities and functional activities of cariprazine and newly designed cariprazine analogues. Binding affinities (K_i) at the dopamine and serotonin receptors were determined in competition binding studies and functional activities obtained from BRET assay (pEC_{50} and E_{max}) on the D_3R Data presented are the mean ± SEM of three individual experiments. [a] Parameters taken from Egyed et al (*35*).

		cariprazine[a]	EA-Ri-146	EA-Ri-153
	**Structure**	-	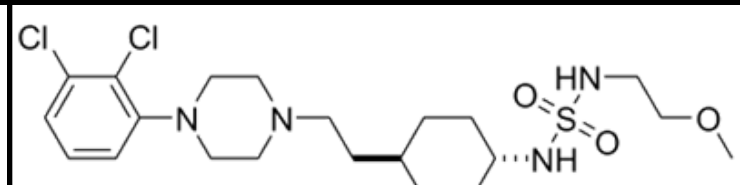	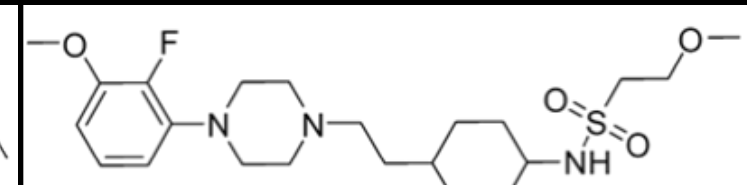
**Ki (nM)**	**D_2 ([^{3}H]-spiperone))**	1.58	9.80	1220.00
	**D_3 ([^{3}H]7-OH-DPAT)**	2.03	0.02	2.80
	**$5HT_{1A}R$ ([^{3}H]-8-OH-DPAT) ± SD**	-	196 ± 6	2513 ± 525
	**$5HT_{2A}R$ ([^{3}H]-ketanserin) ± SD**	-	699 ± 45	22835 ± 417
	**D_3 selectivity**	0.77	490.00	435.71
**D3 Ga_{OA} activation**	**pEC_{50} (95%CI)**	-	8.91 (8.46-9.34)	8.90 (8.69-9.10)
	**E_{max} (95% CI)**	-	76.0 (67.0-85.2)	107.4 (101.0-113.8)

Table S9. High-Performance Liquid Chromatography (HPLC) measurement details for the Blood–Brain Barrier Parallel Artificial Membrane Permeability Assay (BBB PAMPA).

<table>
<tr><th>HPLC</th><td colspan="3">Shimadzu</td></tr>
<tr><th>Detector</th><td colspan="3">DAD</td></tr>
<tr><th>Column</th><td colspan="3">Kinetex C18 50x4.6 mm 2.6 um - FA (H23-221666)</td></tr>
<tr><th>Column temperature</th><td colspan="3">40°C</td></tr>
<tr><th>Flow rate</th><td colspan="3">0.5 mL/min</td></tr>
<tr><th rowspan="2">Eluents</th><th>A:</th><td colspan="2">95% H_2O, 5% ACN, 0.1% FA</td></tr>
<tr><th>B:</th><td colspan="2">95% ACN, 5% H_2O, 0.1% FA</td></tr>
<tr><th rowspan="7">Gradients</th><th>Time (min)</th><th>A %</th><th>B%</th></tr>
<tr><td>0.0</td><td>90</td><td>10</td></tr>
<tr><td>1.0</td><td>90</td><td>10</td></tr>
<tr><td>3.0</td><td>10</td><td>90</td></tr>
<tr><td>5.0</td><td>10</td><td>90</td></tr>
<tr><td>5.5</td><td>90</td><td>10</td></tr>
<tr><td>10.5</td><td>90</td><td>10</td></tr>
</table>

Table S10. BBB sample HPLC measurement (Raw data). Sample preparation was performed using 190 µL ACN and 10 µL of a 10 mM DMSO stock solution.

Compound	Wavelength (nm)	RT (min)	AREA
**EA-Ri-146**	250	5.258	986070
**EA-Ri-153**	250	5.080	79606
**CARBAZEPIN**	290	5.544	3355630
**INDOMETACIN**	290	6.323	2416364

Table S11. BBB permeability raw data obtained using the Blood-Brain Barrier Parallel Artificial Membrane Permeability Assay (BBB PAMPA). Donor buffer: 15 µL of a 10 mM DMSO stock solution and 285 µL PBS (pH 7.4) buffer.

	50 uL + 12.5 uL ACN		100 uL		80 uL + 20 uL ACN	
	DONOR		ACCEPTOR		Starting solution	
	RT (min)	AREA	RT (min)	AREA	RT (min)	AREA
EA-Ri-146	5.26	51774.00	5.27	33431.00	5.26	411793.00
	5.26	41049.00	5.26	20736.00	5.24	294655.00
	-	-	5.26	33150.00	5.23	343733.00
EA-Ri-153	5.07	20568.00	5.07	8761.00	5.05	58359.00
	5.07	16601.00	5.07	7571.00	-	-
	5.07	20812.00	5.07	10369.00	5.04	55127.00
CARBAZEPIN	5.54	1270692.00	5.53	517131.00	5.54	1891037.00
	5.54	1277702.00	5.53	921895.00	5.55	2736614.00
	5.55	2460078.00	5.53	39119.00	5.55	2440456.00
INDOMETACIN	6.31	2045144.00	6.29	21571.00	6.32	1914333.00
	6.31	2004865.00	6.30	31060.00	6.31	1915757.00
	6.31	2087992.00	6.30	7462.00	6.32	1890763.00

Supplementary Text

Chemical Synthesis.

All starting materials were purchased from commercial sources or the synthesis was published earlier (*20*, *64*). Compounds **EA-Ri-112**, **EA-RK-110** and **cariprazine** were synthesized based on our former publication (*42*). For the experiments with the **FluoCar**, we used the same batch that was published earlier (*9*). HPLC chromatograms of all synthesised compounds are published for purity verification. All synthesized new compounds were identified with HPLC-MS, HRMS and NMR measurements.

Synthesis of Fluo2Car and its intermediates (fig. S7)

N-[2,3-Dichloro-4-(piperazin-1-yl)phenyl]acetamide hydrochloride (Int.1)

Allylpalladium(II) chloride dimer (65 mg, 0.176 mmol), *rac*. BINAP (220 mg, 0.35 mmol), *tert*-butyl piperazine-1-carboxylate (790 mg, 4.24 mmol) and *N*-(4-bromo-2,3-dichlorophenyl)acetamide (1.0 g, 3.53 mmol) were dissolved in 10 mL of abs. toluene under argon atmosphere. NaHMDS (4.5 mL, 4.5 mmol, 1 M in THF) was added dropwise to the mixture in 10 minutes, at room temperature. The solution was heated to 100 °C and stirred for 16 hours. The mixture was concentrated under reduced pressure, the residue was redissolved in DCM (50 mL) and extracted with water (100 mL). The organic layer was concentrated, and the remaining crude product was (950 mg, 2.45 mmol) was suspended in 10 mL of 8 M HCl in dioxane solution. The mixture was stirred at room temperature for 5 hours. The precipitate was filtered and washed with diethyl ether. The crude product was purified with flash chromatography (eluents: hexane and EtOAc). 532 mg (48%) white solid was obtained. HRMS $[M+H]^+$ $C_{12}H_{16}N_3OCl_2$ expected: 288.0667 measured: 288.0670. ^{1}H NMR (500 MHz, DMSO-d_6) δ 9.67 (s, 1H), 9.50 (s, 1H), 7.57 (d, J = 8.8 Hz, 1H), 7.20 (d, J = 8.8 Hz, 1H), 4.01 – 3.69 (m, 4H), 3.48 – 3.14 (m, 4H), 2.08 (s, 3H). ^{13}C NMR (126 MHz, DMSO-d_6) δ 185.12, 169.14, 147.09, 132.76, 127.24, 125.94, 119.64, 66.83, 48.33, 43.51.

3,3-Dimethyl-1-[(1*R*,4*R*)-4-{2-[4-(4-amino-2,3-dichlorophenyl)piperazin-1-yl]ethyl}cyclohexyl] urea (Int. 2)

Step 1. 2-[(1*R*,4*R*)-4-[(Dimethylcarbamoyl)amino]cyclohexyl]ethyl 4-methylbenzene-1-sulfonate (140 mg, 0.382 mmol), Cs_2CO_3 (170 mg, 0.52 mmol) and **Int. 1** (100 mg, 0.347 mmol) were dissolved in 10 mL of DCM. The mixture was stirred for 16 hours at room temperature, then it was concentrated under reduced pressure, the residue was redissolved in DCM (5 mL) and extracted with water (5 mL). The organic layer was concentrated, and the remaining crude product was transferred to the next reaction step without further purification.

Step 2. The crude product of step 1. (25 mg, 0.051 mmol) was dissolved in 3 mL of 10% aqueous hydrochloric acid solution. The mixture was stirred for 2 days at 100 °C. The pH of the solution was adjusted to 8 with saturated Na_2CO_3 solution and extracted with DCM. The organic phase was dried over $MgSO_4$, filtered and evaporated. The crude product was purified with preparative HPLC (eluents: water and CH_3CN, both containing 0.1% formic acid). 20 mg (90%) yellowish-white solid was obtained. HRMS $[M+H]^+$ $C_{21}H_{34}N_5OCl_2$ expected: 442.2134 measured: 442.2142. ^{1}H NMR (500 MHz, DMSO-d_6, TFA-d_1) δ 7.59 – 7.53 (m, 1H), 7.28 (d, J = 8.3 Hz, 1H)., 3.59 (d, J = 11.9 Hz, 2H), 3.44 (d, J = 12.8 Hz, 2H), 3.37 (td, J = 11.5, 5.8 Hz, 1H), 3.18 (dt, J = 20.5, 8.9 Hz, 4H), 3.03 (t, J = 12.2 Hz, 2H), 2.75 (s, 6H), 1.81 – 1.70 (m, 4H), 1.57

(dt, J = 11.7, 6.8 Hz, 2H), 1.21 (qd, J = 12.4, 3.0 Hz, 3H), 1.05 – 0.95 (m, 2H). ^{13}C NMR (126 MHz, DMSO-d_6, TFA-d_1) δ 149.33, 137.90, 132.77, 128.40, 126.13, 125.26, 119.70, 54.01, 51.23, 49.29, 47.85, 35.74, 34.34, 32.52, 31.35, 30.06.

Fluo2Car

(1-(1-((2,3-dichloro-4-(4-(2-((1R,4R)-4-(3,3-dimethylureido)cyclohexyl)ethyl)piperazin-1-yl)phenyl)amino)-13-oxo-3,6,9-trioxa-12-azaoctadecan-18-yl)-3,3-dimethyl-2-((1*E*,3*E*)-5-((*Z*)-1,3,3-trimethyl-5-sulfoindolin-2-ylidene)penta-1,3-dien-1-yl)-3H-indol-1-ium-5-sulfonate)

Step 1. Sodium hydride (8 mg, 0.2 mmol, 60% dispersion in mineral oil) and **Int. 2** (18 mg, 0.04 mmol) were suspended in 0.2 mL of DMF and stirred for 30 minutes at room temperature. In the next step *tert*-butyl *N*-{2-[2-(2-{2-[(4-methylbenzenesulfonyl)oxy]ethoxy}ethoxy)ethoxy]ethyl}carbamate (21 mg; 0,046 mmol) in 0.1 mL of DMF was added to the reaction mixture. The solution was stirred for 12 hours at 70 °C. The mixture was filtered and purified with preparative HPLC (eluents: water and CH_3CN, both containing 0.1% formic acid). Afterward, the protecting group was removed using 8 M HCl in dioxane. Since only a small amount was available, the compound was used in the next step without further analysis.

Step 2. The PEG linker coupled intermediate (1 mg, 0.0016 mmol) was dissolved in 0.1 mL of DMF, DIPEA (15 µl, 0.005 mmol), HATU (3 mg, 0,005 mmol) and Sulfo-Cy5 NHS ester (4 mg, 0.005 mmol) were added to the solution. The mixture was stirred for 16 hours at room temperature. The mixture was purified with preparative HPLC (eluents: water and CH_3CN, both containing 0.1% formic acid) and lyophilized. 0.8 mg of blue crystals (yield: 50%) were obtained. HRMS $[M+H]^{2+}$ $C_{61}H_{88}Cl_2N_8O_{11}S_2$ expected: 621.2690 measured: 621.2672. ^{1}H NMR (500 MHz, DMSO-d_6) δ 8.49 (s, 14H), 8.35 (t, J = 13.1 Hz, 2H), 7.96 (d, J = 5.3 Hz, 1H), 7.89 (d, J = 13.0 Hz, 2H), 7.81 (s, 2H), 7.65 (ddd, J = 7.5, 5.6, 1.5 Hz, 2H), 7.52 (dd, J = 7.0, 2.0 Hz, 1H), 7.45 – 7.08 (m, 6H), 7.05 (d, J = 7.9 Hz, 2H), 6.88 (d, J = 7.8 Hz, 2H), 6.69 – 6.51 (m, 1H), 6.28 (dd, J = 25.3, 13.8 Hz, 2H), 6.18 (s, 1H), 5.58 (s, 2H), 5.33 (t, J = 5.0 Hz, 1H), 4.28 (t, J = 7.0 Hz, 2H), 4.09 (t, J = 7.2 Hz, 2H), 3.60 (s, 2H), 3.51 – 3.43 (m, 3H), 3.16 (dd, J = 11.1, 5.3 Hz, 2H), 3.00 (q, J = 6.6 Hz, 2H), 2.11 – 2.04 (m, 6H), 1.69 (s, 9H), 1.59 – 1.51 (m, 2H), 1.46 (dd, J = 14.1, 7.0 Hz, 1H), 1.25 (d, J = 3.3 Hz, 9H), 1.08 (dt, J = 17.4, 7.0 Hz, 6H), 0.88 (dt, J = 16.4, 7.2 Hz, 4H).

Synthesis of designed compounds (Top: EA-Ri-146 , Bottom: EA-Ri-153) from known intermediates (fig. S19).

EA-Ri-146

([(4-{2-[4-(2,3-dichlorophenyl)piperazin-1-yl]ethyl}cyclohexyl)sulfamoyl](2-methoxyethyl)amine)

4-{2-[4-(2,3-dichlorophenyl)piperazin-1-yl]ethyl}cyclohexan-1-amine (54 mg, 0.15 mmol) and triethylamine (63 µl, 0.45 mmol) were added to 3 mL of dichloromethane. After 10 minutes stirring, *N*-(2-methoxyethyl)sulfamoyl chloride (52 mg, 0.30 mmol) was added to the solution. The mixture was stirred 2 hours at room temperature. The mixture was extracted with water, dried over $MgSO_4$ filtered and evaporated. The crude product was purified with preparative HPLC (eluents: water and CH_3CN). 62 mg (yield: 84%) white solid was obtained. HRMS $[M+H]^+$ $C_{21}H_{35}Cl_2N_4O_3S$ expected: 493.1801 measured: 493.1808 ^{1}H NMR (500 MHz, CD_3OD) δ 7.27 (q, J = 3.7 Hz, 2H), 7.13 (dd, J = 6.1, 3.5 Hz, 1H), 3.52 (t, J = 5.6 Hz, 2H), 3.38 (s, 3H), 3.19 (s, 4H), 3.14 (t, J = 5.7 Hz, 2H), 3.12 – 3.04 (m, 1H), 3.00 (s, 4H), 2.79 (t, J = 8.2 Hz, 2H), 2.12 – 2.06 (m, 2H), 1.86 (d, J = 13.1 Hz, 2H), 1.57 (dd, J = 10.4, 5.8 Hz, 2H), 1.32 (td, J = 16.0, 6.7 Hz, 4H), 1.17 – 1.05 (m, 2H). ^{13}C NMR (126 MHz, CD_3OD) δ 150.17, 145.05, 140.26, 127.74, 118.89, 98.88, 70.92, 57.57, 52.66, 49.74, 42.72, 35.01, 33.31, 31.72, 14.44, 7.19.

EA-Ri-153

(*N*-(4-{2-[4-(3-fluoro-2-methoxyphenyl)piperazin-1-yl]ethyl}cyclohexyl)-2-methoxyethane-1-sulfonamide)

(1*R*,4*R*)-4-{2-[4-(2-fluoro-3-methoxyphenyl)piperazin-1-yl]ethyl}cyclohexan-1-amine (11 mg, 0.033 mmol) and triethylamine (23 µl, 0.165 mmol) were added to 3 mL of dichloromethane. After 10 minutes of stirring, 2-methoxyethane-1-sulfonyl chloride (16 mg, 0.10 mmol) was added to the solution. The mixture was stirred 2 hours at room temperature. The mixture was extracted with water, dried over $MgSO_4$, filtered and evaporated. The crude product was purified with preparative HPLC (eluents: water and CH_3CN). 10 mg (66%) white solid was obtained. HRMS $[M+H]^+$ $C_{22}H_{36}FN_3O_4S$ expected: 458.2483 measured: 458.2471. ^{1}H NMR (500 MHz, DMSO-d_6) δ 8.17 (s, 1H), 7.00 (qd, J = 5.4, 1.7 Hz, 2H), 6.77 (t, J = 7.9 Hz, 1H), 6.61 (t, J = 7.8 Hz, 1H), 3.79 (s, 3H), 3.64 (t, J = 6.5 Hz, 2H), 3.28 – 3.21 (m, 5H), 3.07 – 2.95 (m, 5H), 2.33 (t, J = 7.5 Hz, 2H), 1.90 – 1.83 (m, 2H), 1.74 – 1.69 (m, 2H), 1.33 (q, J = 7.1 Hz, 2H), 1.25 – 1.12 (m, 4H), 0.96 (qd, J = 13.1, 3.3 Hz, 2H). ^{13}C NMR (126 MHz, DMSO-d_6) δ 148.48, 144.19, 141.13, 124.30, 111.46, 107.53, 66.73, 58.45, 56.53, 56.09, 53.32, 52.74, 52.59, 50.65, 34.86, 34.22, 33.70, 32.07.

Analytical methods.

NMR. NMR spectra were recorded at 30 °C, on a Varian Unity Inova 500 spectrometer (500 MHz for ^{1}H NMR and 125 MHz for ^{13}C spectra) with the deuterium signal of the solvent as the lock. Chemical shifts (δ) and coupling constants (J) are given in ppm and Hz, respectively. The solvent of the NMR measurement is indicated for each substance.

HPLC-MS. HPLC-MS measurements were performed using a Shimadzu LCMS-2020 device equipped with a Reprospher 100 C18 (5 µm; 100×3 mm) column and positive-negative double ion source (DUIS) with a quadrupole MS analyser in a range of 50–1000 *m/z or 300-1500 m/z*. Sample was eluted with gradient elution using eluent A (0.1% formic acid in water) and eluent B (0.1% formic acid in acetonitrile). Flow rate was set to 1.5 mL/min. The initial condition was 0% B eluent, followed by a linear gradient to 100% B eluent by 2 min, from 2 to 3.75 min 100% B eluent was retained; and from 3.75 to 4.5 min back to initial condition and retained to 5 min. The column temperature was kept at 30 °C and the injection volume was 1 µl. Purity determinations were performed using HPLC by the integration of the UV-peaks at 254 nm, the same column and eluents were used as written before. For better separation, the following gradient was utilized: the initial condition was 0% B eluent, followed by a linear gradient to 100% B eluent by 7 min, from 7 to 8 min 100% B eluent was retained, and from 8 to 9 min back to the initial condition and retained to 10 min.

HRMS. HRMS measurements were performed on a Triple TOF 5600+ QTOF mass spectrometer (Sciex, Framingham, MA, USA) equipped with DuoSpray ion source. The resolution of the instrument was above 30 000 over the entire mass range. Samples were introduced into an acetonitrile flow (0.2 mL/min) in flow injection mode. Analyst TF 1.8.1 software (Sciex, Framingham, MA, USA) was used for controlling the instrument and for data processing.

Characterisation of novel compounds

EA-Ri-146 structure:

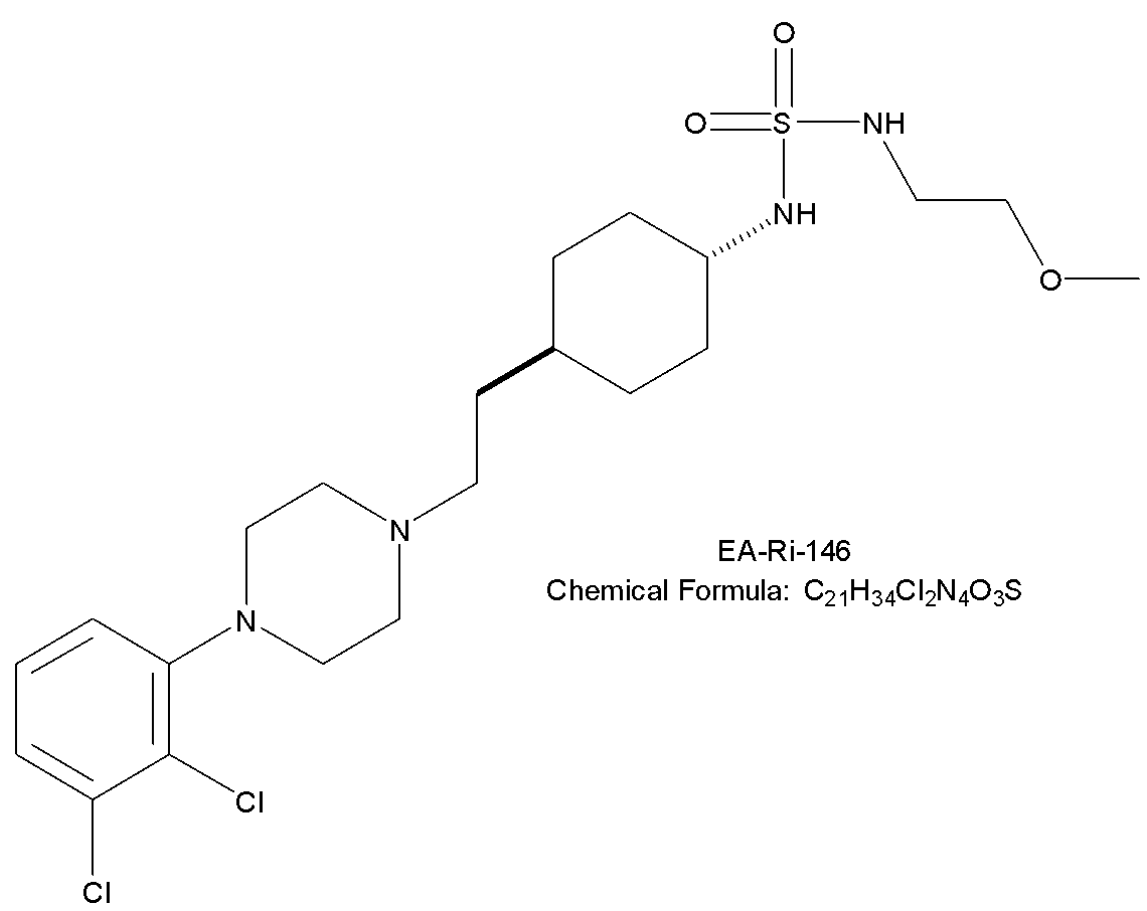

^{1}H NMR spectrum of EA-Ri-146:

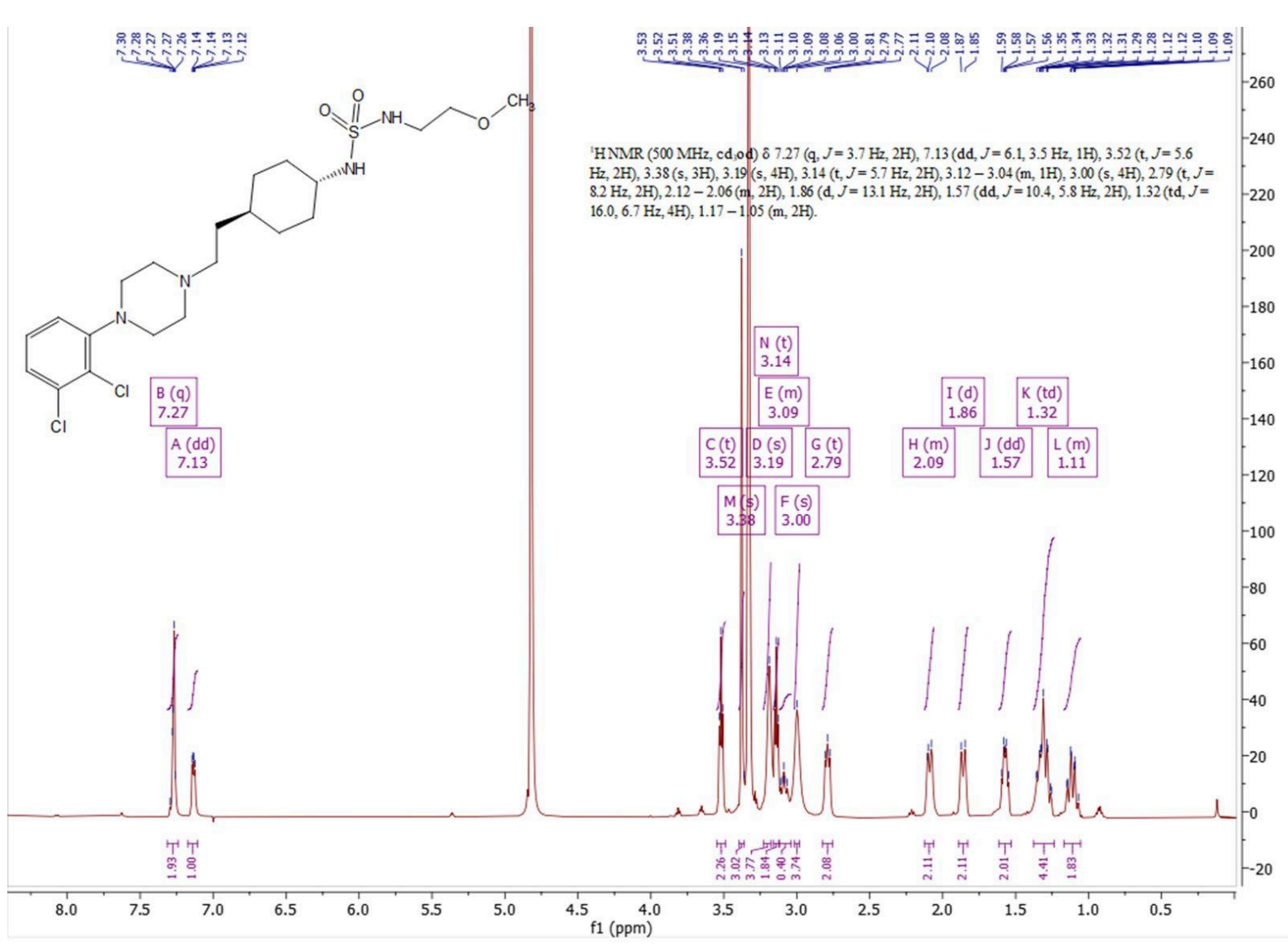

^{13}C NMR spectrum of EA-Ri-146:

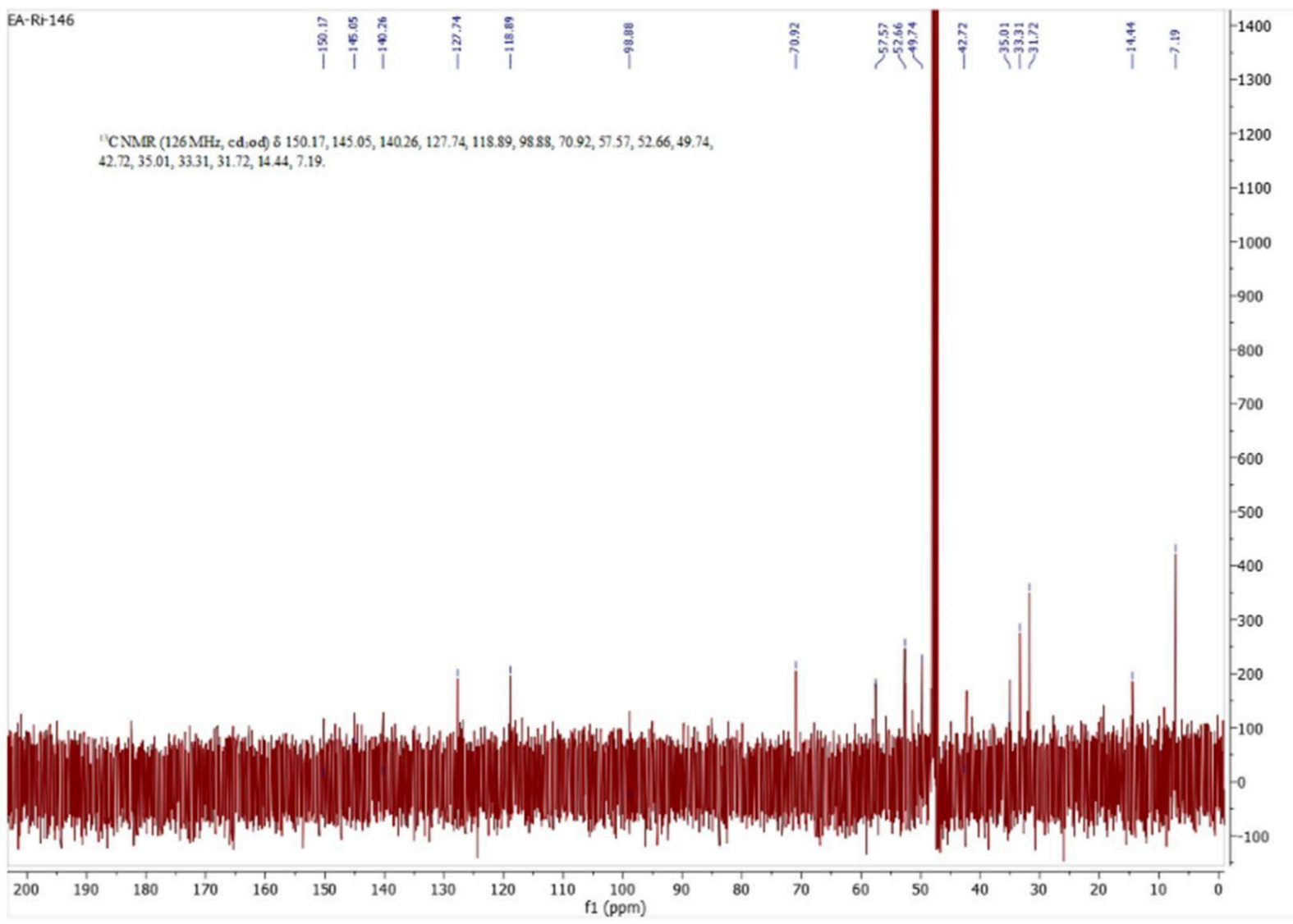

HRMS of EA-Ri-146:

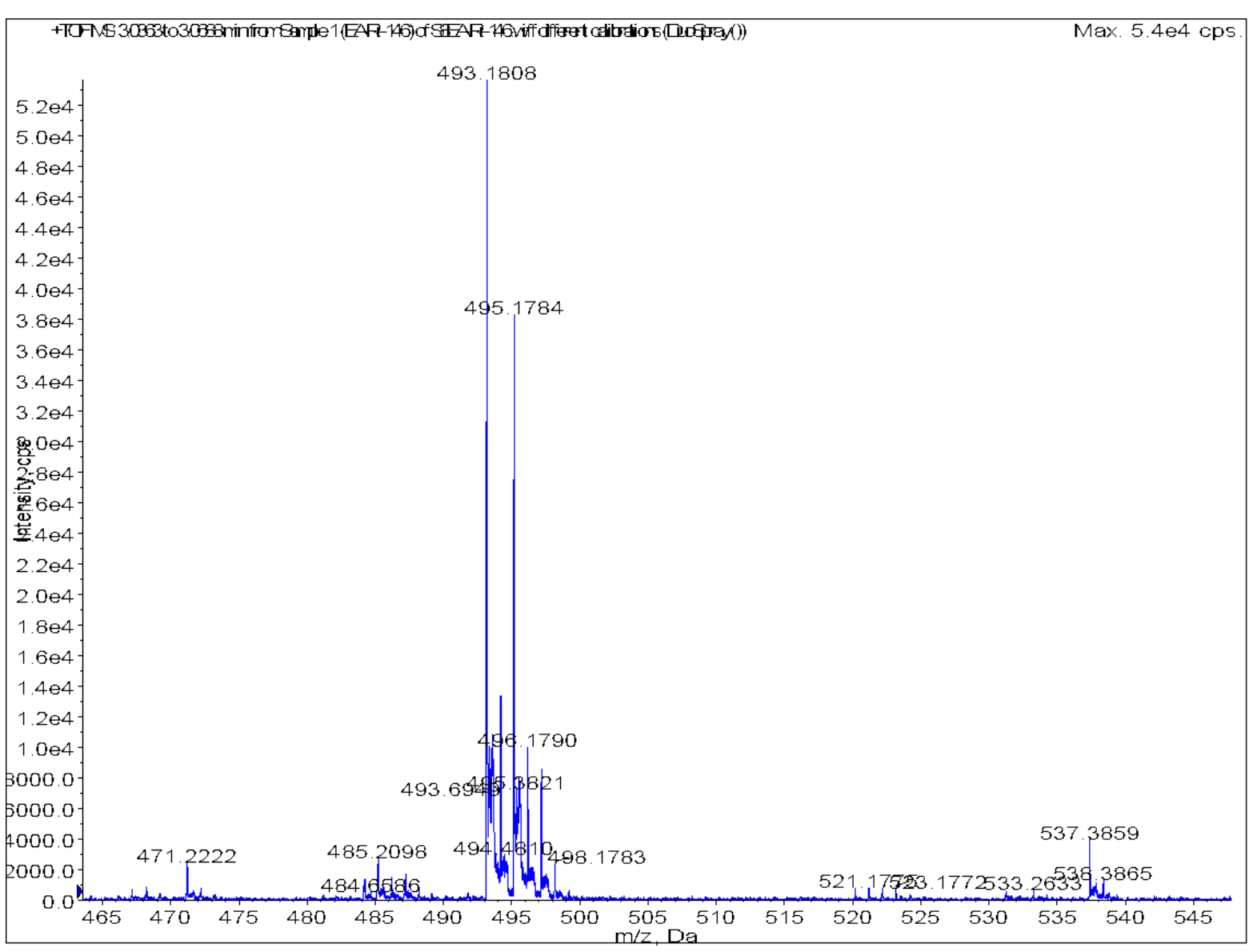

Formula	Calculated mass	Error / mDa	Error / ppm	DBE
C21 H35 N4 O3 S Cl2	493.1801	0.6549	1.328	5.5

EA-Ri-153 structure:

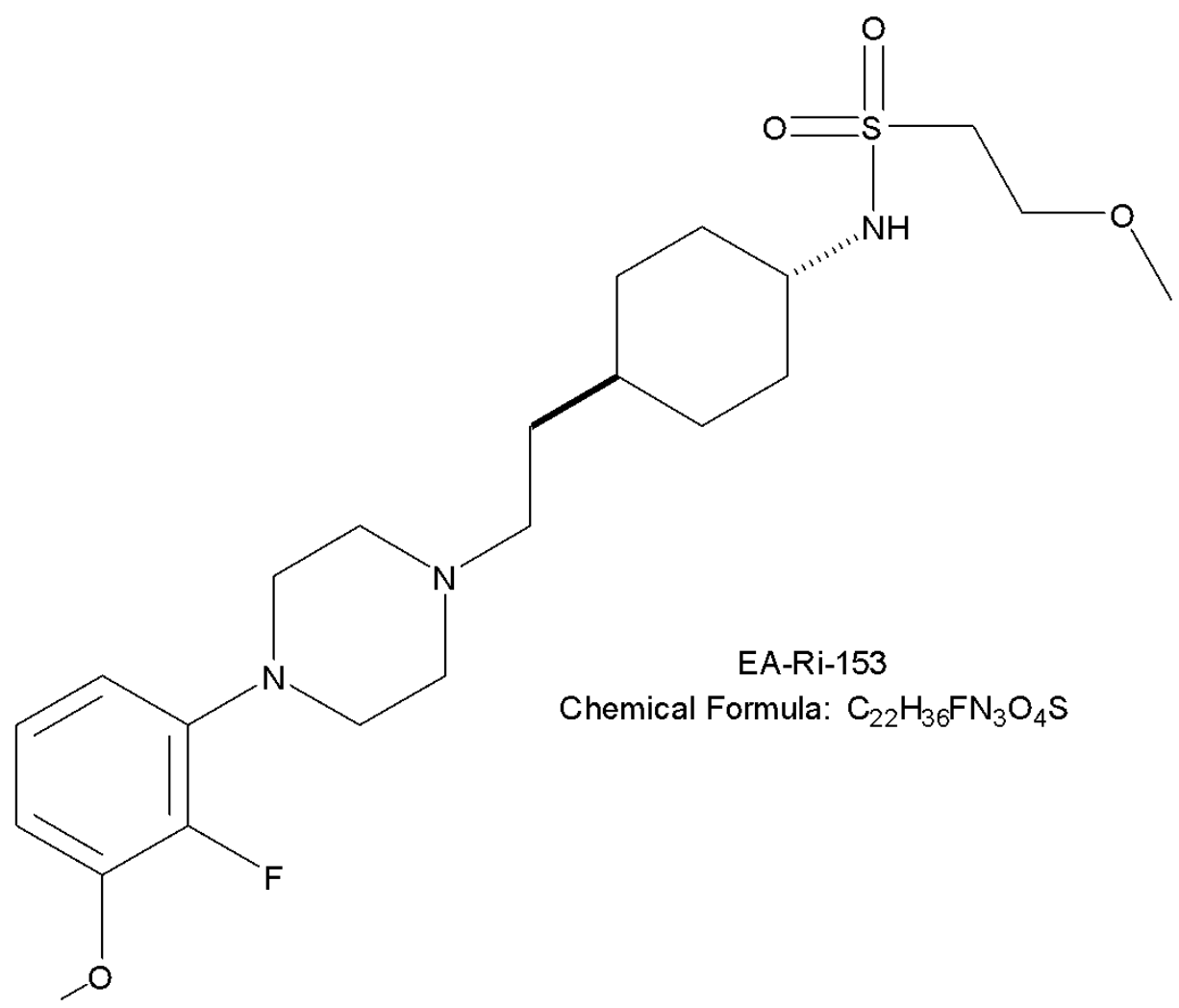

EA-Ri-153
Chemical Formula: $C_{22}H_{36}FN_3O_4S$

^{1}H NMR spectrum of EA-Ri-153:

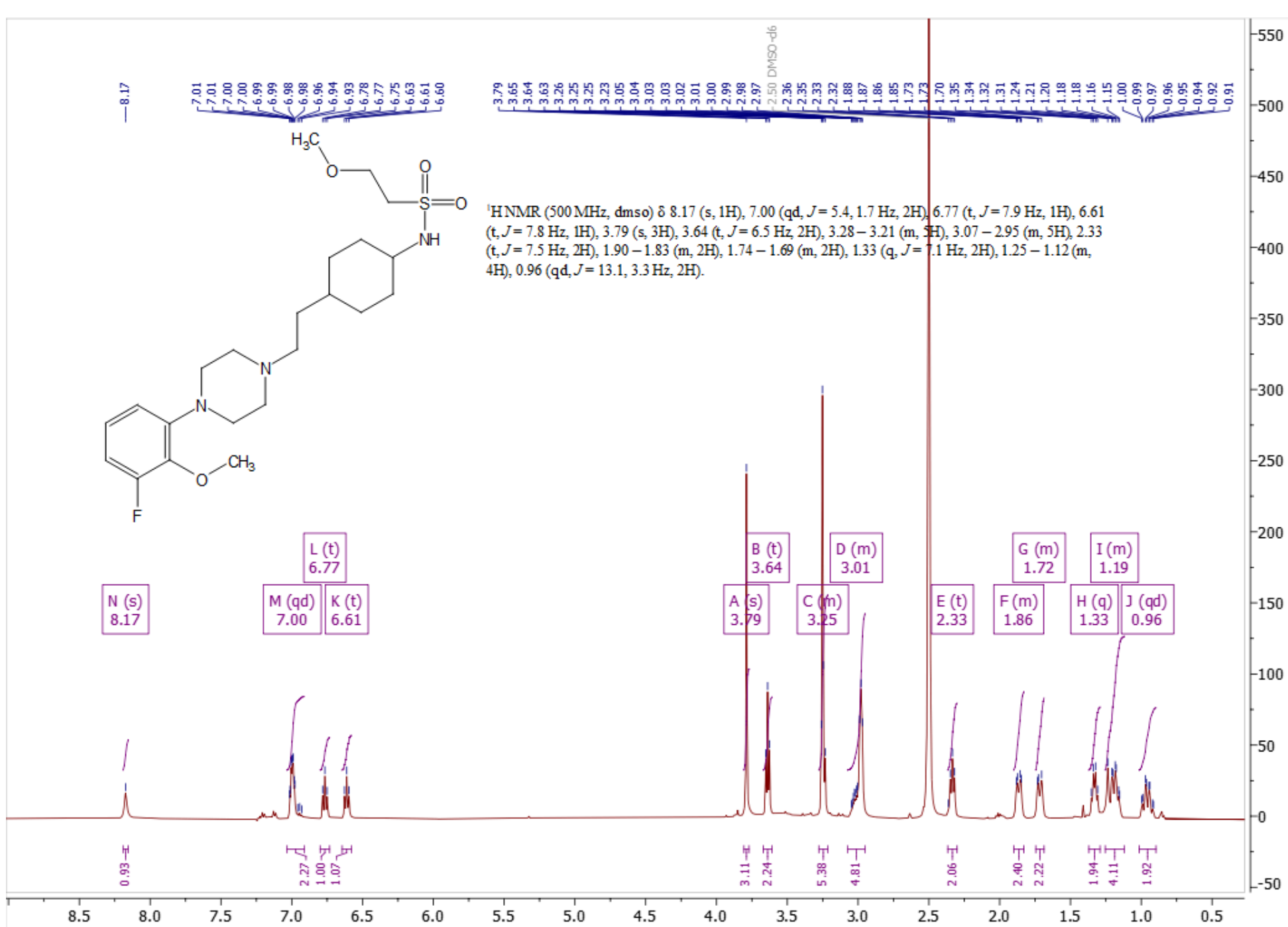

^{13}C NMR spectrum of EA-Ri-153:

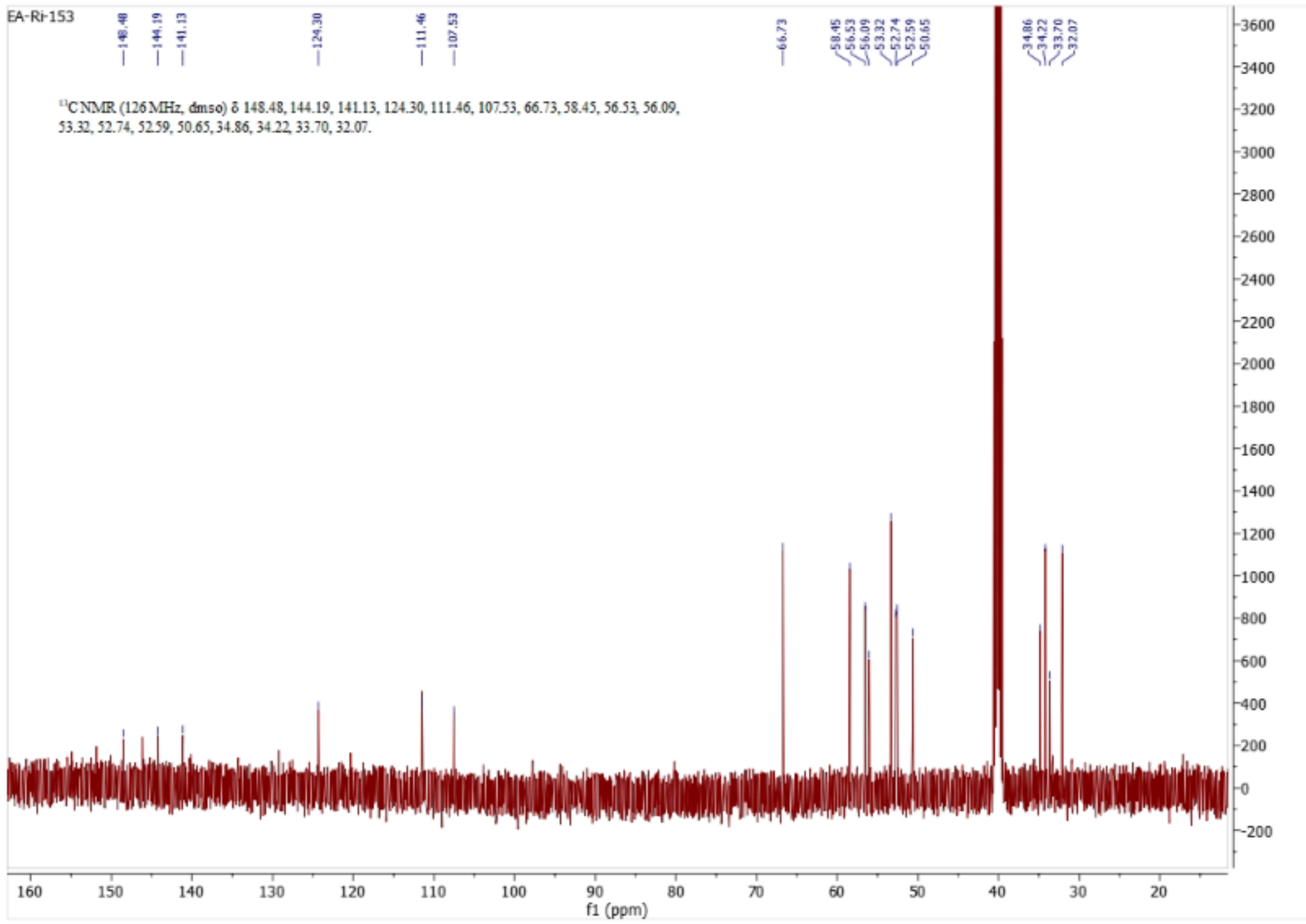

HRMS of EA-Ri-153:

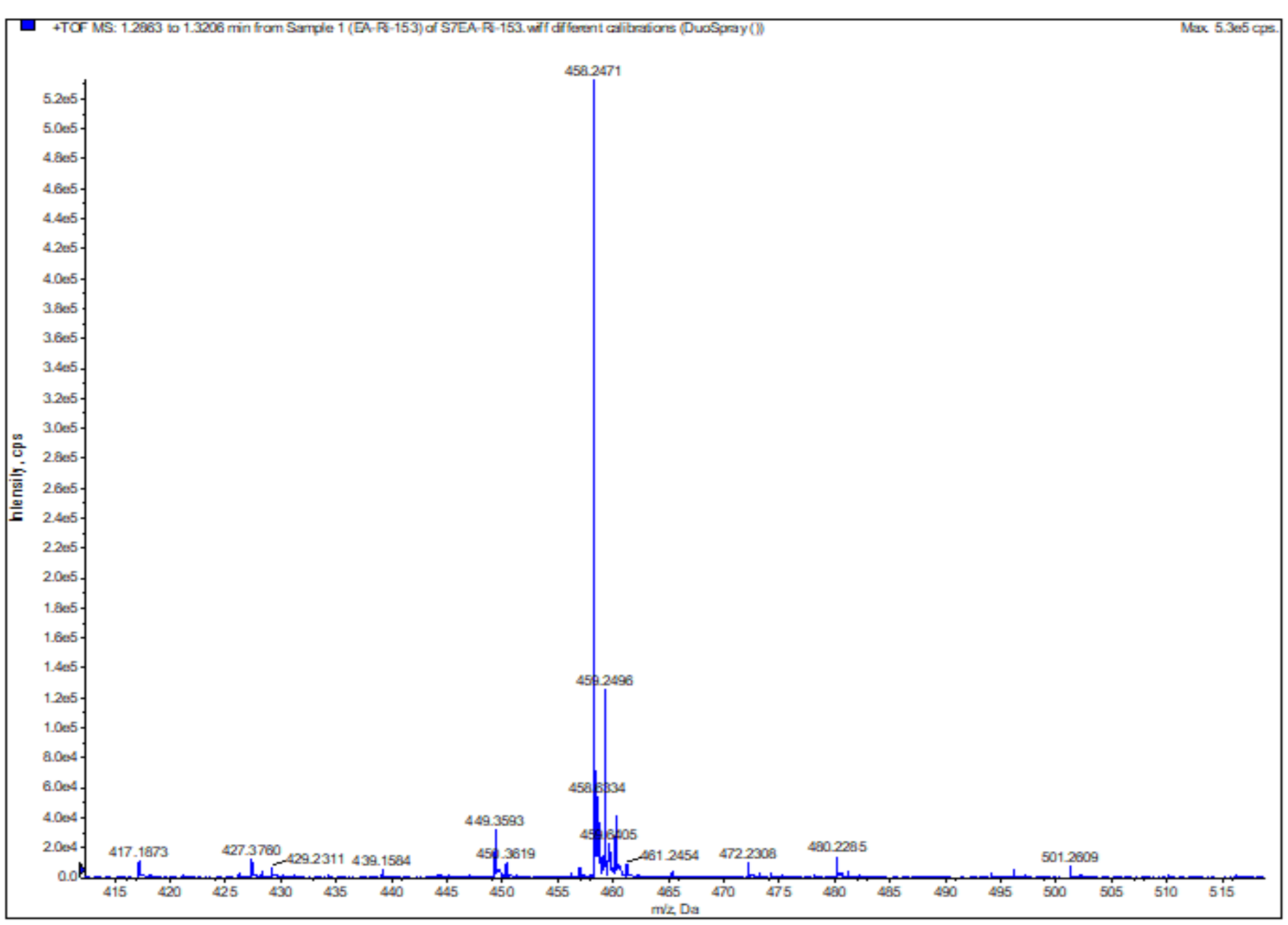

Formula	Calculated mass	Error / mDa	Error / ppm	DBE
C22 H37 N3 O4 F S	458.2483	-1.2335	-2.6918	5.5

Fluo2Car Intermediate 1 structure:

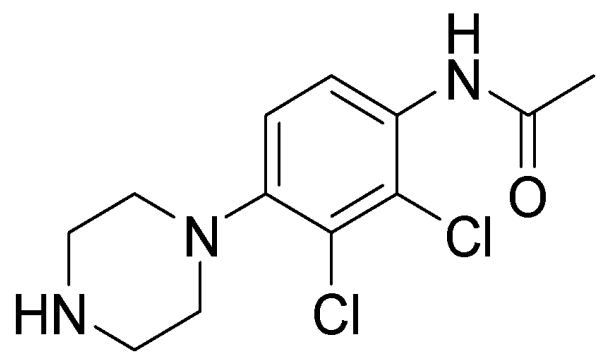

Int 1.

Chemical Formula: $C_{12}H_{15}Cl_2N_3O$

Exact Mass: 287.0592

^{1}H NMR spectrum of Fluo2Car Intermediate 1:

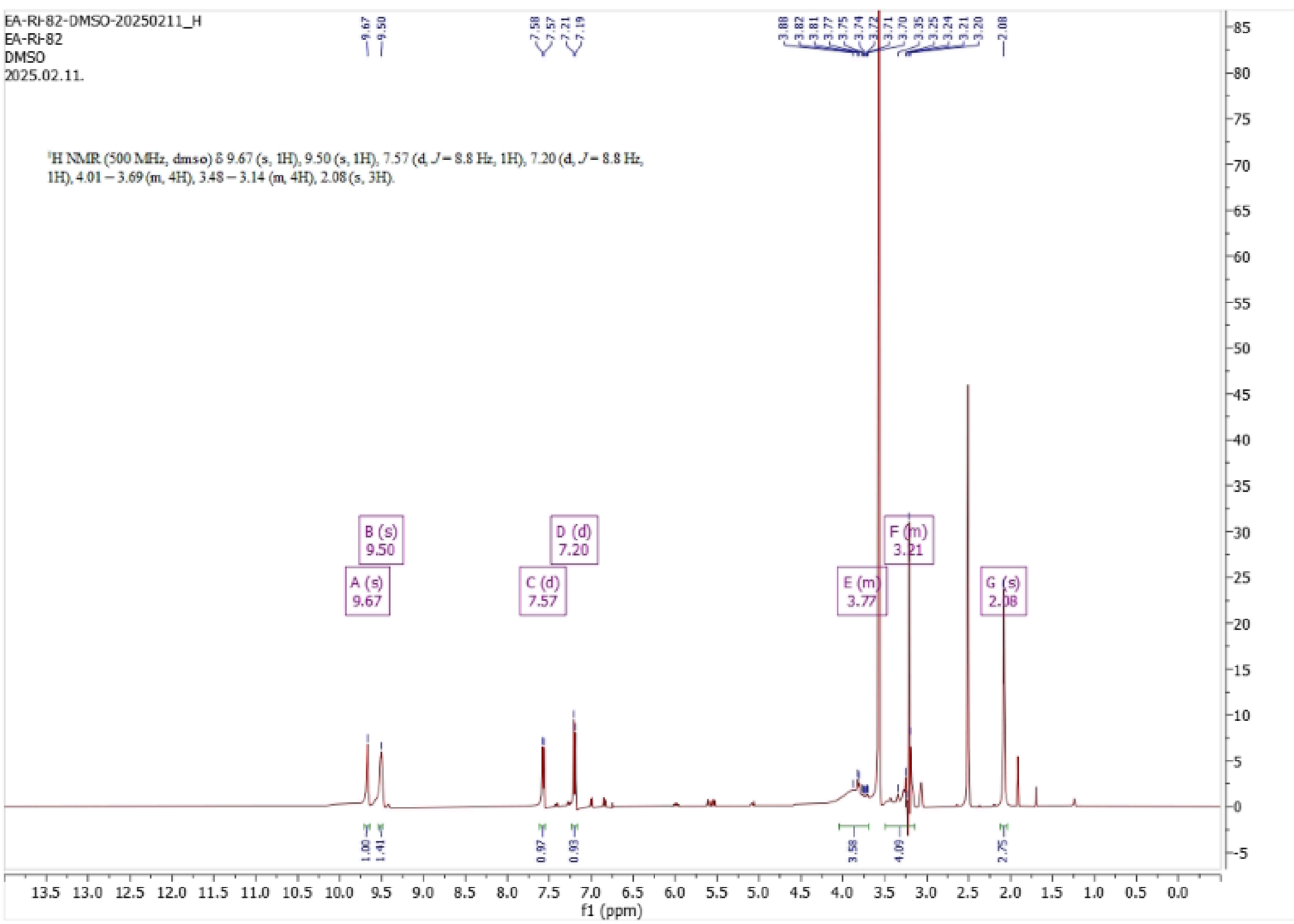

^{13}C NMR spectrum of Fluo2Car Intermediate 1:

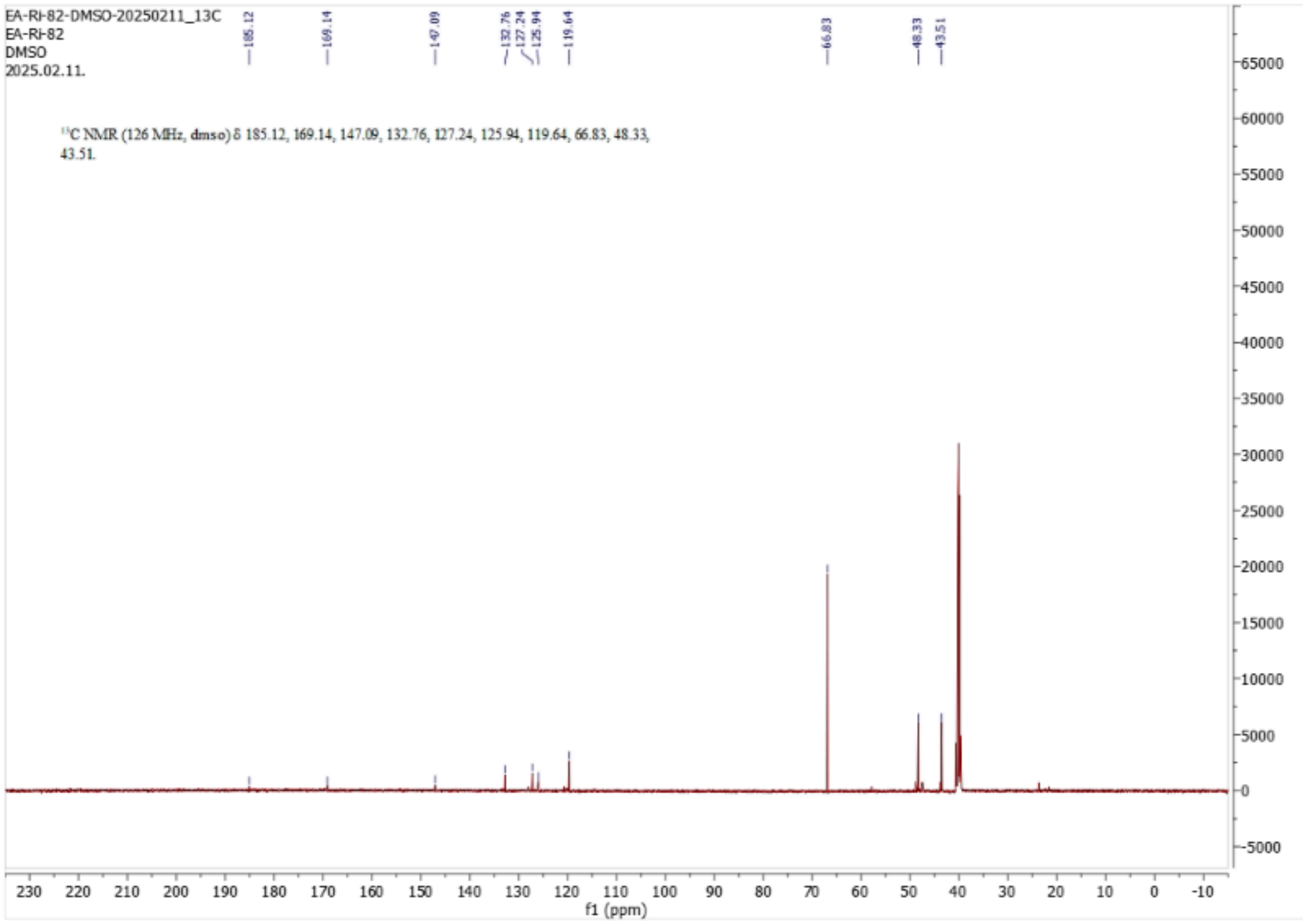

HRMS of Fluo2Car Intermediate 1:

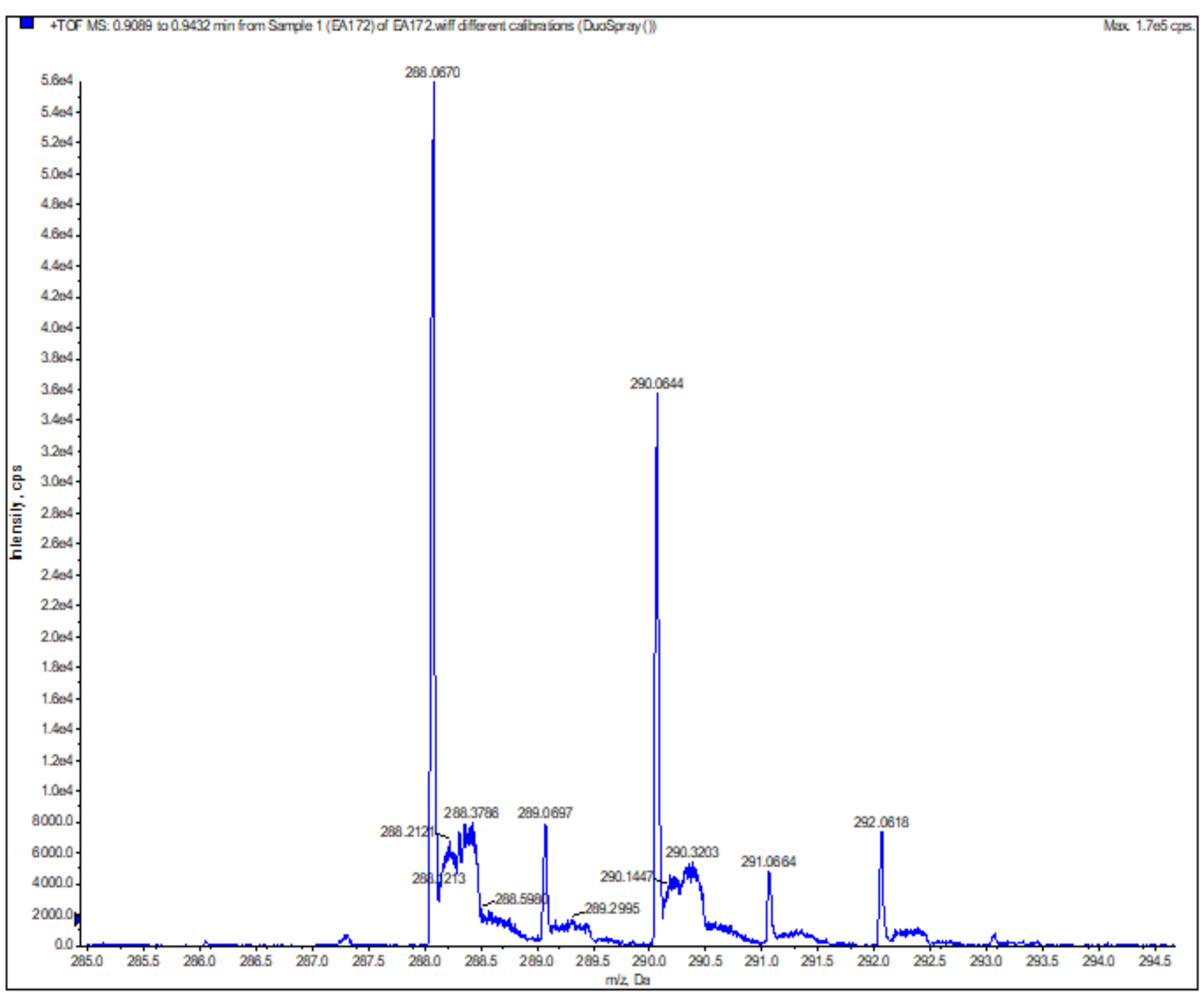

Formula	Calculated mass	Error / mDa	Error / ppm	DBE
C12 H16 N3 O Cl2	288.067	-0.0427	-0.1484	5.5

Fluo2Car Intermediate 2 structure:

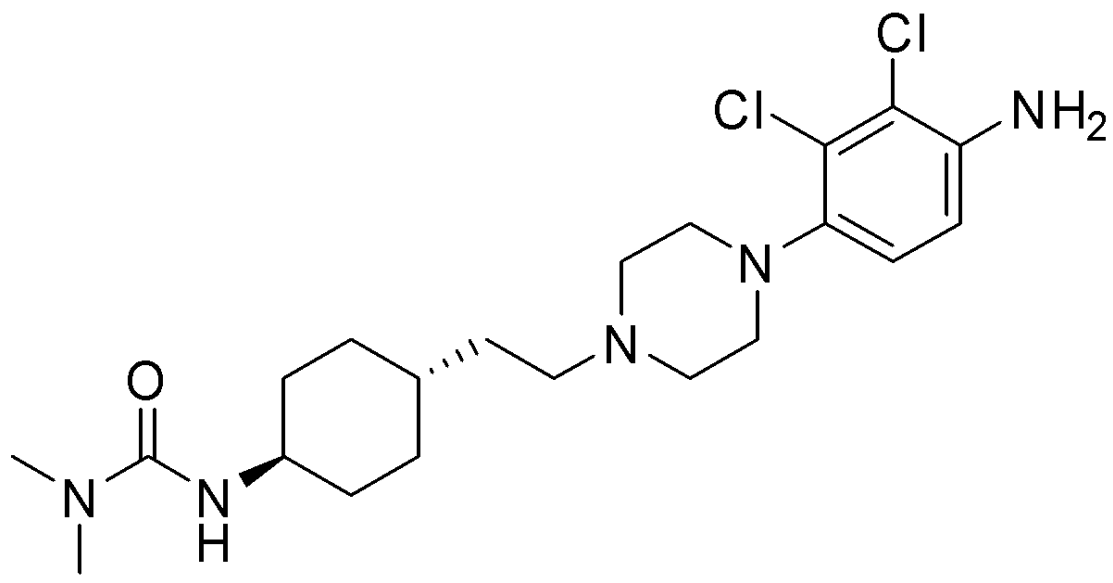

Int 2.
Chemical Formula: $C_{21}H_{33}Cl_2N_5O$

^{1}H NMR spectrum of Fluo2Car Intermediate 2:

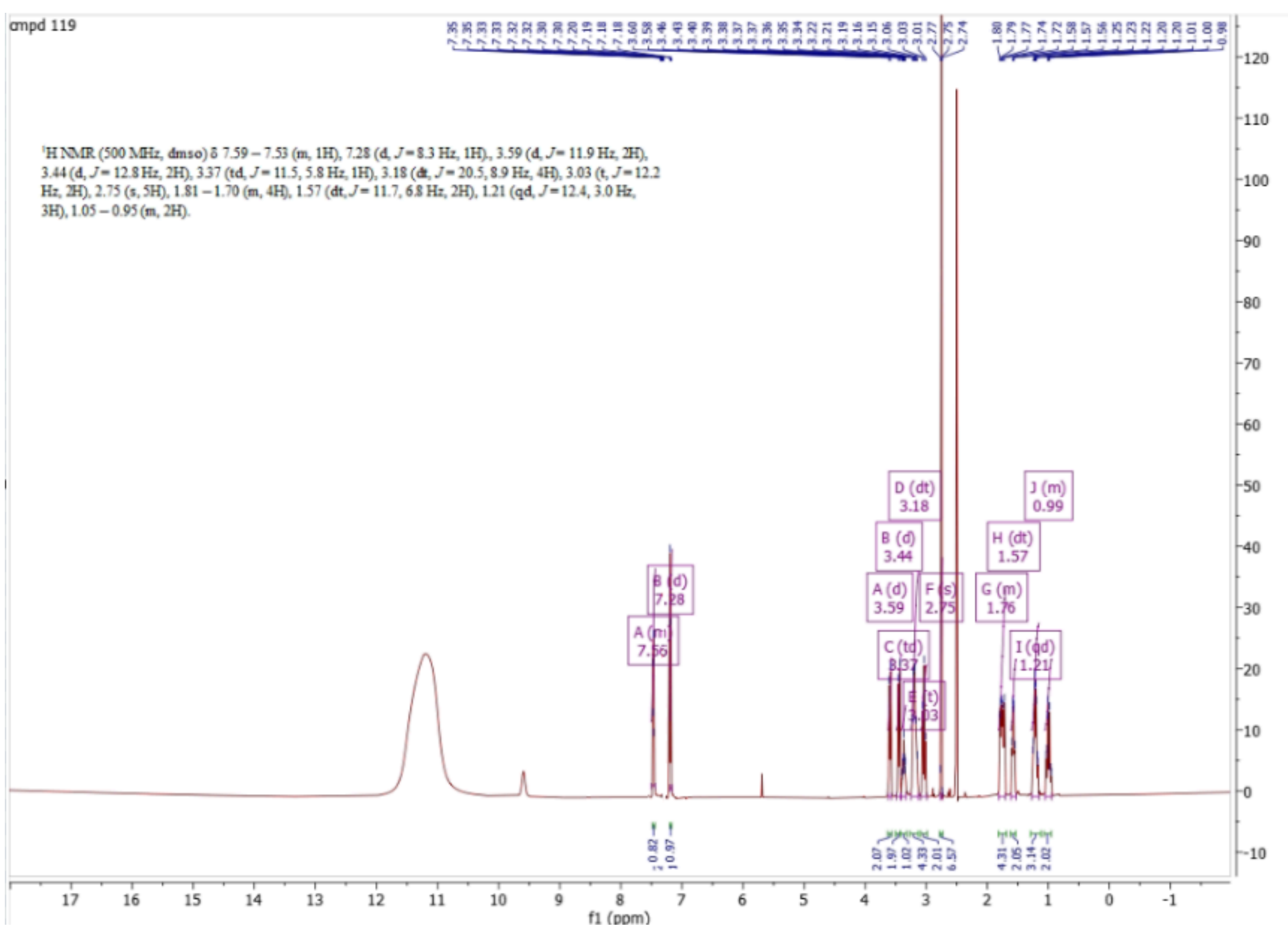

^{13}C NMR spectrum of Fluo2Car Intermediate 2:

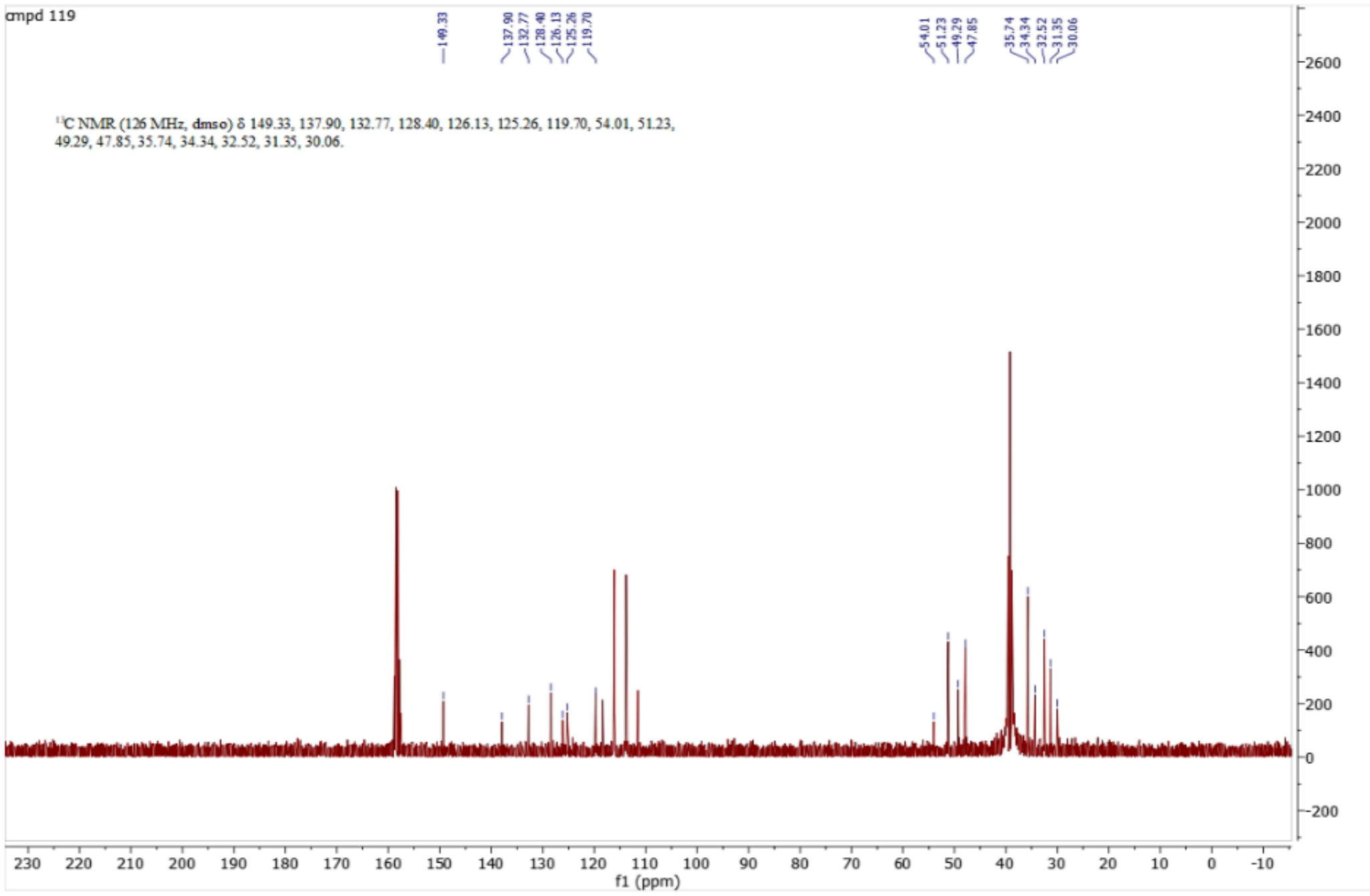

HRMS of Fluo2Car Intermediate 2:

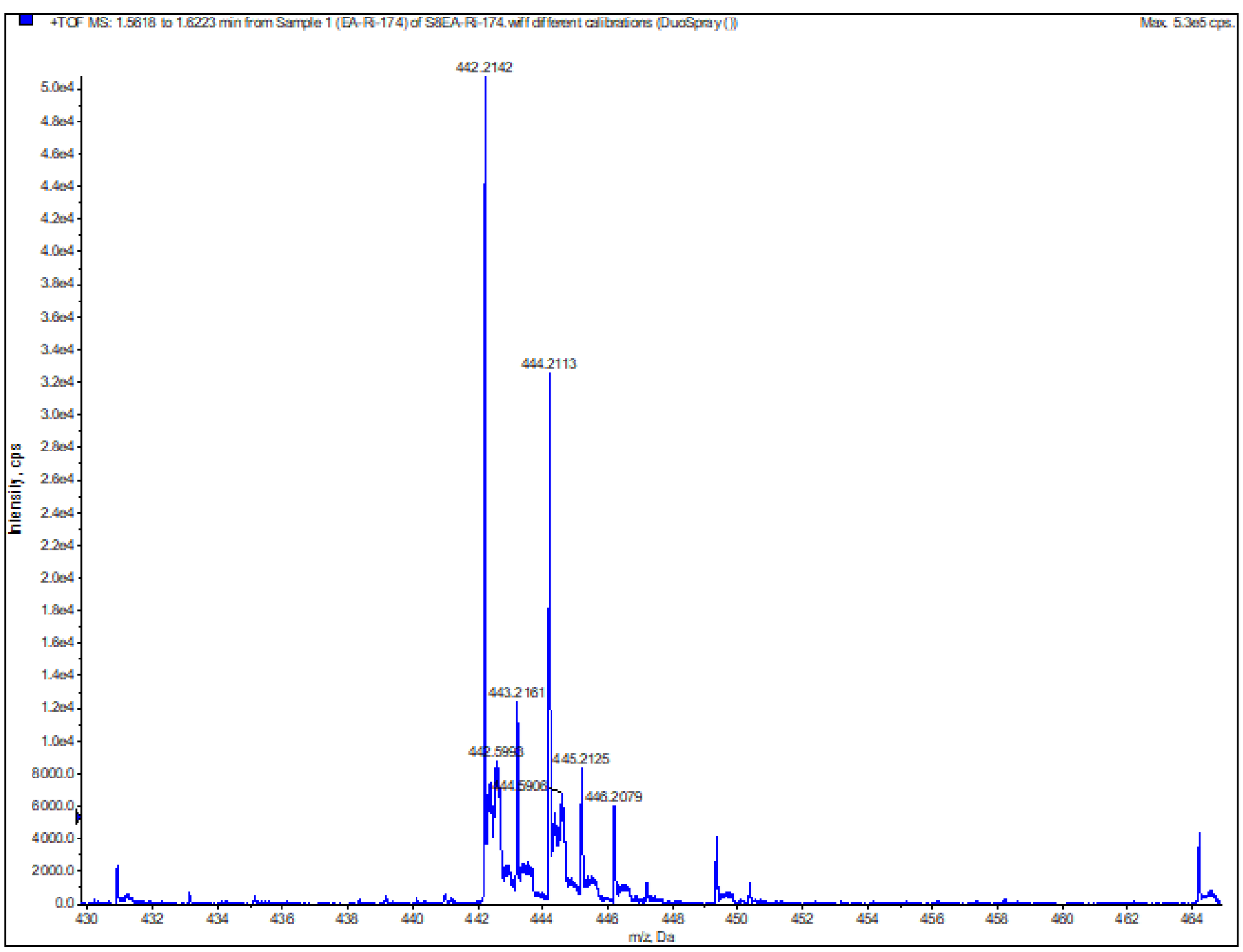

Formula	Calculated mass	Error / mDa	Error / ppm	DBE
C21 H34 N5 O Cl2	442.2134	0.707	1.5989	6.5

Fluo2Car structure:

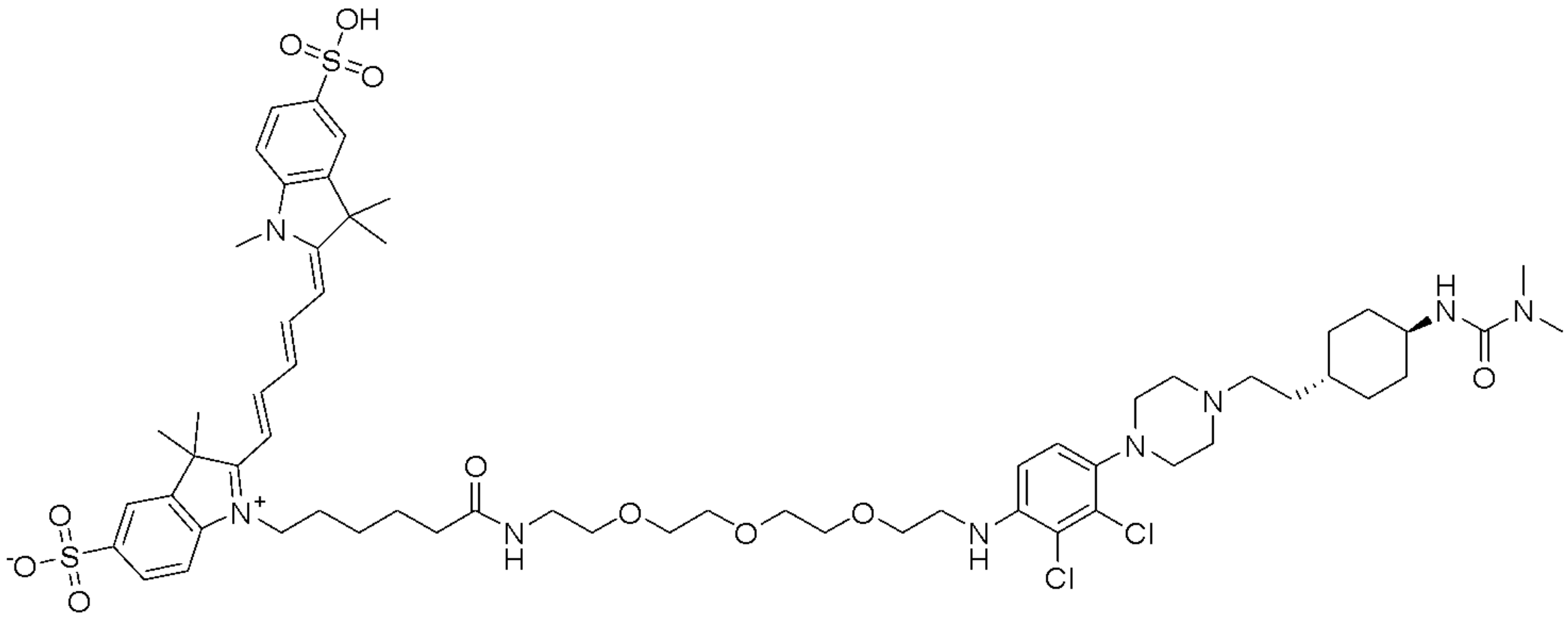

FluoCar2
Chemical Formula: $C_{61}H_{86}Cl_2N_8O_{11}S_2$

^{1}H NMR spectrum of Fluo2Car

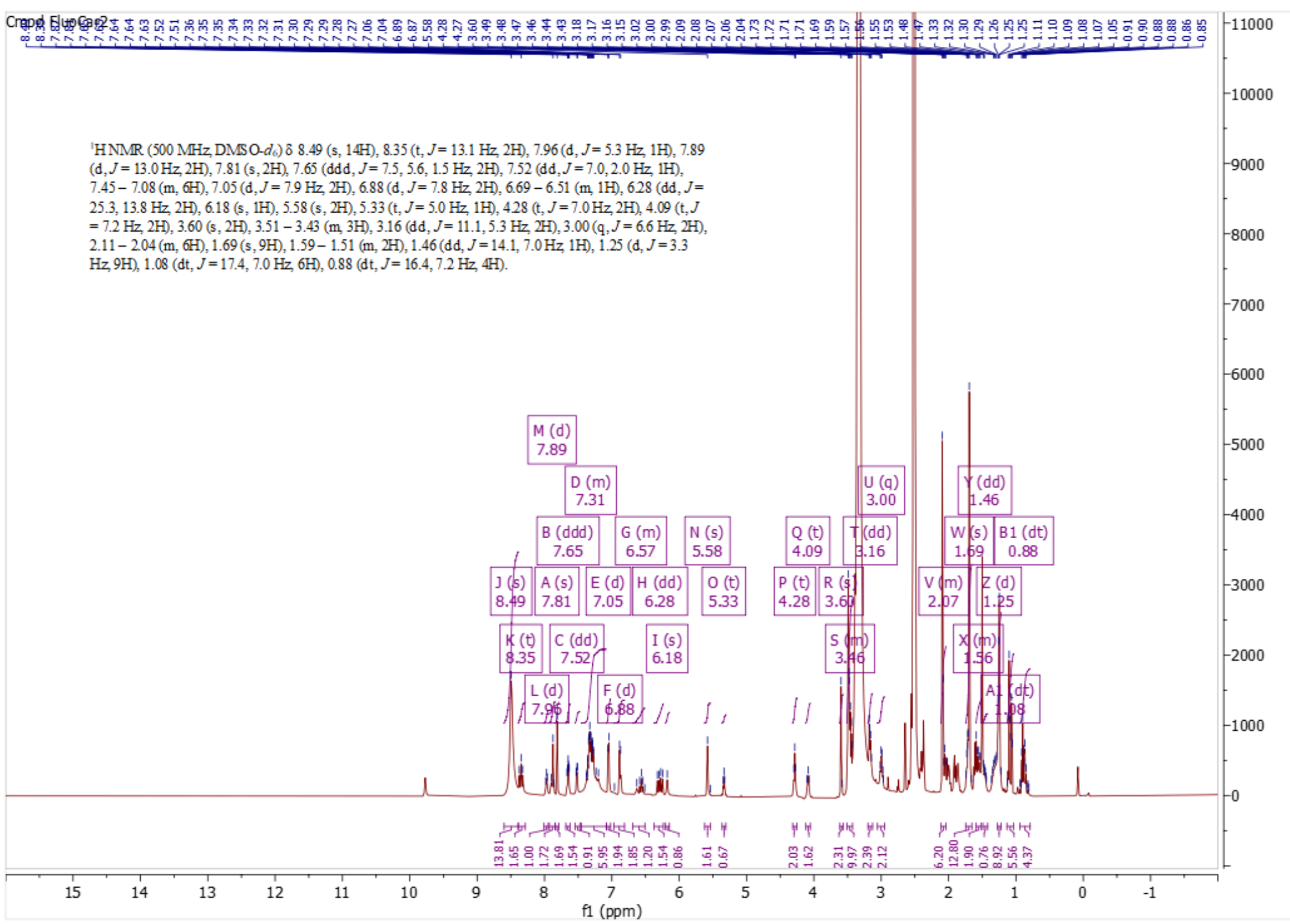

HRMS of Fluo2Car:

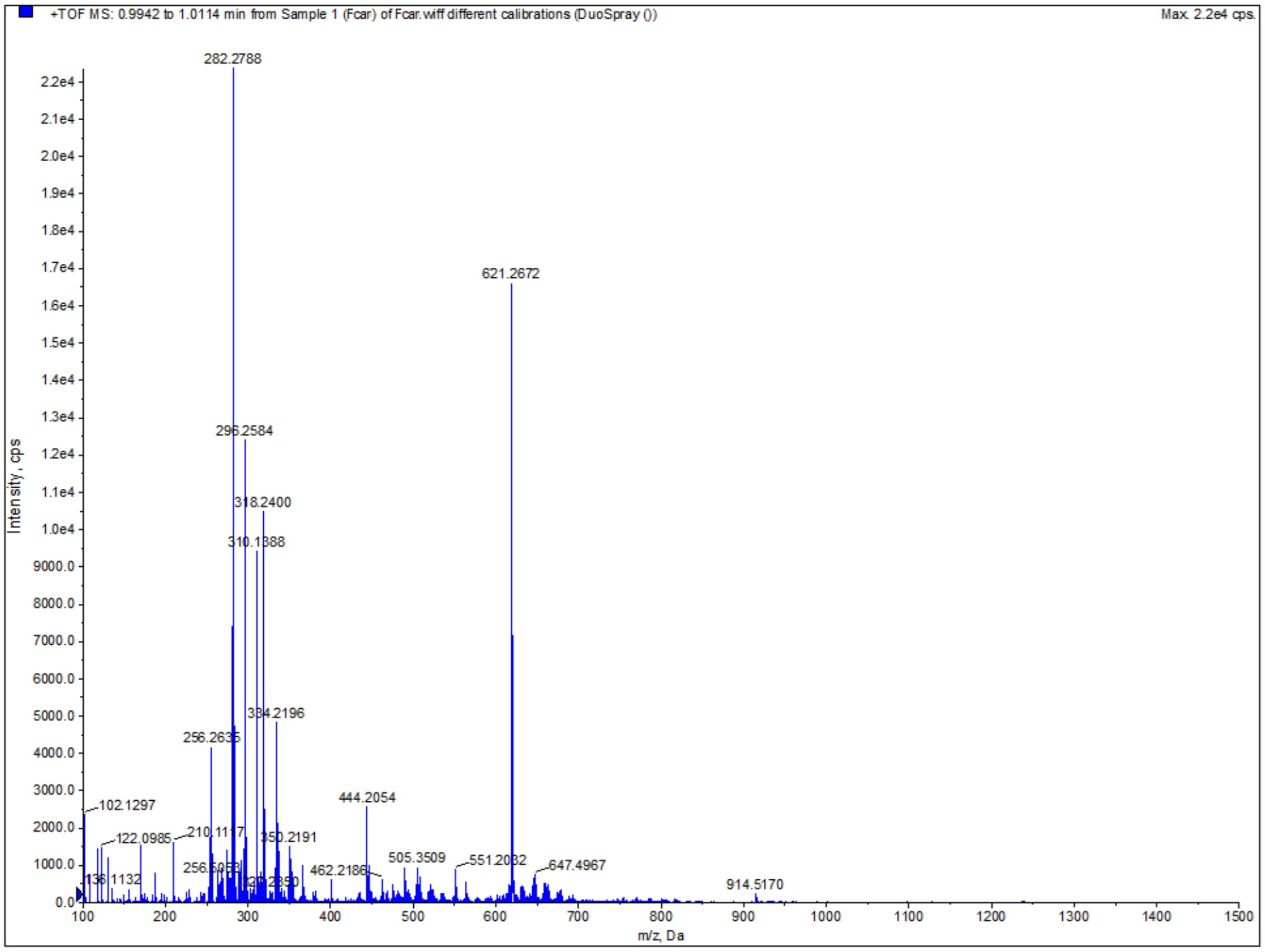

Formula	Calculated mass	Error / mDa	Error / ppm	DBE
C61 H88 N8 O11 S2 Cl2	621.2690	-1.8661	-3.0134	

HPLC purity of the tested compounds

EA-Ri-146

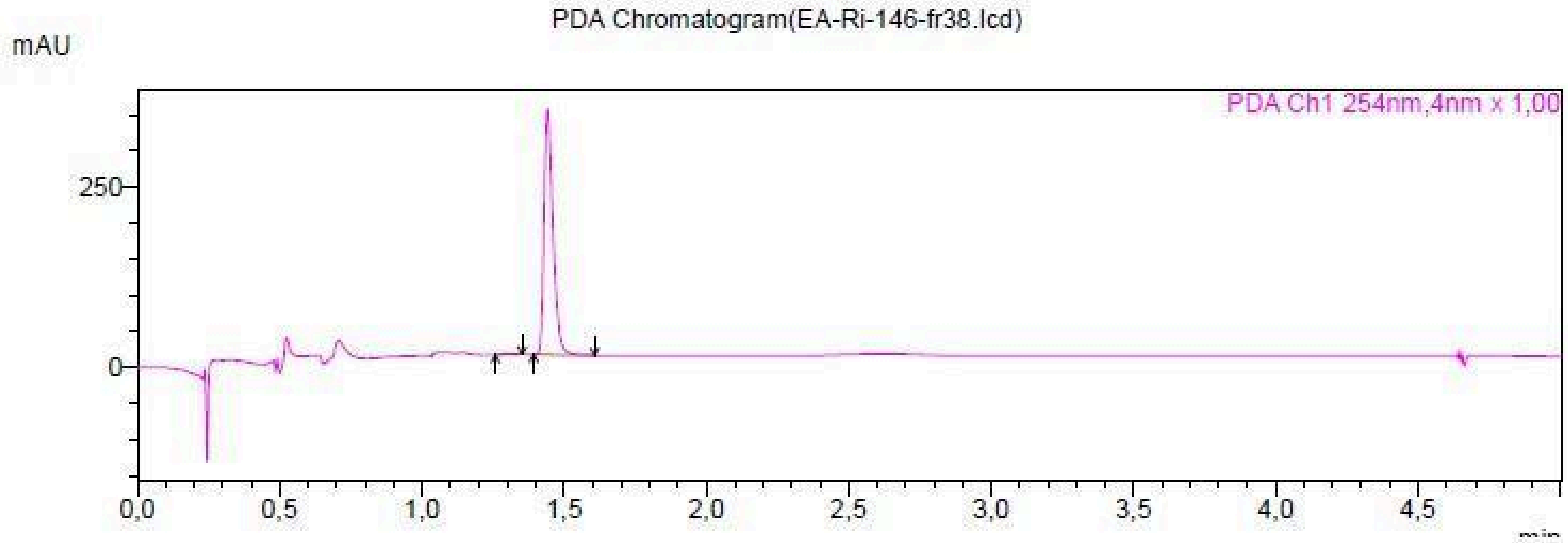

PDA Peak Table(EA-Ri-146-fr38.lcd)

PDA Ch1 254nm

Peak#	Ret. Time	Height	Area	Area%
1	1,298	1507	3333	0,443
2	1,442	329830	748251	99,557
Total		331337	751584	100,000

EA-Ri-153

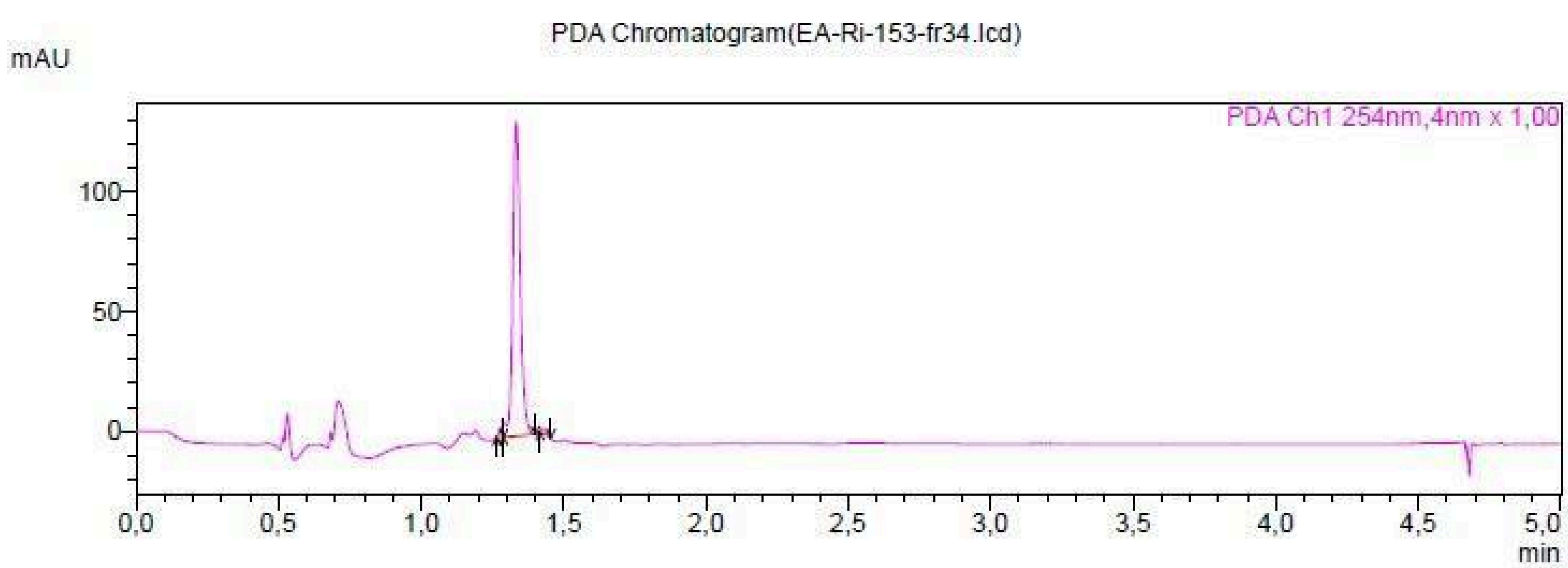

PDA Peak Table(EA-Ri-153-fr34.lcd)

PDA Ch1 254nm

Peak#	Ret. Time	Height	Area	Area%
1	1,281	2580	4066	1,671
2	1,333	128154	236598	97,225
3	1,427	2056	2686	1,104
Total		132790	243350	100,000

Cariprazine

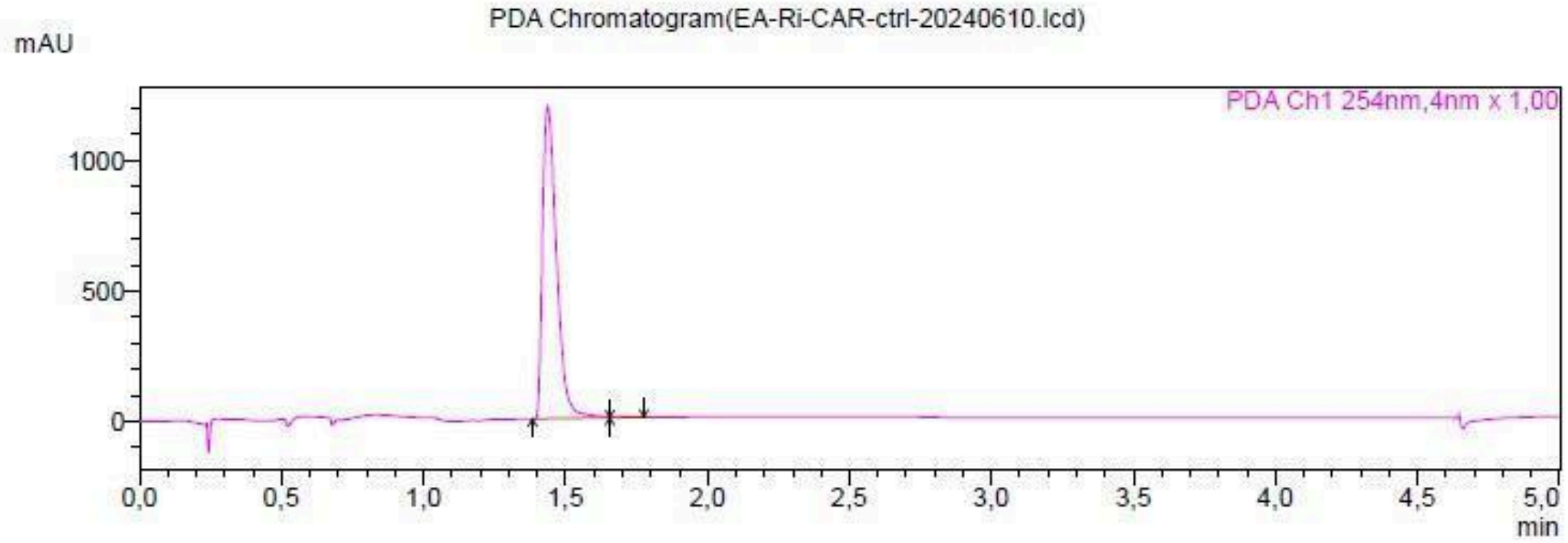

PDA Peak Table(EA-Ri-CAR-ctrl-20240610.lcd)

PDA Ch1 254nm

Peak#	Ret. Time	Height	Area	Area%
1	1.438	1188607	4124785	99.615
2	1.665	4718	15939	0.385
Total		1193325	4140723	100.000

EA-Ri-112

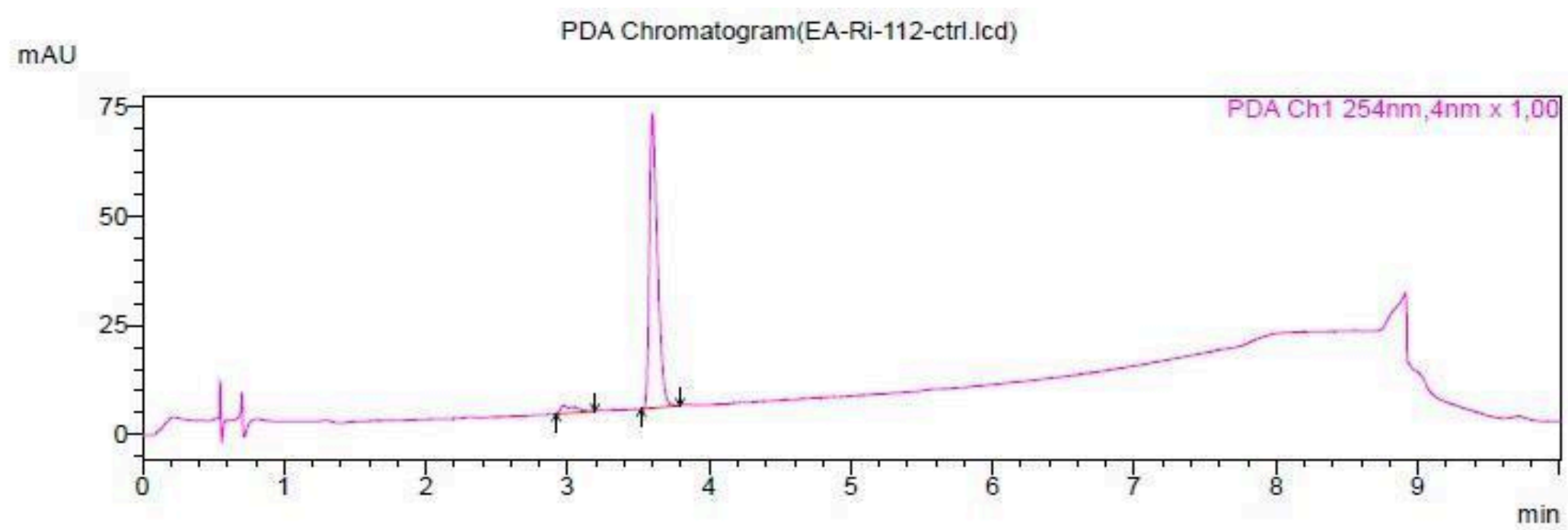

PDA Peak Table(EA-Ri-112-ctrl.lcd)

PDA Ch1 254nm

Peak#	Ret. Time	Height	Area	Area%
1	2.972	1726	12269	4.425
2	3.599	66572	265010	95.575
Total		68299	277279	100.000

EA-RK-110

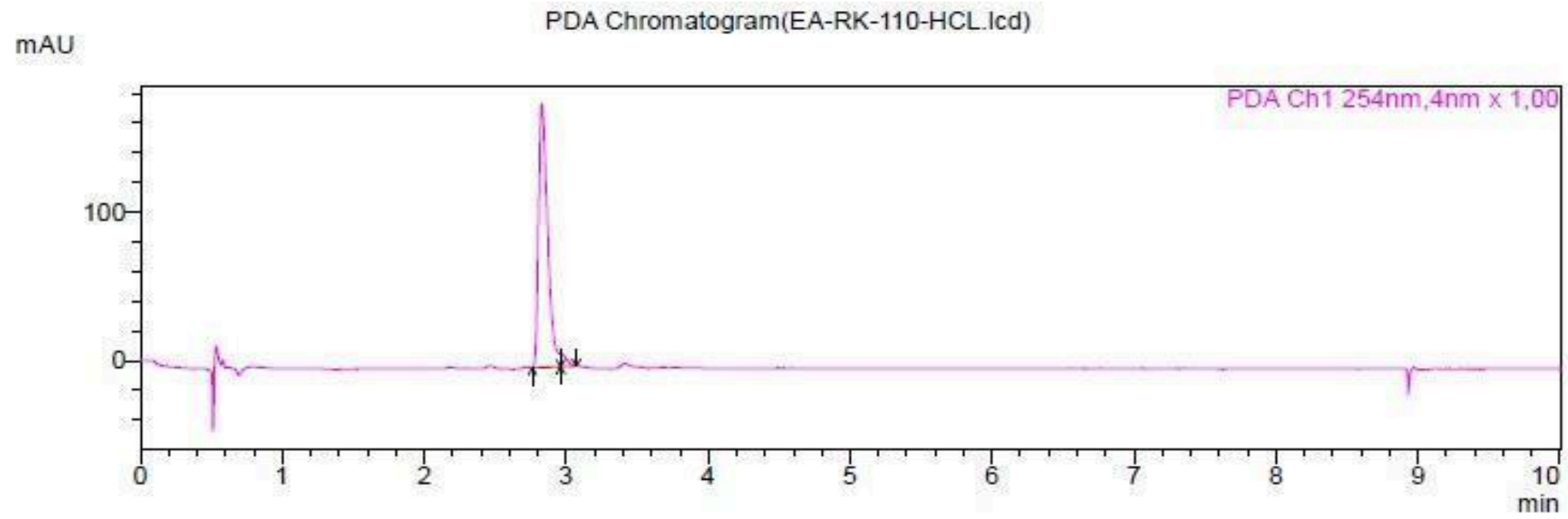

PDA Peak Table(EA-RK-110-HCL.lcd)

PDA Ch1 254nm

Peak#	Ret. Time	Height	Area	Area%
1	2.829	176740	791829	96.925
2	2.961	9101	25119	3.075
Total		185841	816948	100.000

FluoCar

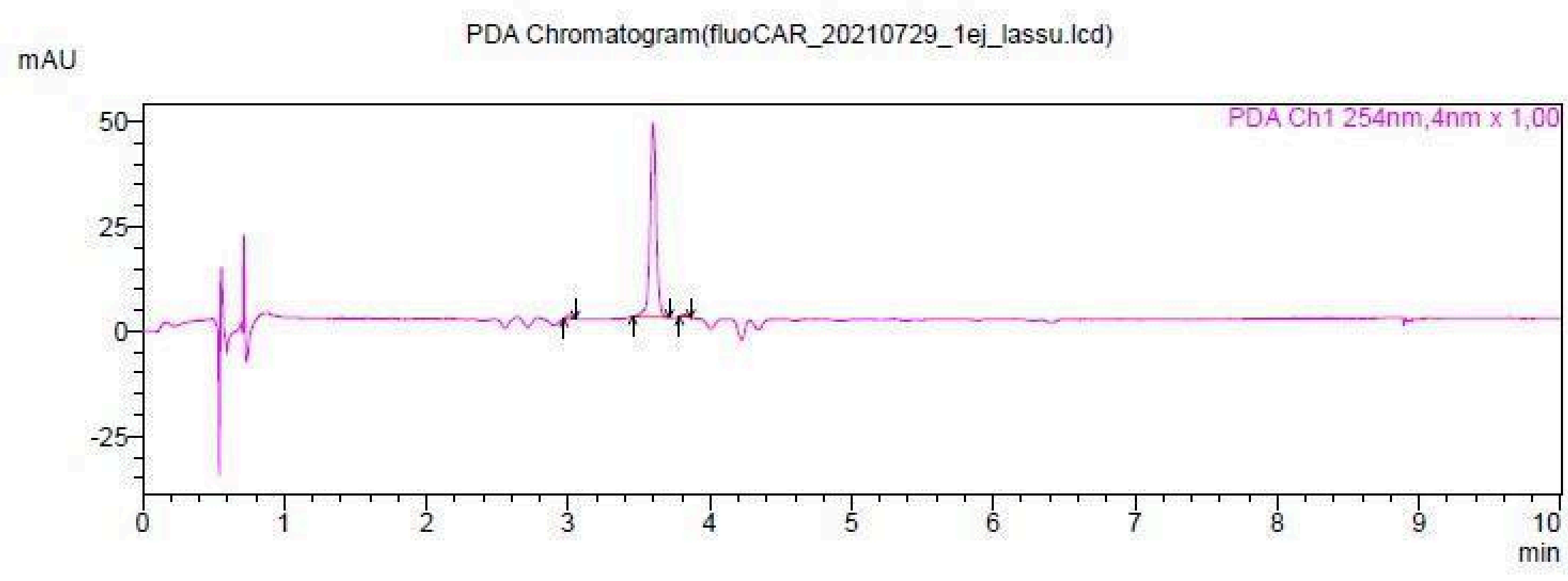

PDA Peak Table(fluoCAR_20210729_1ej_lassu.lcd)

PDA Ch1 254nm

Peak#	Ret. Time	Height	Area	Area%
1	3.004	906	2256	1.541
2	3.599	45489	142211	97.112
3	3.823	713	1972	1.347
Total		47107	146440	100.000

FluoCar2

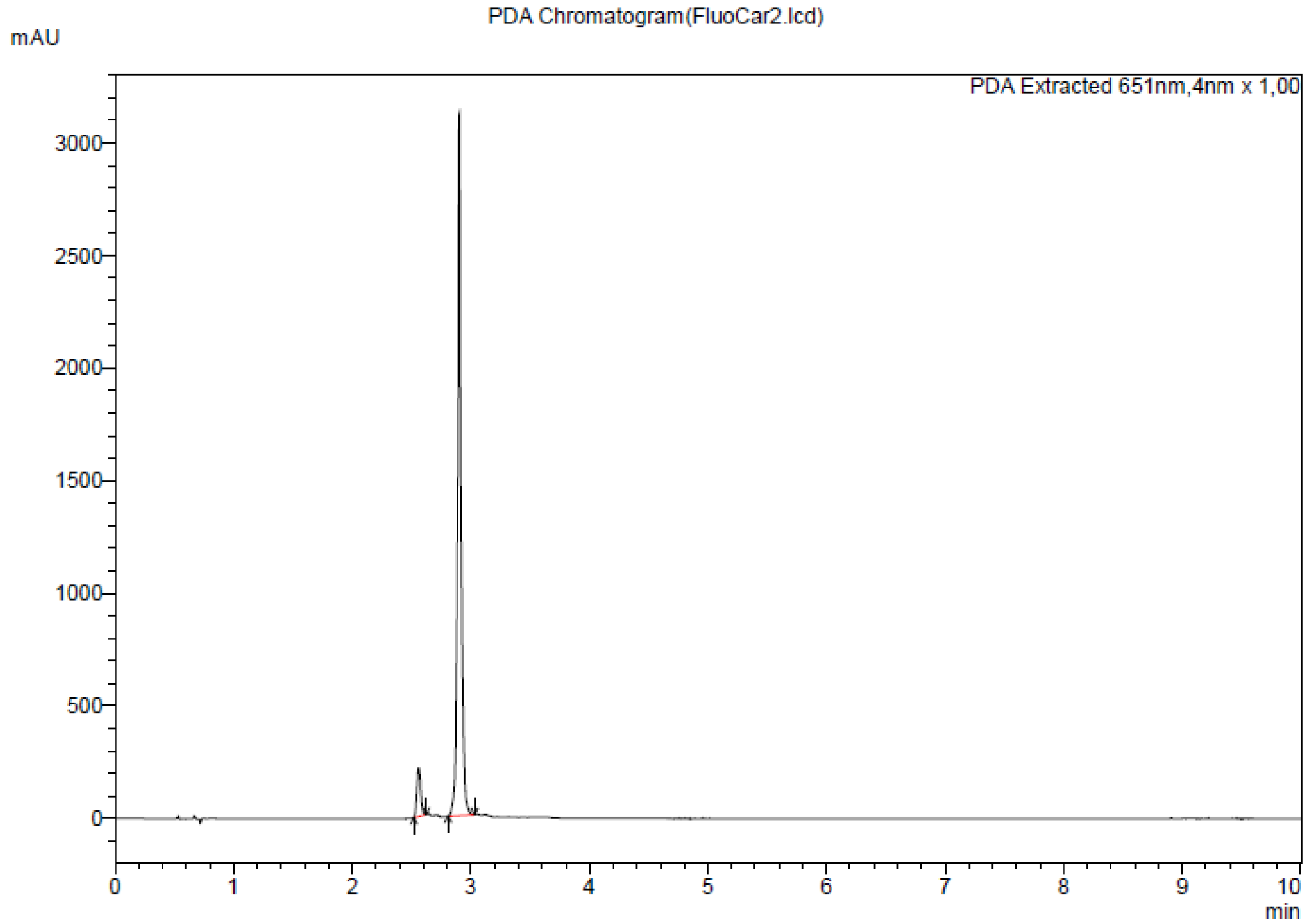

PDA Peak Table(FluoCar2.lcd)

PDA Extracted 651nm

Peak#	Ret. Time	Height	Area	Area%
1	2,561	208022	469538	6,793
2	2,903	2970217	6442079	93,207
Total		3178239	6911617	100,000

REFERENCES

1. J.-M. Beaulieu, R. R. Gainetdinov, The physiology, signaling, and pharmacology of dopamine receptors. *Pharmacol. Rev.* **63**, 182–217 (2011).

2. B. Landwehrmeyer, G. Mengod, J. M. Palacios, Dopamine D_3 receptor mRNA and binding sites in human brain. *Brain Res. Mol. Brain Res.* **18**, 187–192 (1993).

3. E. R. Larson, M. A. Ariano, D3 and D2 dopamine receptors: Visualization of cellular expression patterns in motor and limbic structures. *Synapse* **20**, 325–337 (1995).

4. J. H. Meador-Woodruff, S. P. Damask, J. Wang, V. Haroutunian, K. L. Davis, S. J. Watson, Dopamine receptor mRNA expression in human striatum and neocortex. *Neuropsychopharmacology* **15**, 17–29 (1996).

5. Z. U. Khan, A. Gutiérrez, R. Martín, A. Peñafiel, A. Rivera, A. De La Calle, Differential regional and cellular distribution of dopamine D2-like receptors: An immunocytochemical study of subtype-specific antibodies in rat and human brain. *J. Comp. Neurol.* **402**, 353–371 (1998).

6. M. Suzuki, Y. L. Hurd, P. Sokoloff, J. C. Schwartz, G. Sedvall, D_3 dopamine receptor mRNA is widely expressed in the human brain. *Brain Res.* **779**, 58–74 (1998).

7. E. V. Gurevich, J. N. Joyce, Distribution of dopamine D_3 receptor expressing neurons in the human forebrain: Comparison with D_2 receptor expressing neurons. *Neuropsychopharmacology* **20**, 60–80 (1999).

8. G. Searle, J. D. Beaver, R. A. Comley, M. Bani, A. Tziortzi, M. Slifstein, M. Mugnaini, C. Griffante, A. A. Wilson, E. Merlo-Pich, S. Houle, R. Gunn, E. A. Rabiner, M. Laruelle, Imaging dopamine D_3 receptors in the human brain with positron emission tomography, [^{11}C] PHNO, and a selective D_3 receptor antagonist. *Biol. Psychiatry* **68**, 392–399 (2010).

9. S. Prokop, P. Ábrányi-Balogh, B. Barti, M. Vámosi, M. Zöldi, L. Barna, G. M. Urbán, A. D. Tóth, B. Dudok, A. Egyed, H. Deng, G. M. Leggio, L. Hunyady, M. van der Stelt, G. M.

Keserű, I. Katona, PharmacoSTORM nanoscale pharmacology reveals cariprazine binding on Islands of Calleja granule cells. *Nat. Commun.* **12**, 6505 (2021).

10. H. Hall, C. Halldin, D. Dijkstra, H. Wikström, L. D. Wise, T. A. Pugsley, P. Sokoloff, S. Pauli, L. Farde, G. Sedvall, Autoradiographic localisation of D3-dopamine receptors in the human brain using the selective D3-dopamine receptor agonist (+)-[3H]PD 128907. *Psychopharmacology (Berl)* **128**, 240–247 (1996).

11. B. Kiss, I. Laszlovszky, B. Krámos, A. Visegrády, A. Bobok, G. Lévay, B. Lendvai, V. Román, Neuronal dopamine D3 receptors: Translational implications for preclinical research and CNS disorders. *Biomolecules* **11**, 104 (2021).

12. A. H. Newman, T. Ku, C. J. Jordan, A. Bonifazi, Z.-X. Xi, New drugs, old targets: Tweaking the dopamine system to treat psychostimulant use disorders. *Annu. Rev. Pharmacol. Toxicol.* **61**, 609–628 (2021).

13. E. V. Gurevich, Therapeutic applications dopamine D3 receptor function, new insight after 30 years research. *Curr. Top. Behav. Neurosci.* **60**, 29–45 (2022).

14.. Barabássy, Z. B. Dombi, G. Németh, D3 receptor-targeted cariprazine: Insights from lab to bedside. *Int. J. Mol. Sci.* **25**, 5682 (2024).

15. A. A. Bolonna, R. W. Kerwin, Partial agonism and schizophrenia. *Br. J. Psychiatry* **186**, 7–10 (2005).

16. J. A. Lieberman, Dopamine partial agonists: A new class of antipsychotic. *CNS Drugs* **18**, 251–267 (2004).

17. B. Kiss, A. Horváth, Z. Némethy, E. Schmidt, I. Laszlovszky, G. Bugovics, K. Fazekas, K. Hornok, S. Orosz, I. Gyertyán, E. Agai-Csongor, G. Domány, K. Tihanyi, N. Adham, Z. Szombathelyi, Cariprazine (RGH-188), a dopamine D_3 receptor-preferring, D_3/D_2 dopamine receptor antagonist-partial agonist antipsychotic candidate: In vitro and neurochemical profile. *J. Pharmacol. Exp. Ther.* **333**, 328–340 (2010).

18. S. Marder, W. W. Fleischhacker, W. Earley, K. Lu, Y. Zhong, G. Németh, I. Laszlovszky, E. Szalai, S. Durgam, Efficacy of cariprazine across symptom domains in patients with acute exacerbation of schizophrenia: Pooled analyses from 3 phase II/III studies. *Eur. Neuropsychopharmacol.* **29**, 127–136 (2019).

19. G. Németh, I. Laszlovszky, P. Czobor, E. Szalai, B. Szatmári, J. Harsányi, Á. Barabássy, M. Debelle, S. Durgam, I. Bitter, S. Marder, W. W. Fleischhacker, Cariprazine versus risperidone monotherapy for treatment of predominant negative symptoms in patients with schizophrenia: A randomised, double-blind, controlled trial. *Lancet* **389**, 1103–1113 (2017).

20. E. S. Gogarnoiu, C. D. Vogt, J. Sanchez, A. Bonifazi, E. Saab, A. B. Shaik, O. Soler-Cedeño, G.-H. Bi, B. Klein, Z.-X. Xi, J. R. Lane, A. H. Newman, Dopamine D_3/D_2 receptor ligands based on cariprazine for the treatment of psychostimulant use disorders that may be dual diagnosed with affective disorders. *J. Med. Chem.* **66**, 1809–1834 (2023).

21. A. Newman-Tancredi, M. S. Kleven, Comparative pharmacology of antipsychotics possessing combined dopamine D_2 and serotonin 5-HT_{1A} receptor properties. *Psychopharmacology* **216**, 451–473 (2011).

22. A. Do, K. Keramatian, A. Schaffer, L. Yatham, Cariprazine in the treatment of bipolar disorder: Within and beyond clinical trials. *Front. Psych.* **12**, 769897 (2021).

23. W. R. Earley, H. Guo, G. Németh, J. Harsányi, M. E. Thase, Cariprazine augmentation to antidepressant therapy in major depressive disorder: Results of a randomized, double-blind, placebo-controlled trial. *Psychopharmacol. Bull.* **48**, 62–80 (2018).

24. E. Vieta, W. R. Earley, M. V. Burgess, S. Durgam, C. Chen, Y. Zhong, Á. Barabássy, G. Németh, Long-term safety and tolerability of cariprazine as adjunctive therapy in major depressive disorder. *Int. Clin. Psychopharmacol.* **34**, 76–83 (2019).

25. S. Durgam, W. Earley, H. Guo, D. Li, G. Németh, I. Laszlovszky, M. Fava, S. A. Montgomery, Efficacy and safety of adjunctive cariprazine in inadequate responders to antidepressants: A randomized, double-blind, placebo-controlled study in adult patients with major depressive disorder. *J. Clin. Psychiatry* **77**, 371–378 (2016).

26. G. S. Sachs, P. P. Yeung, L. Rekeda, A. Khan, J. L. Adams, M. Fava, Adjunctive cariprazine for the treatment of patients with major depressive disorder: A randomized, double-blind, placebo-controlled phase 3 study. *Am. J. Psychiatry* **180**, 241–251 (2023).

27. M. Papp, P. Gruca, M. Lasoń-Tyburkiewicz, N. Adham, B. Kiss, I. Gyertyán, Attenuation of anhedonia by cariprazine in the chronic mild stress model of depression. *Behav. Pharmacol.* **25**, 567–574 (2014).

28. V. Duric, M. Banasr, T. Franklin, A. Lepack, N. Adham, B. Kiss, I. Gyertyán, R. S. Duman, Cariprazine exhibits anxiolytic and dopamine D_3 receptor-dependent antidepressant effects in the chronic stress model. *Int. J. Neuropsychopharmacol.* **20**, 788–796 (2017).

29. A. Herman, M. El Mansari, N. Adham, B. Kiss, B. Farkas, P. Blier, Involvement of 5-HT_{1A} and 5-HT_{2A} receptors but not α_2-adrenoceptors in the acute electrophysiological effects of cariprazine in the rat brain in vivo. *Mol. Pharmacol.* **94**, 1363–1370 (2018).

30. P. G. Strange, Antipsychotic drug action: Antagonism, inverse agonism or partial agonism. *Trends Pharmacol. Sci.* **29**, 314–321 (2008).

31. B. L. Roth, D. J. Sheffler, W. K. Kroeze, Magic shotguns versus magic bullets: Selectively non-selective drugs for mood disorders and schizophrenia. *Nat. Rev. Drug Discov.* **3**, 353–359 (2004).

32. A. Egyed, D. J. Kiss, G. M. Keserű, The impact of the secondary binding pocket on the pharmacology of class A GPCRs. *Front. Pharmacol.* **13**, 847788 (2022).

33. E. Y. T. Chien, W. Liu, Q. Zhao, V. Katritch, G. W. Han, M. A. Hanson, L. Shi, A. H. Newman, J. A. Javitch, V. Cherezov, R. C. Stevens, Structure of the human dopamine D3 receptor in complex with a D2/D3 selective antagonist. *Science* **330**, 1091–1095 (2010).

34. A. H. Newman, F. O. Battiti, A. Bonifazi, 2016 Philip S. Portoghese medicinal chemistry lectureship: Designing bivalent or bitopic molecules for G-protein coupled receptors. The whole is greater than the sum of its parts. *J. Med. Chem.* **63**, 1779–1797 (2020).

35. A. Egyed, D. J. Kiss, Z. Pável, K. Rajna, S. A. Gaitonde, M. Dubiel, M. I. Loza, E. Szűcs, É. Borbély, Z. Hajna, G. Satala, M. Vass, P. Szabó, K. Jósvay, A. J. Bojarski, H. Stark, Z. Helyes, E. Pintér, M. Bouvier, G. M. Keserű, The impact of secondary site interactions on the binding specificity of bitopic dopamine D_2/D_3 receptor ligands. *Eur. J. Med. Chem. Rep.* **15**, 100302 (2025).

36. M. Michino, P. Donthamsetti, T. Beuming, A. Banala, L. Duan, T. Roux, Y. Han, E. Trinquet, A. H. Newman, J. A. Javitch, L. Shi, A single glycine in extracellular loop 1 is the critical determinant for pharmacological specificity of dopamine D_2 and D_3 receptors. *Mol. Pharmacol.* **84**, 854–864 (2013).

37. A. E. Moritz, A. Bonifazi, A. M. Guerrero, V. Kumar, R. B. Free, J. R. Lane, R. K. Verma, L. Shi, A. H. Newman, D. R. Sibley, Evidence for a stereoselective mechanism for bitopic activity by extended-length antagonists of the D_3 dopamine receptor. *ACS Chem. Nerosci.* **11**, 3309–3320 (2020).

38. A. H. Newman, T. Beuming, A. K. Banala, P. Donthamsetti, K. Pongetti, A. LaBounty, B. Levy, J. Cao, M. Michino, R. R. Luedtke, J. A. Javitch, L. Shi, Molecular determinants of selectivity and efficacy at the dopamine D_3 receptor. *J. Med. Chem.* **55**, 6689–6699 (2012).

39. C. Klein Herenbrink, R. Verma, H. D. Lim, A. Kopinathan, A. Keen, J. Shonberg, C. J. Draper-Joyce, P. J. Scammells, A. Christopoulos, J. A. Javitch, B. Capuano, L. Shi, J. R. Lane, Molecular determinants of the intrinsic efficacy of the antipsychotic aripiprazole. *ACS Chem. Biol.* **14**, 1780–1792 (2019).

40. M. Michino, C. A. Boateng, P. Donthamsetti, H. Yano, O. M. Bakare, A. Bonifazi, M. P. Ellenberger, T. M. Keck, V. Kumar, C. Zhu, R. Verma, J. R. Deschamps, J. A. Javitch, A. H. Newman, L. Shi, Toward understanding the structural basis of partial agonism at the dopamine D_3 receptor. *J. Med. Chem.* **60**, 580–593 (2017).

41. S. Arroyo-Urea, A. L. Nazarova, Á. Carrión-Antolí, A. Bonifazi, F. O. Battiti, J. H. Lam, A. H. Newman, V. Katritch, J. García-Nafría, A bitopic agonist bound to the dopamine 3 receptor reveals a selectivity site. *Nat. Commun.* **15**, 7759 (2024).

42. A. Egyed, K. Domány-Kovács, B. Koványi, F. Horti, D. Kurkó, D. J. Kiss, G. Pándy-Szekeres, I. Greiner, G. M. Keserű, Controlling receptor function from the extracellular vestibule of G-protein coupled receptors. *Chem. Commun* **56**, 14167–14170 (2020).

43. A. Frank, D. J. Kiss, G. M. Keserű, H. Stark, Binding kinetics of cariprazine and aripiprazole at the dopamine D_3 receptor. *Sci. Rep.* **8**, 12509 (2018).

44. P. Xu, S. Huang, H. Zhang, C. Mao, X. E. Zhou, X. Cheng, I. A. Simon, D.-D. Shen, H.-Y. Yen, C. V. Robinson, K. Harpsøe, B. Svensson, J. Guo, H. Jiang, D. E. Gloriam, K. Melcher, Y. Jiang, Y. Zhang, H. E. Xu, Structural insights into the lipid and ligand regulation of serotonin receptors. *Nature* **592**, 469–473 (2021).

45. J. Shonberg, C. K. Herenbrink, L. López, A. Christopoulos, P. J. Scammells, B. Capuano, J. R. Lane, A structure-activity analysis of biased agonism at the dopamine D2 receptor. *J. Med. Chem.* **56**, 9199–9221 (2013).

46. Y. Zhuang, B. Krumm, H. Zhang, X. E. Zhou, Y. Wang, X.-P. Huang, Y. Liu, X. Cheng, Y. Jiang, H. Jiang, C. Zhang, W. Yi, B. L. Roth, Y. Zhang, H. E. Xu, Mechanism of dopamine binding and allosteric modulation of the human D1 dopamine receptor. *Cell Res.* **31**, 593–596 (2021).

47. X. Teng, S. Chen, Q. Wang, Z. Chen, X. Wang, N. Huang, S. Zheng, Structural insights into G protein activation by D1 dopamine receptor. *Sci. Adv.* **8**, eabo4158 (2022).

48. K. M. Knight, B. E. Krumm, N. J. Kapolka, W. G. Ludlam, M. Cui, S. Mani, I. Prytkova, E. G. Obarow, T. J. Lefevre, W. Wei, N. Ma, X.-P. Huang, J. F. Fay, N. Vaidehi, A. V. Smrcka, P. A. Slesinger, D. E. Logothetis, K. A. Martemyanov, B. L. Roth, H. G. Dohlman, A neurodevelopmental disorder mutation locks G proteins in the transitory pre-activated state. *Nat. Commun.* **15**, 6643 (2024).

49. V. Isberg, C. de Graaf, A. Bortolato, V. Cherezov, V. Katritch, F. H. Marshall, S. Mordalski, J.-P. Pin, R. C. Stevens, G. Vriend, D. E. Gloriam, Generic GPCR residue numbers—Aligning topology maps while minding the gaps. *Trends Pharmacol. Sci.* **36**, 22–31 (2015).

50. J. A. Ballesteros, H. Weinstein, [19] Integrated methods for the construction of three-dimensional models and computational probing of structure-function relations in G protein-coupled receptors. *Meth. Neurosci.* **25**, 366–428 (1995).

51. P. Xu, S. Huang, C. Mao, B. E. Krumm, X. E. Zhou, Y. Tan, X.-P. Huang, Y. Liu, D.-D. Shen, Y. Jiang, X. Yu, H. Jiang, K. Melcher, B. L. Roth, X. Cheng, Y. Zhang, H. E. Xu, Structures of the human dopamine D3 receptor-G_i complexes. *Mol. Cell* **81**, 1147–1159.e4 (2021).

52. P. Xu, S. Huang, B. E. Krumm, Y. Zhuang, C. Mao, Y. Zhang, Y. Wang, X.-P. Huang, Y.-F. Liu, X. He, H. Li, W. Yin, Y. Jiang, Y. Zhang, B. L. Roth, H. E. Xu, Structural genomics of the human dopamine receptor system. *Cell Res.* **33**, 604–616 (2023).

53. A. S. Dixon, M. K. Schwinn, M. P. Hall, K. Zimmerman, P. Otto, T. H. Lubben, B. L. Butler, B. F. Binkowski, T. Machleidt, T. A. Kirkland, M. G. Wood, C. T. Eggers, L. P. Encell, K. V. Wood, NanoLuc complementation reporter optimized for accurate measurement of protein interactions in cells. *ACS Chem. Biol.* **11**, 400–408 (2016).

54. M. K. Schwinn, T. Machleidt, K. Zimmerman, C. T. Eggers, A. S. Dixon, R. Hurst, M. P. Hall, L. P. Encell, B. F. Binkowski, K. V. Wood, CRISPR-mediated tagging of endogenous proteins with a luminescent peptide. *ACS Chem. Biol.* **13**, 467–474 (2018).

55. S. Maeda, A. Koehl, H. Matile, H. Hu, D. Hilger, G. F. X. Schertler, A. Manglik, G. Skiniotis, R. J. P. Dawson, B. K. Kobilka, Development of an antibody fragment that stabilizes GPCR/G-protein complexes. *Nat. Commun.* **9**, 3712 (2018).

56. X. Teng, S. Chen, Y. Nie, P. Xiao, X. Yu, Z. Shao, S. Zheng, Ligand recognition and biased agonism of the D1 dopamine receptor. *Nat. Commun.* **13**, 3186 (2022).

57. J. Yin, K.-Y. M. Chen, M. J. Clark, M. Hijazi, P. Kumari, X.-C. Bai, R. K. Sunahara, P. Barth, D. M. Rosenbaum, Structure of a D2 dopamine receptor-G-protein complex in a lipid membrane. *Nature* **584**, 125–129 (2020).

58. Y. Zhuang, P. Xu, C. Mao, L. Wang, B. Krumm, X. E. Zhou, S. Huang, H. Liu, X. Cheng, X.-P. Huang, D.-D. Shen, T. Xu, Y.-F. Liu, Y. Wang, J. Guo, Y. Jiang, H. Jiang, K. Melcher,

B. L. Roth, Y. Zhang, C. Zhang, H. E. Xu, Structural insights into the human D1 and D2 dopamine receptor signaling complexes. *Cell* **184**, 931–942.e18 (2021).

59. F. O. Battiti, S. L. Cemaj, A. M. Guerrero, A. B. Shaik, J. Lam, R. Rais, B. S. Slusher, J. R. Deschamps, G. H. Imler, A. H. Newman, A. Bonifazi, The significance of chirality in drug design and synthesis of bitopic ligands as D_3 receptor (D_3R) selective agonists. *J. Med. Chem.* **62**, 6287–6314 (2019).

60. Z. Chen, L. Fan, H. Wang, J. Yu, D. Lu, J. Qi, F. Nie, Z. Luo, Z. Liu, J. Cheng, S. Wang, Structure-based design of a novel third-generation antipsychotic drug lead with potential antidepressant properties. *Nat. Neurosci.* **25**, 39–49 (2022).

61. S. A. Gaitonde, C. Avet, M. de la Fuente Revenga, E. Blondel-Tepaz, A. Shahraki, A. M. Pastor, V. Talagayev, P. Robledo, P. Kolb, J. Selent, J. González-Maeso, M. Bouvier, Pharmacological fingerprint of antipsychotic drugs at the serotonin 5-HT_{2A} receptor. *Mol. Psychiatry* **29**, 2753–2764 (2024).

62. S. Jordan, V. Koprivica, R. Chen, K. Tottori, T. Kikuchi, C. A. Altar, The antipsychotic aripiprazole is a potent, partial agonist at the human 5-HT_{1A} receptor. *Eur. J. Pharmacol.* **441**, 137–140 (2002).

63. A. D. Stark, S. Jordan, K. A. Allers, R. L. Bertekap, R. Chen, T. M. Kannan, T. F. Molski, F. D. Yocca, T. Sharp, T. Kikuchi, K. D. Burris, Interaction of the novel antipsychotic aripiprazole with 5-HT_{1A} and 5-HT_{2A} receptors: Functional receptor-binding and in vivo electrophysiological studies. *Psychopharmacology* **190**, 373–382 (2007).

64. E. Agai-Csongor, G. Domány, K. Nógrádi, J. Galambos, I. Vágó, G. M. Keserű, I. Greiner, I. Laszlovszky, A. Gere, E. Schmidt, B. Kiss, M. Vastag, K. Tihanyi, K. Sághy, J. Laszy, I. Gyertyán, M. Zájer-Balázs, L. Gémesi, M. Kapás, Z. Szombathelyi, Discovery of cariprazine (RGH-188): A novel antipsychotic acting on dopamine D_3/D_2 receptors. *Bioorg. Med. Chem. Lett.* **22**, 3437–3440 (2012).

65. J. R. Lane, B. Powney, A. Wise, S. Rees, G. Milligan, G protein coupling and ligand selectivity of the D_{2L} and D_3 dopamine receptors. *J. Pharmacol. Exp. Ther.* **325**, 319–330 (2008).

66. D. Mönnich, L. J. Humphrys, C. Höring, B. L. Hoare, L. Forster, S. Pockes, Activation of multiple G protein pathways to characterize the five dopamine receptor subtypes using bioluminescence technology. *ACS Pharmacol. Transl. Sci.* **7**, 834–854 (2024).

67. B. M. Keegan, C. D. Vogt, L. Chen, R. J. Nosko, E. Saab, A. Bonifazi, E. S. Gogarnoiu, A. B. Shaik, A. H. Newman, L. Shi, Structure-function activity relationships of cariprazine analogues with distinct G protein versus β-arrestin activities at the dopamine D3 receptor. *ACS Chem. Nerosci.* **16**, 4679–4699 (2025).

68. N. Tsutsumi, S. Mukherjee, D. Waghray, C. Y. Janda, K. M. Jude, Y. Miao, J. S. Burg, N. G. Aduri, A. A. Kossiakoff, C. Gati, K. C. Garcia, Structure of human Frizzled5 by fiducial-assisted cryo-EM supports a heterodimeric mechanism of canonical Wnt signaling. *eLife* **9**, e58464 (2020).

69. M. A. Skiba, S. M. Sterling, S. Rawson, S. Zhang, H. Xu, H. Jiang, G. R. Nemeth, M. S. A. Gilman, J. D. Hurley, P. Shen, D. P. Staus, J. Kim, C. McMahon, M. K. Lehtinen, H. A. Rockman, P. Barth, L. M. Wingler, A. C. Kruse, Antibodies expand the scope of angiotensin receptor pharmacology. *Nat. Chem. Biol.* **20**, 1577–1585 (2024).

70. L. Fan, L. Tan, Z. Chen, J. Qi, F. Nie, Z. Luo, J. Cheng, S. Wang, Haloperidol bound D_2 dopamine receptor structure inspired the discovery of subtype selective ligands. *Nat. Commun.* **11**, 1074 (2020).

71. Y. Zhou, C. Cao, L. He, X. Wang, X. C. Zhang, Crystal structure of dopamine receptor D4 bound to the subtype selective ligand, L745870. *eLife* **8**, e48822 (2019).

72. D. Im, A. Inoue, T. Fujiwara, T. Nakane, Y. Yamanaka, T. Uemura, C. Mori, Y. Shiimura, K. T. Kimura, H. Asada, N. Nomura, T. Tanaka, A. Yamashita, E. Nango, K. Tono, F. M. N. Kadji, J. Aoki, S. Iwata, T. Shimamura, Structure of the dopamine D_2 receptor in complex with the antipsychotic drug spiperone. *Nat. Commun.* **11**, 6442 (2020).

73. S. Wang, D. Wacker, A. Levit, T. Che, R. M. Betz, J. D. McCorvy, A. J. Venkatakrishnan, X.-P. Huang, R. O. Dror, B. K. Shoichet, B. L. Roth, D_4 dopamine receptor high-resolution structures enable the discovery of selective agonists. *Science* **358**, 381–386 (2017).

74. S. Wang, T. Che, A. Levit, B. K. Shoichet, D. Wacker, B. L. Roth, Structure of the D2 dopamine receptor bound to the atypical antipsychotic drug risperidone. *Nature* **555**, 269–273 (2018).

75. B. Sun, D. Feng, M. L.-H. Chu, I. Fish, S. Lovera, Z. A. Sands, S. Kelm, A. Valade, M. Wood, T. Ceska, T. S. Kobilka, F. Lebon, B. K. Kobilka, Crystal structure of dopamine D1 receptor in complex with G protein and a non-catechol agonist. *Nat. Commun.* **12**, 3305 (2021).

76. P. Xiao, W. Yan, L. Gou, Y.-N. Zhong, L. Kong, C. Wu, X. Wen, Y. Yuan, S. Cao, C. Qu, X. Yang, C.-C. Yang, A. Xia, Z. Hu, Q. Zhang, Y.-H. He, D.-L. Zhang, C. Zhang, G.-H. Hou, H. Liu, L. Zhu, P. Fu, S. Yang, D. M. Rosenbaum, J.-P. Sun, Y. Du, L. Zhang, X. Yu, Z. Shao, Ligand recognition and allosteric regulation of DRD1-Gs signaling complexes. *Cell* **184**, 943–956.e18 (2021).

77. A. J. Venkatakrishnan, X. Deupi, G. Lebon, C. G. Tate, G. F. Schertler, M. M. Babu, Molecular signatures of G-protein-coupled receptors. *Nature* **494**, 185–194 (2013).

78. Q. Zhou, D. Yang, M. Wu, Y. Guo, W. Guo, L. Zhong, X. Cai, A. Dai, W. Jang, E. I. Shakhnovich, Z.-J. Liu, R. C. Stevens, N. A. Lambert, M. M. Babu, M.-W. Wang, S. Zhao, Common activation mechanism of class A GPCRs. *eLife* **8**, e50279 (2019).

79. M. Huang, W. He, B. Kiss, B. Farkas, N. Adham, H. Y. Meltzer, The role of dopamine D_3 receptor partial agonism in cariprazine-induced neurotransmitter efflux in rat hippocampus and nucleus accumbens. *J. Pharmacol. Exp. Ther.* **371**, 517–525 (2019).

80. M. A. Meier, C. E. Lemercier, C. Kulisch, B. Kiss, B. Lendvai, N. Adham, Z. Gerevich, The novel antipsychotic cariprazine stabilizes gamma oscillations in rat hippocampal slices. *Br. J. Pharmacol.* **177**, 1622–1634 (2020).

81. B. Levant, Characterization of dopamine receptors. *Curr. Protoc. Pharmacol.* **36**, 0106s00 (2007).

82. M. M. Bradford, A rapid and sensitive method for the quantitation of microgram quantities of protein utilizing the principle of protein-dye binding. *Anal. Biochem.* **72**, 248–254 (1976).

83. P. Seeman, H. H. Van Tol, Dopamine receptor pharmacology. *Trends Pharmacol. Sci.* **15**, 264–270 (1994).

84. D. Lévesque, J. Diaz, C. Pilon, M. P. Martres, B. Giros, E. Souil, D. Schott, J. L. Morgat, J. C. Schwartz, P. Sokoloff, Identification, characterization, and localization of the dopamine D3 receptor in rat brain using 7-[3H]hydroxy-N,N-di-n-propyl-2-aminotetralin. *Proc. Natl. Acad. Sci. U.S.A.* **89**, 8155–8159 (1992).

85. Y. Cheng, W. H. Prusoff, Relationship between the inhibition constant (K_I) and the concentration of inhibitor which causes 50 per cent inhibition (I_{50}) of an enzymatic reaction. *Biochem. Pharmacol.* **22**, 3099–3108 (1973).

86. C. Klein Herenbrink, D. A. Sykes, P. Donthamsetti, M. Canals, T. Coudrat, J. Shonberg, P. J. Scammells, B. Capuano, P. M. Sexton, S. J. Charlton, J. A. Javitch, A. Christopoulos, J. R. Lane, The role of kinetic context in apparent biased agonism at GPCRs. *Nat. Commun.* **7**, 10842 (2016).

87. J. Sanchez, Z. E. Huma, J. R. Lane, X. Liu, J. L. Bridgford, R. J. Payne, M. Canals, M. J. Stone, Evaluation and extension of the two-site, two-step model for binding and activation of the chemokine receptor CCR1. *J. Biol. Chem.* **294**, 3464–3475 (2019).

88. B. Hollins, S. Kuravi, G. J. Digby, N. A. Lambert, The c-terminus of GRK3 indicates rapid dissociation of G protein heterotrimers. *Cell. Signal.* **21**, 1015–1021 (2009).

89. C. Avet, A. Mancini, B. Breton, C. Le Gouill, A. S. Hauser, C. Normand, H. Kobayashi, F. Gross, M. Hogue, V. Lukasheva, S. St-Onge, M. Carrier, M. Héroux, S. Morissette, E. B. Fauman, J.-P. Fortin, S. Schann, X. Leroy, D. E. Gloriam, M. Bouvier, Effector membrane translocation biosensors reveal G protein and βarrestin coupling profiles of 100 therapeutically relevant GPCRs. *eLife* **11**, e74101 (2022).

90. Y. Namkung, C. LeGouill, S. Kumar, Y. Cao, L. B. Teixeira, V. Lukasheva, J. Giubilaro, S. C. Simões, J.-M. Longpré, D. Devost, T. E. Hébert, G. Piñeyro, R. Leduc, C. M. Costa-Neto, M. Bouvier, S. A. Laporte, Functional selectivity profiling of the angiotensin II type 1 receptor using pathway-wide BRET signaling sensors. *Sci. Signal.* **11**, eaat1631 (2018).

91. Y. Namkung, C. Le Gouill, V. Lukashova, H. Kobayashi, M. Hogue, E. Khoury, M. Song, M. Bouvier, S. A. Laporte, Monitoring G protein-coupled receptor and β-arrestin trafficking in live cells using enhanced bystander BRET. *Nat. Commun.* **7**, 12178 (2016).

92. J. Müller, K. Esső, G. Dargó, Á. Könczöl, G. T. Balogh, Tuning the predictive capacity of the PAMPA-BBB model. *Eur. J. Pharm. Sci.* **79**, 53–60 (2015).

93. A. Egyed, "Synthesis and evaluation of bitopic ligands targeting dopamine D2/D3 receptors," thesis, Budapest University of Technology and Economics, Budapest, Hungary (2025).

94. S. Mukherjee, S. K. Erramilli, M. Ammirati, F. J. D. Alvarez, K. F. Fennell, M. D. Purdy, B. M. Skrobek, K. Radziwon, J. Coukos, Y. Kang, P. Dutka, X. Gao, X. Qiu, M. Yeager, H. Eric Xu, S. Han, A. A. Kossiakoff, Synthetic antibodies against BRIL as universal fiducial marks for single-particle cryoEM structure determination of membrane proteins. *Nat. Commun.* **11**, 1598 (2020).

95. A. Punjani, J. L. Rubinstein, D. J. Fleet, M. A. Brubaker, cryoSPARC: Algorithms for rapid unsupervised cryo-EM structure determination. *Nat. Methods* **14**, 290–296 (2017).

96. J. Zivanov, T. Nakane, B. O. Forsberg, D. Kimanius, W. J. Hagen, E. Lindahl, S. H. Scheres, New tools for automated high-resolution cryo-EM structure determination in RELION-3. *eLife* **7**, e42166 (2018).

97. P. V. Afonine, B. K. Poon, R. J. Read, O. V. Sobolev, T. C. Terwilliger, A. Urzhumtsev, P. D. Adams, Real-space refinement in PHENIX for cryo-EM and crystallography. *Acta Crystallogr. D Struct. Biol.* **74**, 531–544 (2018).

98. E. F. Pettersen, T. D. Goddard, C. C. Huang, E. C. Meng, G. S. Couch, T. I. Croll, J. H. Morris, T. E. Ferrin, UCSF ChimeraX: Structure visualization for researchers, educators, and developers. *Protein Sci.* **30**, 70–82 (2021).

99. S. Zhang, R. H. Gumpper, X.-P. Huang, Y. Liu, B. E. Krumm, C. Cao, J. F. Fay, B. L. Roth, Molecular basis for selective activation of DREADD-based chemogenetics. *Nature* **612**, 354–362 (2022).

100. N. W. Moriarty, R. W. Grosse-Kunstleve, P. D. Adams, electronic Ligand Builder and Optimization Workbench (eLBOW): A tool for ligand coordinate and restraint generation. *Acta Crystallogr. D Biol. Crystallogr.* **65**, 1074–1080 (2009).

101. P. Emsley, K. Cowtan, Coot: Model-building tools for molecular graphics. *Acta Crystallogr. D Biol. Crystallogr.* **60**, 2126–2132 (2004).

102. M. J. Robertson, G. C. P. van Zundert, K. Borrelli, G. Skiniotis, GemSpot: A pipeline for robust modeling of ligands into cryo-EM maps. *Structure* **28**, 707–716.e3 (2020).

103. V. B. Chen, W. B. Arendall III, J. J. Headd, D. A. Keedy, R. M. Immormino, G. J. Kapral, L. W. Murray, J. S. Richardson, D. C. Richardson, MolProbity: All-atom structure validation for macromolecular crystallography. *Acta Crystallogr. D Biol. Crystallogr.* **66**, 12–21 (2010).

104. Schrödinger Release 2022-1: LigPrep, Protein Preparation Wizard. Glide, (Schrödinger LLC, 2022).

105. I. Rodríguez-Espigares, M. Torrens-Fontanals, J. K. S. Tiemann, D. Aranda-García, J. M. Ramírez-Anguita, T. M. Stepniewski, N. Worp, A. Varela-Rial, A. Morales-Pastor, B. Medel-Lacruz, G. Pándy-Szekeres, E. Mayol, T. Giorgino, J. Carlsson, X. Deupi, S. Filipek, M. Filizola, J. C. Gómez-Tamayo, A. Gonzalez, H. Gutiérrez-de-Terán, M. Jiménez-Rosés, W. Jespers, J. Kapla, G. Khelashvili, P. Kolb, D. Latek, M. Marti-Solano, P. Matricon, M.-T. Matsoukas, P. Miszta, M. Olivella, L. Perez-Benito, D. Provasi, S. Ríos, I. R. Torrecillas, J. Sallander, A. Sztyler, S. Vasile, H. Weinstein, U. Zachariae, P. W. Hildebrand, G. De

Fabritiis, F. Sanz, D. E. Gloriam, A. Cordomi, R. Guixà-González, J. Selent, GPCRmd uncovers the dynamics of the 3D-GPCRome. *Nat. Methods* **17**, 777–787 (2020).

106. S. Jo, T. Kim, V. G. Iyer, W. Im, CHARMM-GUI: A web-based graphical user interface for CHARMM. *J. Comput. Chem.* **29**, 1859–1865 (2008).

107. M. A. Lomize, I. D. Pogozheva, H. Joo, H. I. Mosberg, A. L. Lomize, OPM database and PPM web server: Resources for positioning of proteins in membranes. *Nucleic Acids Res.* **40**, D370–D376 (2012).

108. J. A. Maier, C. Martinez, K. Kasavajhala, L. Wickstrom, K. E. Hauser, C. Simmerling, Ff14SB: Improving the accuracy of protein side chain and backbone parameters from ff99SB. *J. Chem. Theory Comput.* **11**, 3696–3713 (2015).

109. J. Wang, R. M. Wolf, J. W. Caldwell, P. A. Kollman, D. A. Case, Development and testing of a general amber force field. *J. Comput. Chem.* **25**, 1157–1174 (2004).

110. C. I. Bayly, P. Cieplak, W. Cornell, P. A. Kollman, A well-behaved electrostatic potential based method using charge restraints for deriving atomic charges: The RESP model. *J. Phys. Chem.* **97**, 10269–10280 (1993).

111. M. J. Frisch, G. W. Trucks, H. B. Schlegel, G. E. Scuseria, M. A. Robb, J. R. Cheeseman, G. Scalmani, V. Barone, G. A. Petersson, H. Nakatsuji, X. Li, M. Caricato, A. V. Marenich, J. Bloino, B. G. Janesko, R. Gomperts, B. Mennucci, H. P. Hratchian, J. V. Ortiz, A. F. Izmaylov, J. L. Sonnenberg, D. Williams-Young, F. Ding, F. Lipparini, F. Egidi, J. Goings, B. Peng, A. Petrone, T. Henderson, D. Ranasinghe, V. G. Zakrzewski, J. Gao, N. Rega, G. Zheng, W. Liang, M. Hada, M. Ehara, K. Toyota, R. Fukuda, J. Hasegawa, M. Ishida, T. Nakajima, Y. Honda, O. Kitao, H. Nakai, T. Vreven, K. Throssell, J. A. Montgomery Jr, J. E. Peralta, F. Ogliaro, M. J. Bearpark, J. J. Heyd, E. N. Brothers, K. N. Kudin, V. N. Staroverov, T. A. Keith, R. Kobayashi, J. Normand, K. Raghavachari, A. P. Rendell, J. C. Burant, S. S. Iyengar, J. Tomasi, M. Cossi, J. M. Millam, M. Klene, C. Adamo, R. Cammi, J. W. Ochterski, R. L. Martin, K. Morokuma, O. Farkas, J. B. Foresman, D. J. Fox, Gaussian 16 Revision C.01. Gaussian Inc. (2016).

112. D. Case, H. M. Aktulga, K. Belfon, I. Y. Ben-Shalom, J. T. Berryman, S. R. Brozell, D. S. Cerutti, T. E. Cheatham, G. G. Iii, V. W. D. Cisneros, T. Cruzeiro, R. E. Darden, G. Duke, M. K. Giambasu, H. Gilson, W. Gohlke, R. Goetz, S. Harris, S. Izadi, K. Izmailov, M. C. Kasavajhala, E. Kaymak, T. Kovalenko, T. S. Kurtzman, S. Lee, P. Legrand, C. Li, J. Lin, T. Liu, R. Luchko, M. Luo, V. Machado, M. Man, K. M. Manathunga, Y. Merz, O. Miao, G. Mikhailovskii, H. Monard, K. Nguyen, F. Onufriev, S. Pan, R. Pantano, D. R. Rahnamoun, C. Roitberg, S. Sagui, J. Shajan, C. L. Shen, N. R. Simmerling, J. Skrynnikov, J. Smith, R. C. Swails, J. Walker, J. Wang, H. Wang, R. M. Wei, X. Wolf, Y. Wu, Y. Xiong, D. M. Xue, S. York, P. Zhao, P. A. Kollmann, Amber 2022. University of California, San Francisco (2022).

113. B. Leimkuhler, C. Matthews, Rational construction of stochastic numerical methods for molecular sampling. *Appl. Math. Res. Express* **2013**, 34–56 (2012).

114. J.-P. Ryckaert, G. Ciccotti, H. J. C. Berendsen, Numerical integration of the cartesian equations of motion of a system with constraints: Molecular dynamics of *n*-alkanes. *J. Comput. Phys.* **23**, 327–341 (1977).

115. S. Miyamoto, P. A. Kollman, Settle: An analytical version of the SHAKE and RATTLE algorithm for rigid water models. *J. Comput. Chem.* **13**, 952–962 (1992).

116. C. Lu, C. Wu, D. Ghoreishi, W. Chen, L. Wang, W. Damm, G. A. Ross, M. K. Dahlgren, E. Russell, C. D. Von Bargen, R. Abel, R. A. Friesner, E. D. Harder, OPLS4: Improving force field accuracy on challenging regimes of chemical space. *J. Chem. Theory Comput.* **17**, 4291–4300 (2021).

117. J.-M. Chang, P. Di Tommaso, J.-F. Taly, C. Notredame, Accurate multiple sequence alignment of transmembrane proteins with PSI-Coffee. *BMC Bioinformatics* **13**, S1 (2012).
